# The Chironomidae (Diptera) of Svalbard and Jan Mayen

**DOI:** 10.3390/insects11030183

**Published:** 2020-03-13

**Authors:** Elisabeth Stur, Torbjørn Ekrem

**Affiliations:** Department of Natural History, NTNU University Museum, Norwegian University of Science and Technology, NO-7491 Trondheim, Norway; torbjorn.ekrem@ntnu.no

**Keywords:** non-biting midges, arctic, DNA barcodes, taxonomy, biogeography, distribution, identification keys

## Abstract

Non-biting midges of the fly family Chironomidae are extremely abundant and diverse in Arctic regions and are essential components of Arctic ecosystems. Modern identification tools based on documented records of Arctic chironomid species are therefore important for ecological research and environmental monitoring in the region. Here, we provide an updated review of the chironomid fauna of the Svalbard archipelago and the island of Jan Mayen, Norway. Our results show that a total of 73 species distributed across 24 genera in four subfamilies are known from these areas. Our review treats 109 taxa, including nomina dubia and misidentifications. It includes morphological identification keys to all known species as well as photographs of most taxa and DNA barcodes of 66 species. Taxonomic remarks are given for selected taxa, including previous misidentifications and erroneous records. *Chironomus islandicus*, *Tvetenia bavarica*, *Limnophyes schnelli*, *Metriocnemus brusti* and *Metriocnemus fuscipes* as well as the genera *Allocladius*, *Corynoneura* and *Bryophaenocladius* are reported from Svalbard for the first time, while *Procladius* (*Holotanypus*) *frigidus*, *Stictochironomus psilopterus*, *Chaetocladius incertus*, *Orthocladius* (*Orthocladius*) *mixtus* and *Smittia longicosta*, previously considered as junior synonyms or nomina dubia, are revived as valid species based on examination of type material or literature. Twenty species within eleven genera are introduced with interim names. *Metriocnemus similis* is regarded as a junior synonym of *Metriocnemus ursinus*, and *Smittia incerta*, *Smittia flexinervis* and *Smittia spitzbergensis* are regarded as nomina dubia. Valid taxa no longer considered as part of the Svalbard fauna are *Parochlus kiefferi*, *Arctopelopia barbitarsis*, *Procladius* (*Holotanypus*) *crassinervis*, *Diamesa lindrothi*, *Diamesa incallida*, *Diamesa lundstromi*, *Chironomus hyperboreus*, *Sergentia coracina*, *Camptocladius stercorarius*, *Chaetocladius dissipatus*, *Chaetocladius dentiforceps*, *Chaetocladius laminatus*, *Chaetocladius perennis*, *Cricotopus* (*Cricotopus*) *humeralis*, *Cricotopus* (*Cricotopus*) *polaris*, *Hydrosmittia ruttneri*, *Limnophyes edwardsi*, *Metriocnemus picipes*, *Metriocnemus tristellus*, *Orthocladius* (*Eudactylocladius*) *gelidus*, *Orthocladius* (*Euorthocladius*) *thienemanni*, *Orthocladius* (*Orthocladius*) *obumbratus*, *Orthocladius* (*Orthocladius*) *rhyacobius*, *Paralimnophyes*, *Paraphaenocladius impensus*, *Psectrocladius* (*Monopsectrocladius*) *calcaratus*, *Psectrocladius* (*Psectrocladius*) *psilopterus*, *Psectrocladius* (*Psectrocladius*) *ventricosus*, *Smittia lasiophthalma*, *Smittia lasiops* and *Zalutschia tatrica*.

## 1. Introduction

The family Chironomidae, or non-biting midges, is one of the most common and species rich organism groups in freshwater and semi-aquatic habitats [1]. It has members in all biogeographical regions, including the Antarctic mainland, and more than 6000 valid species described world-wide ([2,3]; Patrick Ashe pers. comm.). As is true for most insect groups, chironomids are considered as better known in some regions than in others. However, even in regions with long taxonomic history, new species are frequently discovered (e.g., [4]). This is at least partly due to the fact that molecular work, especially DNA barcoding [5], has become more common, and enabled researchers to detect morphologically similar species with distinct genetic lineages [4,6,7,8,9]. DNA barcodes can also aid in life stage association [10]; an important asset in freshwater paleoecology where it is challenging to retrieve DNA from pre-historic samples. Thus, by using DNA barcodes to associate larvae with morphologically identifiable adults or pupae, one does not depend on the rearing of larvae for species-level identifications. Rearing larvae to emerging adults can be challenging for species with strict environmental requirements.

Chironomids are extremely frequent and diverse in the Arctic. In fact, 360 species have been recorded with certainty and an estimate of more than 700 species exist [11]. Although this estimate is likely too high, as it is based on extrapolation of the Chironominae diversity in the Holarctic Region (published world catalogues only available for other subfamilies), the chironomid diversity of the Arctic surpasses that of all other comparable groups of invertebrates. In extreme high Arctic regions, chironomids can comprise up to half of all insect species [12] and at lower Arctic latitudes, they also represent a considerable share of the diversity [13,14,15]. Yet, gaps in the taxonomic knowledge of Arctic Chironomidae are still acknowledged [16], and as seen in the present study, any thorough collection event will likely record species new to science and species belonging to groups in need of revision.

The abundance of chironomids in both terrestrial and freshwater habitats makes them important components of many Arctic food webs [17]. In a study conducted at Zackenberg, eastern Greenland, dipterans were found to completely dominate the community of flying insects cought in a Malaise trap (97%), and 42% of these Diptera were chironomids [18]. It is reasonable to assume that similar numbers occur at sites with comparable environments. Moreover, in freshwater habitats, midge larvae are an important food source for fish, e.g., for juvenile and dwarf Arctic char (*Salvelinus alpinus*) (e.g., [19]). The importance of chironomid species with terrestrial immature stages in the Arctic is insufficiently explored, but Chironomidae larvae are often encountered when extracting invertebrates from soil [20].

**Figure 1:**
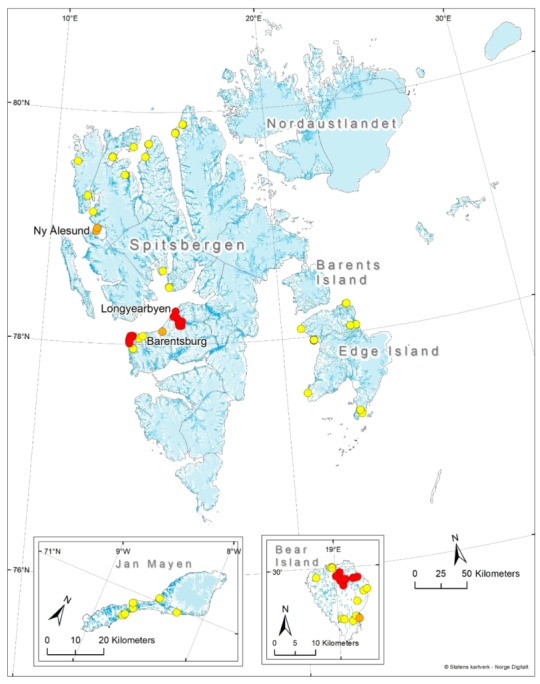
Map of Svalbard and Jan Mayen showing localities sampled for this study. Red = three or more visits per locality, orange = two visits, yellow = one visit.

The archipelago of Svalbard is located between 74° and 81° N and between 10° and 35° E, in the Barents Sea north of mainland Norway (Figure 1). It has been under Norwegian sovereignty since the effectuation of the Svalbard Treaty in 1925. Svalbard consists of the five main islands Spitsbergen, Nordaustlandet (North East Land), Edgeøya (Edge Island), Barentsøya (Barents Island) and Bjørnøya (Bear Island) in addition to numerous smaller islands, islets and skerries. The first four principal islands are grouped more or less together north of 76° latitude, while Bear Island is located further south at about 74.3° N approximately halfway between the Norwegian mainland and Spitsbergen. The archipelago lies within the high Arctic as defined by Conservation of Arctic Flora and Fauna [21]. More than 60% of the approximately 61,000 km^2^ land mass is permanently covered by ice and snow, while less than 10% is covered by vegetation [22]. The geological history of Svalbard is relatively complex with bedrock formation at different times in geological history [23].

The volcanic Jan Mayen Island is situated 550 km northeast of Iceland between 72.0° and 75.1° N and between 7.1° and 8.1° W. It is not part of Svalbard and has a completely different geological history as well as administrative organization. The island covers about 377 km^2^ and is dominated by the 2277 m tall Beerenberg volcano [24]. Both the geographical position of the island and its Arctic-maritime climate make the biota of Jan Mayen interesting in a biogeographical and environmental perspective. Jan Mayen has been part of Norway since 1930.

The first chironomids to be documented from Svalbard were collected by expeditions between 1838 and 1861 and described by Carl Henrik Boheman [25]. Five chironomid species were recorded in the material available to him, of which three are recognised as part of the Svalbard fauna today, either under a senior synonym (*Chironomus lugubris* Zetterstedt, 1850 = *C. polaris* Boheman, 1866) or subsequently placed in different genera (*Diamesa arctica* (Boheman, 1866) and *Smittia brevipennis* (Boheman, 1866)) (Species were described in Boheman (1865), but names were not available according to the International Code of Zoological Nomenclature until actual publication in 1866.) Concering his remaining two species identifications, one used a name currently regarded as a nomen dubium (*Tanytarsus productus* (Zetterstedt, 1838)) and the other likely was a misidentification of *Smittia aterrima* (Meigen, 1818). The list of species from Spitsbergen was revised and increased by August Emil Holmgren who participated in the Swedish expedition to the archipelago in 1868. The expedition was able to land on Bear Island in July-August, a favourable time of the year for collecting flying insects (landing on Bear Island to collect insects was not common for contemporary expeditions). In the following publication, Holmgren listed twenty-one chironomid species from Svalbard including eight species from Bear Island [26]. Sixteen of the species were described as new to science, and nine of these are still regarded as valid first descriptions. Later publications that considerably increased the number of species from the archipelago were those by Jean-Jacques Kieffer and August Thienemann [27] based on collections made by Albert Koch, and by Frederick Wallace Edwards [28,29,30,31,32], who examined material collected by various British expeditions. Seven species were later added by Mauri Hirvenoja [33], but three of these records turned out to be misidentifications when compared with the current species concepts (see below), and two are considered as junior synonyms of older names assigned to species distributed in Svalbard.

The chironomid fauna of Jan Mayen has not been investigated to the same extent. The first records were published by Eduard [34], who described two species new to science: *Chironomus incertus* Becher, 1886 now regarded as a junior synonym of *Smittia extrema* (Holmgren, 1869) and *Chironomus callosus* Becher, 1886, here regarded as a junior synonym of *Metriocnemus ursinus* (Holmgren, 1869). Later records from the island were treated by Edwards [35] who recognized seven different species, but labelled several of his identifications as doubtful.

Modern-day use of Chironomidae in Arctic ecological studies needs up-to-date identification tools to arrive reliable or plausible identifications. Moreover, consistency in identifications, both over time and between studies, is needed in order to make ecological studies comparable and to monitor diversity change through time. A shared perception of species, which is crucial to interpret biodiversity data correctly, can be difficult to obtain from morphology alone. Thus, the use of molecular tools for identification of Chironomidae adds objectivity and comparability in classifications. Identification of species based on short, standardized gene fragments, the so-called DNA barcoding [5], has proven useful in this regard as it adds objectivity to identifications and works equally well for all life stages [10]. Moreover, as biological monitoring already takes advantage of molecular tools such as metabarcoding and metagenomics [36,37,38,39], it is reasonable to believe that this also will be the case for future biomonitoring in the Arctic. However, identifications through DNA barcoding cannot ever be better than the reference library upon which they are based [40] and keys and descriptions based on morphology will continue to be valuable assets of the chironomid literature. This applies especially to identifications of material from which it is difficult to obtain high quality DNA, such as to subfossil head capsules, historical material, and specimens fixed in DNA-damaging preservatives.

The aim of this study, therefore, was to provide a revised overview of Svalbard’s and Jan Mayen’s Chironomidae faunas, and to present identification keys and associated DNA barcodes for as many species as possible. It is not our intention to perform taxonomic revisions, but we do discuss taxonomic issues that we detected and/or resolved during our observations and examinations of available material.

## 2. Materials and Methods

Chironomidae specimens used were collected through nine field trips to Spitsbergen and Bear Island in the Arctic summer from mid June to mid August in the years 2002–2013. Adults were collected by Malaise traps, sweep nets, pitfall traps and emergence traps, while immatures were collected with drift nets, kick sampling and Eckman grab samplers. In total, 92 localities were sampled by us or colleagues (Figure 1).

Thousands of specimens were sorted through in order to select a representative number of specimens for each species. Adult specimens were preserved in 85% ethanol, while immatures were preserved in 96% ethanol. Sorting morphospecies was conducted under a stereo microscope, while species identification usually was done on slide-mounted material in a compound microscope. Slide mounts were made using Euparal and in accordance with Pinder [41]. All specimens are deposited in the Natural History Collections of the NTNU University Museum in Trondheim, Norway (NTNU-VM). Photographs were taken with a Leica DM6000 microscope under various light conditions using a Leica DFC 420 camera and the Multifocus module in the software Leica Application Suite 4.8.

For the 944 specimens subjected to DNA analysis, tissue was sampled prior to slide mounting and shipped to the Canadian Centre for DNA Barcoding (CCDB) at the University of Guelph through the collaboration with the Norwegian Barcode of Life Network. DNA extraction followed standard protocols for insect tissues at CCDB, PCR and bi-directional Sanger sequencing used either the LCO1490 and HCO2198 primers [42] or the LepF1 + LepR1 primers [43] or a cocktail of these (C_LepFolF and C_LepFolR, [44]).

The DNA barcodes, Barcode Index Numbers (BINs), GenBank accessions and associated meta-data, including specimen and collection information of the Svalbard and Jan Mayen Chironomidae referred to in this study are available through the public dataset DS-CHIRSV (doi:10.5883/DS-CHIRSV) in the Barcode of Life Data Systems (BOLD) [45].

The literature used for morphological identification of the material comprise taxonomic revisions [10,46,47,48,49,50,51,52,53,54,55,56,57,58,59,60,61,62] as well as original or re-descriptions [25,26,28,31,63,64,65,66]. In particular, the keys to Holarctic Chironomidae [67,68,69] have been useful to consult diagnostic characters on generic level.

## 3. Results

In total, 73 species are regarded as documented inhabitants of Svalbard and/or Jan Mayen (Table 1). Among these, 60 are known from Spitsbergen, 10 from Edgeøya, 32 from Bear Island, and eight from Jan Mayen. Fifty-four species currently known from the islands can be associated with Linnean names, the rest being separable morphological species with interim names (Table 1). These are either species not yet formally described or belong to genera in need of taxonomic revision before the identity of the specimens we have examined can be determined.

As a result of our review, eight taxa are reported from Svalbard from the first time: *Chironomus islandicus* (Kieffer, 1913), *Limnophyes schnelli* Sæther, 1990, *Metriocnemus brusti* Sæther, 1989, *Metriocnemus fuscipes* (Meigen, 1818) and *Tvetenia bavarica* (Goetghebuer, 1934) as well as the genera *Allocladius* Kieffer, 1913, *Bryophaenocladius* Thienemann, 1934 and *Corynoneura* Winnertz, 1846. On the other hand, we regard the previously reported species *Parochlus kiefferi* (Garrett, 1925), *Arctopelopia barbitarsis* (Zetterstedt, 1850), *Procladius* (*Holotanypus*) *crassinervis* Zetterstedt, 1838, *Diamesa incallida* (Walker, 1856), *Diamesa lindrothi* Goetghebuer, 1931, *Diamesa lundstromi* Kieffer 1918, *Chironomus hyperboreus* Stæger, 1845, *Sergentia coracina* (Zetterstedt, 1850), *Camptocladius stercorarius* (De Geer, 1776), *Chaetocladius dentiforceps* (Edwards, 1929), *Chaetocladius dissipatus* (Edwards, 1929), *Chaetocladius laminatus* Brundin, 1947, *Chaetocladius perennis* (Meigen, 1830), *Cricotopus* (*Cricotopus*) *humeralis* (Zetterstedt, 1838), *Cricotopus* (*Cricotopus*) *polaris* Kieffer, 1926, *Hydrosmittia ruttneri* (Strenzke and Thienemann, 1942), *Limnophyes edwardsi* Sæther, 1990, *Metriocnemus picipes* (Meigen, 1818), *Metriocnemus tristellus* Edwards, 1929, *Orthocladius* (*Eudactylocladius*) *gelidus* (Kieffer, 1922), *Orthocladius* (*Euorthocladius*) *thienemanni* Kieffer, 1906, *Orthocladius* (*Orthocladius*) *obumbratus* Johannsen, 1905, *Orthocladius* (*Orthocladius*) *rhyacobius* Kieffer, 1911, *Paralimnophyes* Brundin, 1956, *Paraphaenocladius impensus* (Walker, 1856), *Psectrocladius* (*Monopsectrocladius*) *calcaratus* (Edwards, 1929), *Psectrocladius* (*Psectrocladius*) *psilopterus* (Kieffer, 1906), *Psectrocladius* (*Psectrocladius*) *ventricosus* Kieffer, 1925, *Smittia lasiophthalma* (Malloch, 1915), *Smittia lasiops* (Malloch, 1915) and *Zalutschia tatrica* (Pagast, 1935) to be erroneous records based on misidentifications or misconceptions. The species *Smittia flexinervis* (Kieffer, 1911), *Smittia incerta* (Becher, 1886) and *Smittia spitzbergensis* (Kieffer, 1919) are regarded as nomina dubia, while *Metriocnemus similis* Kieffer, 1922 is regarded as a junior synonym of *Metriocnemus ursinus* (Holmgren, 1869).

We present novel DNA barcodes for 66 species from Svalbard and Jan Mayen that are associated with morphological groups and compared with DNA barcode data of related populations and taxa, also from regions outside of the Arctic. For fifty-eight species recorded from Svalbard there are DNA barcodes from other regions (Table 1). Through the use of DNA barcodes, we associated immature life stages of 61 species, several of which were previously undescribed. This led to a few interesting findings such as the premandibular structure in larvae of *Chaetocladius incertus* (Lundström, 1915), previously regarded as a junior synonym of *Chaetocladius perennis* (see discussion below).

### 3.1. Keys to the Chironomidae of Svalbard and Jan Mayen

The key to adults includes species that are recorded from Svalbard and Jan Mayen. It is likely that more species from certain genera will be found in the future and caution should be taken in species-level identifications. Although we do not know of reliable records, we included the genera *Parochlus*, *Paralimnophyes* and *Sergentia* in the key of these because they are not unlikely to be found on Svalbard or Jan Mayen. The keys to larvae and pupae are to genus-level only since many species have unknown immature stages and keys could therefore be misleading. Since the characters used in the key to immatures are based on known associations, they might not represent the species on Svalbard very well (e.g., *Paraphaenocladius*).

#### 3.1.1. Adults

**Figure 2:**
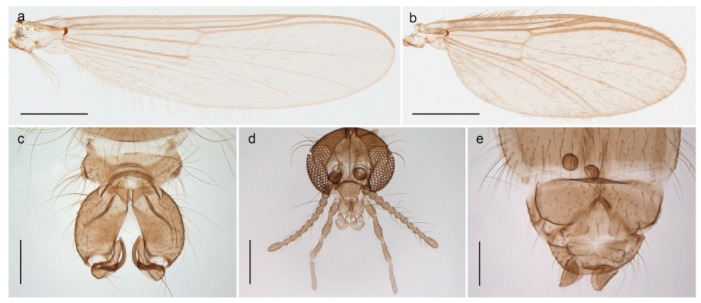
*Parochlus kiefferi* (specimens from continental Norway). (**a**) Male wing, scale bar = 500 µm; (**b**) female wing, scale bar = 500 µm; (**c**) male hypopygium, scale bar = 100 µm; (**d**) male head, scale bar = 200 µm; (**e**) female genitalia, scale bar = 100 µm.

**Figure 3:**
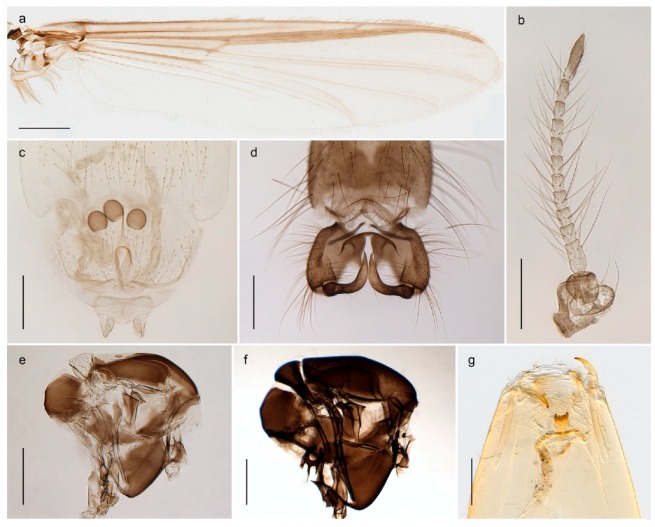
*Arctopelopia melanosoma*. (**a**) Male wing, scale bar = 500 µm; (**b**) female antenna, scale bar = 200 µm; (**c**) female genitalia, scale bar = 200 µm; (**d**) male hypopygium, scale bar = 200 µm; (**e**) female thorax, scale bar = 500 µm; (**f**) male thorax, scale bar = 500 µm; (**g**) larval head, scale bar = 200 µm.

**Figure 4:**
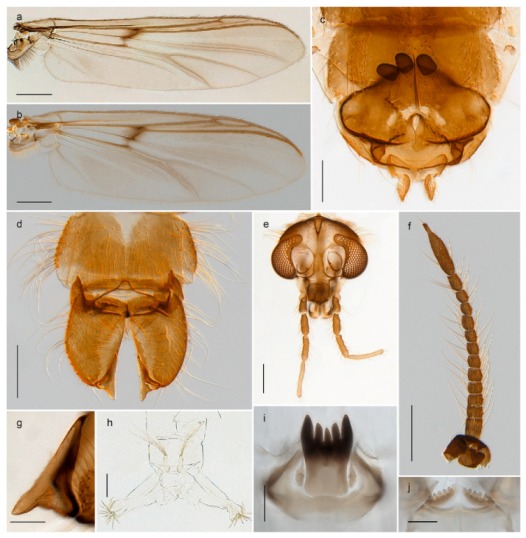
*Procladius* (*Holotanypus*) *frigidus*. (**a**) Male wing, scale bar = 500 µm; (**b**) female wing, scale bar = 500 µm; (**c**) female genitalia, scale bar = 200 µm; (**d**) male hypopygium, scale bar = 200 µm; (**e**) female head, scale bar = 200 µm; (**f**) female antenna, scale bar = 200 µm; (**g**) tooth of gonostylus, scale bar = 50 µm; (**h**) larva, posterior end, scale bar = 500 µm; (**i**) larval ligula, scale bar = 50 µm; (**j**) larval mentum, scale bar = 50 µm.

**Figure 5:**
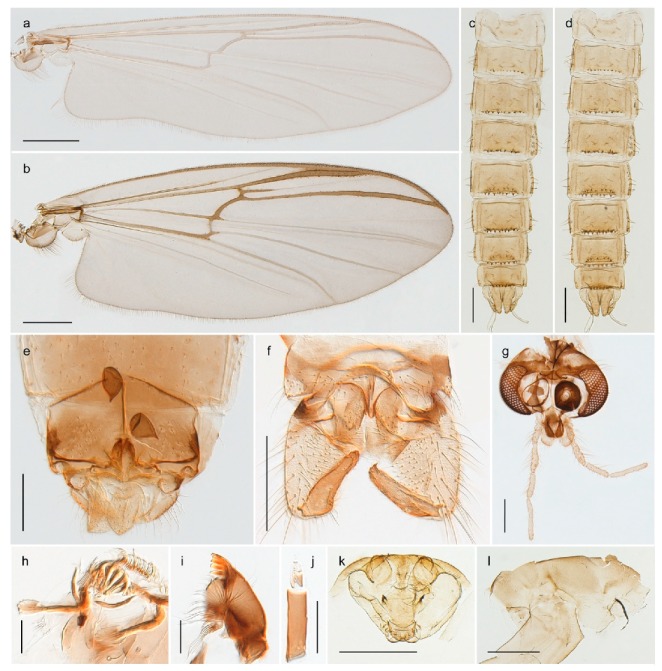
*Diamesa aberrata*. (**a**) Male wing, scale bar = 500 µm; (**b**) female wing, scale bar = 500 µm; (**c**) pupal abdomen, dorsal view, scale bar = 500 µm; (**d**) pupal abdomen, ventral view, scale bar = 500 µm; (**e**) female genitalia, scale bar = 200 µm; (**f**) male hypopygium, scale bar = 200 µm; (**g**) male head, scale bar = 200 µm; (**h**) larval labral surface, scale bar = 50 µm; (**i**) larval mandible, scale bar = 50µm; (**j**) larval antenna, scale bar = 50 µm; (**k**) pupal frontal apotome, scale bar = 500 µm; (**l**) pupal thorax, scale bar = 500 µm.

1. Wing with crossvein MCu present (e.g., Figures 2a,b, 3a and 4a,b)
2Wing with crossvein MCu absent (e.g., Figures 13a and 23a)
152. Wing vein R_2+3_ absent (Figure 2a,b)
*Parochlus kiefferi*
- Wing vein R_2+3_ present (Figures 4a,b and 5a,b)
33. Wing vein R_2+3_ forked (Figure 4a,b); tarsomere 4 cylindrical (subfamily Tanypodinae) 4- Wing vein R2+3 simple (Figure 5a,b); tarsomere 4 cordiform (Figures 8f and 10b) (subfamily Diamesinae)
54. Wing with crossvein MCu ending in M3+4 distal to cubital fork (Figure 3a)

*Arctopelopia melanosoma*
- Wing with crossvein MCu ending in Cu, proximal to cubital fork (Figure 4a,b)
*Procladius* (*Holotanypus*) *frigidus*5. Microtrichia present between all ommatidia in of the eye, giving a «hairy» appearance (Genus Diamesa)
6- Microtrichia only present between ommatidia near inner margin of the eye (Figure 10e)
*Pseudokiefferiella* sp. 1ES6. Outer genitalia with well-developed gonocoxites and mobile gonostyli (e.g., Figure 5f) (males)
7- Outer genitalia with reduced gonocoxites and one-segmented cerci (e.g., Figure 5e) (females)
117. Anal point of hypopygium small (Figure 5f)

**Figure 6:**
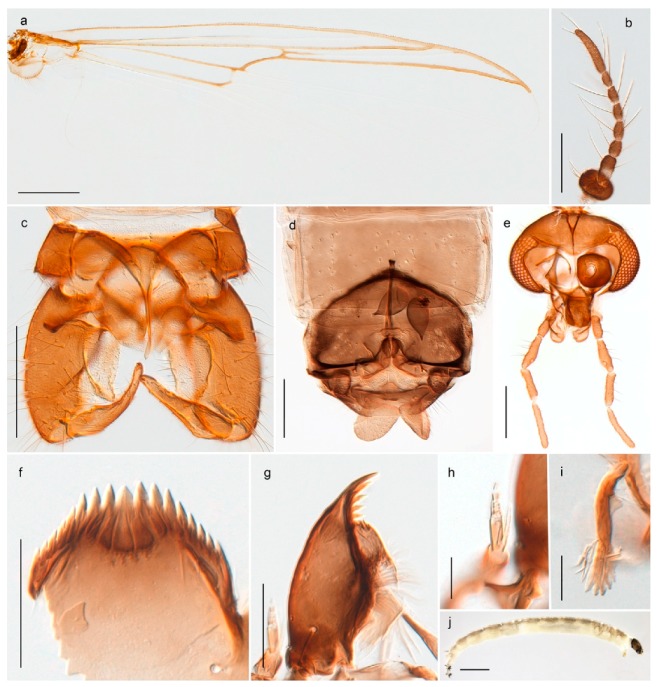
*Diamesa arctica*. (**a**) Male wing, scale bar = 500 µm; (**b**) female antenna, scale bar = 200 µm; (**c**) male hypopygium, scale bar = 200 µm; (**d**) female genitalia, scale bar = 200 µm; (**e**) male head, scale bar = 200 µm; (**f**) larval mentum, scale bar = 50 µm; (**g**) larval mandible, scale bar = 50 µm; (**h**) larval antenna, scale bar = 20 µm; (**i**) larval premandible, scale bar = 20 µm; (**j**) larva, scale bar = 1 mm.

*Diamesa aberrata*
- Anal point of hypopygium well-developed (e.g., Figure 6c)
88. Gonocoxite 2–3 times longer than gonostylus (Figure 8a)

**Figure 7:**
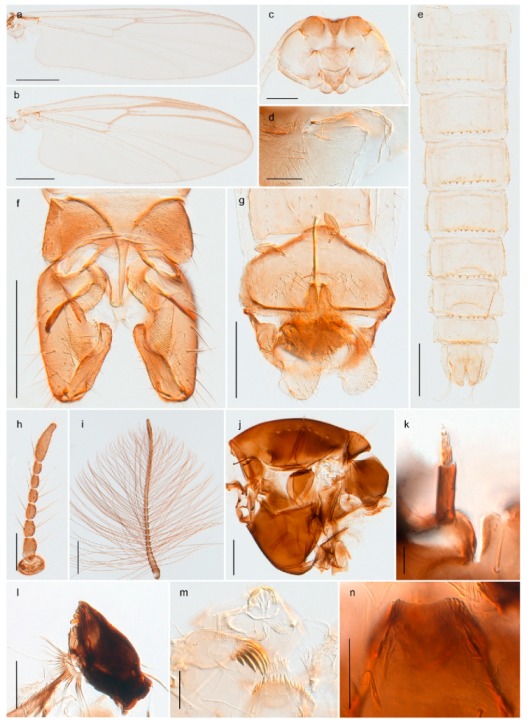
*Diamesa bertrami*. (**a**) Male wing, scale bar = 500 µm; (**b**) female wing, scale bar = 500 µm; (**c**) pupal frontal apotome, scale bar = 200 µm; (**d**) pupal thoracic horn, scale bar = 100 µm; (**e**) pupal abdomen, dorsal view, scale bar = 500 µm; (**f**) male hypopygium, scale bar = 200 µm; (**g**) female genitalia, scale bar = 200 µm; (**h**) female antenna, scale bar = 100 µm; (**i**) male antenna, scale bar = 200 µm; (**j**) thorax, scale bar = 200 µm; (**k**) larval antenna, scale bar = 20 µm; (**l**) larval mandible, scale bar = 50µm; (**m**) larval mandible, scale bar = 50 µm; (**n**) larval mentum, scale bar = 50 µm.

*Diamesa bohemani*
- Gonocoxite <2 times longer than gonostylus (e.g., Figure 7f)
99. Anal point of hypopygium broadly triangular (Figure 9a); antenna with reduced plume (Figure 9f)

*Diamesa hyperborea*
- Anal point of hypopygium long, thin (e.g., Figure 7f); antenna with normally developed plume (e.g., Figure 7i)
1010. Anal point of hypopygium with apical tooth; inner margin of gonostylus strongly concave in apical half (Figure 7f)

*Diamesa bertrami*
- Anal point of hypopygium without apical tooth; inner margin of gonostylus slightly concave and tapering towards apex (Figure 6c)

**Figure 8:**
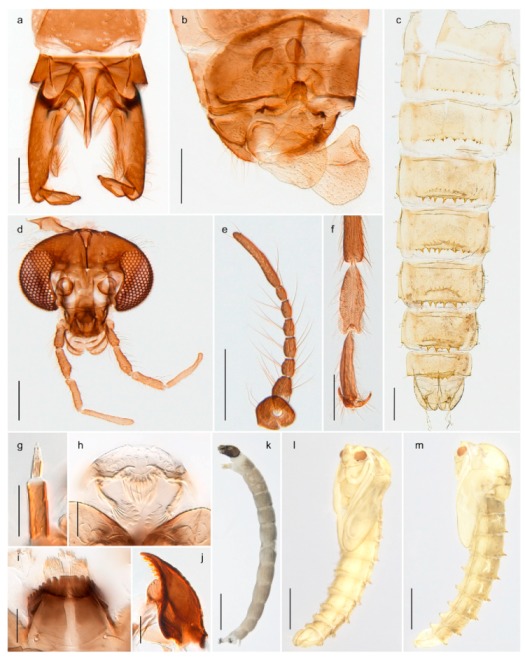
*Diamesa bohemani*. (**a**) Male hypopygium, scale bar = 200 µm; (**b**) female genitalia, scale bar = 200 µm; (**c**) pupal abdomen, scale bar = 500 µm; (**d**) female head, scale bar = 200 µm; (**e**) female antenna, scale bar = 200 µm; (**f**) male tarsus, scale bar = 100 µm; (**g**) larval antenna, scale bar = 50 µm; (**h**) larval labral surface, scale bar = 50 µm; (**i**) larval mentum, scale bar = 50 µm; (**j**) larval mandible, scale bar = 50 µm; (**k**) larva, scale bar = 1 mm; (**l**) pupa ventral view, scale bar = 1 mm; (**m**) pupa lateral view, scale bar = 1 mm.

*Diamesa arctica*
11. Pseudospurs present on tarsomere 3 of all legs; cercus as large, or larger than segment IX, with apical constriction ventrally (Figure 8b)

**Figure 9:**
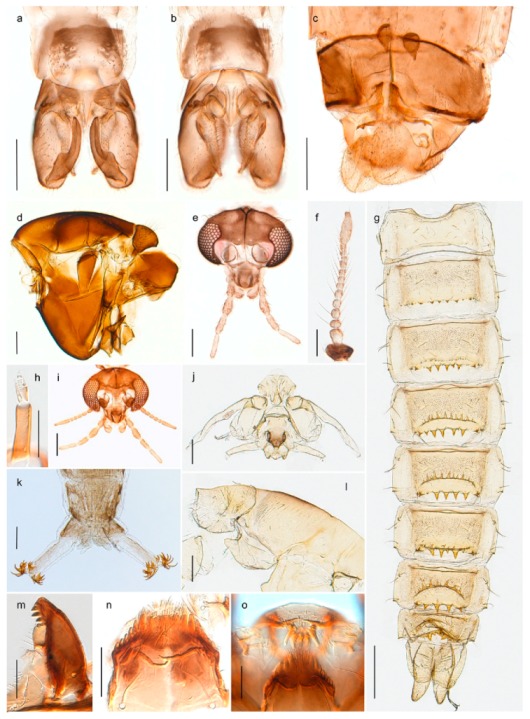
*Diamesa hyperborea*. (**a**) Male hypopygium dorsal view, scale bar = 200 µm; (**b**) Male hypopygium ventral view, scale bar = 200 µm; (**c**) female genitalia ventral view, scale bar = 200 µm; (**d**) female thorax, scale bar = 200 µm; (**e**) male head, scale bar = 200 µm; (**f**) male antenna, scale bar = 100 µm; (**g**) pupal abdomen dorsal view, scale bar = 500 µm; (**h**) larval antenna, scale bar = 50 µm; (**i**) female head, scale bar = 200 µm; (**j**) pupal frontal apotome, scale bar = 200 µm; (**k**) larva posterior parapods, scale bar = 200 µm; (**l**) pupal thorax, scale bar = 200 µm; (**m**) larval mandible, scale bar = 50 µm; (**n**) larval mentum, scale bar = 50 µm; (**o**) larval labral surface, scale bar = 50 µm.

**Figure 10:**
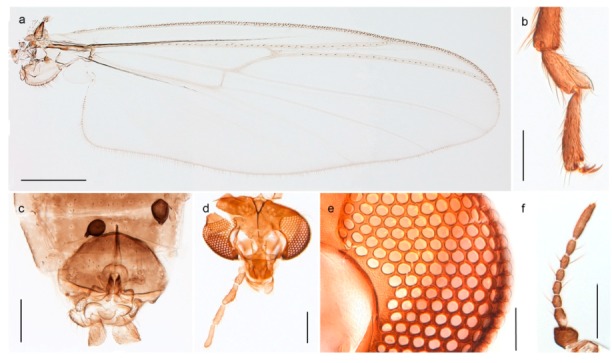
*Pseudokiefferiella* sp. (**a**) Female wing, scale bar = 500 µm; (**b**) female tarsus, scale bar = 100 µm; (**c**) female genitalia, scale bar = 200 µm; (**d**) female head, scale bar = 200 µm; (**e**) female eye, scale bar = 50 µm; (**f**) female antenna, scale bar = 200 µm.

*Diamesa bohemani*
- Pseudospurs absent on tarsomere 3 of all legs; cercus smaller and of different shape (e.g., Figure 9c)
1212. Pseudospurs present on tarsomere 1–2 of fore leg; cerci broadly triangular in lateral view (Figure 9c)

*Diamesa hyperborea*
- Pseudospurs absent on tarsomere 1–2 on fore leg; cercus of different shape (e.g., Figure 6d)
1313. Eye hairy; cercus with obvious ventral elongation (Figure 7g)

*Diamesa bertrami*
- Eye pubescent; cercus without obvious ventral elongation (Figures 5e and 6d)
1414. Gonapophysis VIII with angular medioposterior corner (Figure 5e)

*Diamesa aberrata*
- Gonapophysis VIII with rounded medioposterior corner (Figure 6d)

*Diamesa arctica*
15. Fore tarsomere 1 longer than fore tibia; tibial comb of hind leg consisting of fused spines (e.g., Figures 12i and 15b) (subfamily Chironominae)
16- Fore tarsomere 1 shorter than fore tibia; tibial comb of hind leg consisting of free spiniform setae (e.g., Figures 23i and 25j) (subfamily Orthocladiinae)
3216. Wing membrane with macrotrichia; squama bare; crossvein RM parallel with R4+5 and continuous with it (e.g., Figures 13a and 14a,b) (tribe Tanytarsini)
17- Wing membrane often bare, squama with numerous setae on edge; crossvein RM oblique with R4+5 (e.g., Figures 16a, 19a and 20a) (tribe Chironomini)
2517. Antenna short with 5–6 flagellomeres; genitalia with cerci, without strongly developed gonocoxites and gonostyli (females)
18- Antenna plumose with 11 flagellomeres; genitalia with strongly developed gonocoxites and gonostyli (males)
2218. Small, bright green in colour with brown mesonotal bands; fore leg ratio (LR1) >1.4; mid-and hind tibial combs well separated, each with obvious spur; gonapophysis VIII undivided; parthenogenetic on Svalbard (Figure 15)

*Tanytarsus heliomesonyctios*
- Dark specimens, if greenish in ground colour, always with some brown pigmentation (not bright green); LR1 < 1.4; mid- and hind tibial combs fused (e.g., Figure 12i), without or with at most a minute spur; gonapophysis VIII divided
1919. Light olive green ground colour, scutellum and antennae; mid and hind tibial combs with minute spur; dorsomesal lobe of gonapophysis VIII broad (Figure 14)

*Paratanytarsus austriacus*
- If olive green ground colour and scutellum, antenna, fore tibia and maxillary palps with brown pigmentation; dorsomesal lobe of gonapophysis VIII narrow (Genus Micropsectra)
2020. Wing membrane with setae in apical 1/3 only, no setae in cell m; low tibial combs; completely dark brown (Figure 13)

*Micropsectra radialis*
- Wing membrane with rich setation, numerous setae in cell m; high tibial combs; dark brown or olive green ground colour
2121. Olive green ground colour, light scutellum, dorsocentrals including humerals 12–15 (Figure 12)

**Figure 11:**
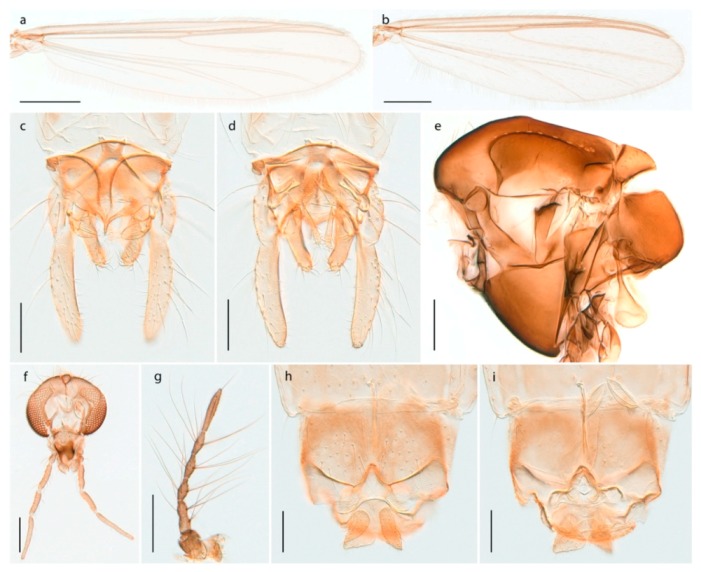
*Micropsectra insignilobus*. (**a**) Male wing, scale bar = 500 µm; (**b**) female wing, scale bar = 500 µm; (**c-d**) male hypopygium, scale bar = 100 µm; (**e**) male thorax, scale bar = 200 µm; (**f**) male head, scale bar = 200 µm; (**g**) female antenna, scale bar = 200 µm; (**h**–**i**) female genitalia, scale bar = 100 µm.

*Micropsectra logani*
- Dark brown species, brown scutellum, dorsocentrals including humerals 15–19 (Figure 11)

*Micropsectra insignilobus*
22. Mid and hind tibial combs with minute spur; anal point short and broad with high crests; superior volsella almost square; median volsella well developed, almost reaching tip of inferior volsella, with numerous simple lamellae (Figure 14)

*Paratanytarsus austriacus*
- Mid and hind tibial combs without spurs; if anal point broad, never with high crests; superior volsella roundish or fingertip-like in appearance; median volsella of variable length, always with cochleariform lamellae (genus Micropsectra)
2323. Wing membrane with setae in apical 1/4 only; superior volsella with serrate median margin; digitus hooked (Figure 13)

**Figure 12:**
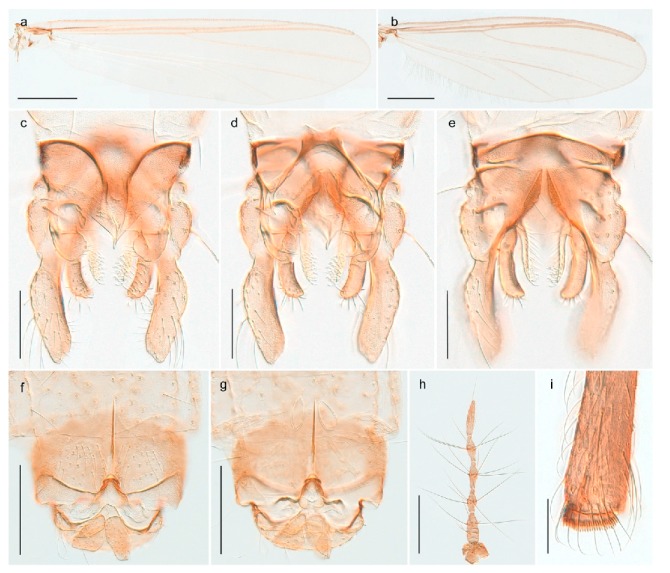
*Micropsectra logani*. (**a**) Male wing, scale bar = 500 µm; (**b**) female wing, scale bar = 500 µm; (**c**–**e**) male hypopygium, scale bar = 100 µm; (**f**–**g**) female genitalia, scale bar = 200 µm; (**h**) female antenna, scale bar = 200 µm; (**i**) tibial comb, scale bar = 50 µm.

*Micropsectra radialis*
- Wing membrane covered with setae; superior volsella with smooth median margin; digitus not hooked (Figures 11c and 12d)
2424. Dark olive ground colour; superior volsella almost circular (Figure 12)

*Micropsectra logani*
- Dark brown colour; superior volsella fingertip-like in appearance (Figure 11)

*Micropsectra insignilobus*
25. Wing membrane with macrotrichia in cells r4+5 and m1+2 (Figure 19)

*Sergentia coracina*
- Wing membrane without macrotrichia (e.g., Figure 20a)
2626. Wing with cubital fork proximal to crossvein RM; male antenna with 13 flagellomeres; male genitalia with mobile gonostylus (Figure 20)

*Stictochironomus psilopterus*
- Wing with cubital fork distal to crossvein RM (Figures 16a–18a); male antenna with 11 flagellomeres; male genitalia with rigid gonostylus (Figures 16c, 17c and 18b) (genus Chironomus)
2727. Antenna with 11 flagellomeres and well-developed plume; genitalia with well-developed gonocoxite and gonostylus (males)
28- Antenna with 5 flagellomeres and reduced plume; genitalia with reduced gonocoxite and cercus (females)
3028. Gonostylus constricted in apical 1/5; apical part of superior volsella parallel-sided, hooked; strong fore tarsal beard (Figure 16)

*Chironomus islandicus*
- Gonostylus constricted in apical ½ (Figures 17c and 18e); apical part of superior volsella enlarged, pediform; fore tarsal beard absent
2929. Posterior margin of abdominal tergites pale, giving the appearance of narrow, light transverse bands; legs dark brown (Figure 17)

*Chironomus lugubris*
- Abdomen and legs completely brownish black (Figure 18)
*Chironomus* sp. 1TE30. Body and legs completely brownish black
*Chironomus* sp. 1TE- At least fore femur and posterior margin of abdominal segments paler than rest
3131. Proximal half of femur yellowish-brown on all legs

*Chironomus lugubris*
- Proximal half of fore femur yellowish-brown, mid- and hind femur black

*Chironomus islandicus*
32. Wing veins R1 and R4+5 short, thick and fused with costa in thick clavus, ending at mid-point of wing (Figure 25a)
*Corynoneura* sp. 1ES- Wing veins R1 and R4+5 narrow, elongate, separated from costa until apex beyond mid-point of wing (e.g., Figure 53a,b)
3333. Macrotrichia present on wing membrane
34- Wing without macrotrichia on membrane
3634. Wing vein R4+5 always and costa usually ending proximal to vein M3+4 (Figure 53a,b); pseudospurs on tarsi absent

*Paraphaenocladius brevinervis*
- Wing vein R4+5 usually and costa always ending opposite or distal to vein M3+4 (e.g., Figure 32a); pseudospurs on tarsi present or absent
3535. Costa of wing without apical extension (rounded apex); pseudospurs on tarsi absent; clypeus large, bulbous (Figure 32b)

*Heterotrissocladius subpilosus*
- Costa of wing with apical extension; pseudospurs on tarsi present; clypeus normally developed
*Metriocnemus* spp. (see page 24)36. Squama of wing bare; eye hairy or pubescent; antenna with strong subapical seta (e.g., Figure 60e)
*Smittia* spp. (see page 33)- Squama usually with setae, if squama bare: eye bare and antenna without strong subapical seta
3737. Squama bare
38- Squama with setae
3938. Thorax with two characteristic acrostichals on mid-scutum (Figure 34e)
*Hydrosmittia oxoniana* and *Hydrosmittia* sp. 1ES- Thorax with 4–16 acrostichals on mid-scutum
*Allocladius* sp.1ES39. Setae present on preepisternum (Figures 35h and 38d) and eyes bare (microtrichia not present between ommatidia)
*Limnophyes* spp. (see page 23)- Seta usually absent on preepisternum; if present, eyes hairy (microtrichia extending beyond margin of ommatidia, e.g., Figure 26d)
4040. Clypeus enlarged, wider than diameter of pedicel in male

*Oliveridia tricornis*
- Clypeus normally developed, narrower than diameter of pedicel in male
4141. Eye hairy (microtrichia extending beyond margin of ommatidia)
*Cricotopus* spp. (see page 21)- Eye at most pubescent (microtrichia not extending beyond margin of ommatidia)
4242. Lateral spinules on spurs of mid- and hind tibiae diverge from shaft of spur (Figure 23i)
43- Lateral spinules on spurs of mid- and hind tibiae appressed to shaft of spur
4443. Male gonostylus broad, triangular, crista dorsalis weakly developed (Figure 23c); female antenna long, five elongate flagellomeres (Figure 23h)

*Chaetocladius holmgreni*
- Male gonostylus more or less parallel sided, crista dorsalis well developed (Figure 24f,g); female antenna short, six flagellomeres (Figure 24k), basal five almost spherical
*Chaetocladius incertus* and *C*. sp. 8ES44. Costa of wing clearly produced some distance beyond R4+5; wing membrane with coarse punctuation visible at 60× magnification (Figure 22a)
45- Costa of wing at most moderately produced; wing membrane with fine to moderate punctuation not visible at 60× magnification (e.g., Figure 61a)
4645. Acrostichals strong and decumbent, beginning close to antepronotum; eyes with broad with short dorsal extension; virga in males normally present
*Bryophaenocladius* sp. 5ES- Acrostichals scalpellate, present on mid-scutum; eyes without dorsal extension; virga in males absent

*Paralimnophyes*
46. Pulvilli large and distinct, mostly pad-like (Figure 55e)
*Psectrocladius* spp. (see page 30)- Pulvilli absent, vestigial or small, never more than ½ length of claw
4747. Small species, wing length about 1.5 mm; male with pin-like virga (Figure 71h)

*Tvetenia bavarica*
- Moderately large species, wing length usually more than 2.0 mm; virga, if present, not pin-like
4848. Acrostichals starting some distance from antepronotum; males with small, bare, pointed anal point (Figure 33c)

*Hydrobaenus conformis*
- Acrostichals, when present, starting near antepronotum; males with more robust, setose anal point (e.g., Figures 49d and 50e)
*Orthocladius* spp. (see page 26)

#### 3.1.2. Pupae

1. Anal lobe fringed with taeniate setae, but lacking distinctive macrosetae (e.g., Figure 10.77D in [73]); posterolateral corner of segment VI with sclerotized comb (e.g., figures 10.55E, F in [73],) (subfamily Chironominae)2- Anal lobe with or without setal fringe; if fringed, three distinctive macrosetae present on each side; posterolateral corner of segment VI never with comb72. Thoracic horn with multiple branches; tergites IV–V without median patch/patches of spines or spinules (tribe Chironomini) (e.g., figure 10.77D in [73])3- Thoracic horn not branched (e.g., figure 10.55C in [73],); tergites IV–V with median patch or paired patches of spines or spinules (tribe Tanytarsini) (e.g., figure 10.55E in [73])53. Tip of cephalic tubercle with circular field of spinules (e.g., figure 10.71A in [73])
*Sergentia*
- Tip of cephalic tubercle without spinules44. Anal lobe without dorsal seta; well-defined posterolateral comb with well-separated robust teeth present on segment VIII (e.g., figure 10.77E in [73])
*Stictochironomus*
- Anal lobe with dorsal seta; posterolateral spur or brush of closely adjacent spines present on segment VIII (e.g., figure 10.6E in [73])
*Chironomus*
5. Strong tubercle on pedicel sheath; tergites III–IV with spines in longitudinal, straight patches (figure 2b in [74])
*Tanytarsus*
- pedicel sheath without strong tubercle; if spines present in patches on tergites III-IV, patches not straight and longitudinal6 6. Wing sheath with pearl row; spine- and point patches absent from tergite III; tergite IV with one oval, centred point patch anteriorly (figure 10.55E in [73])
*Paratanytarsus*
- Wing sheath without pearl row; spines or spinules present in patches on tergite III; tergite IV with two point patches (figure 12e in [75], figure 14 in [76])
*Micropsectra*
7. Thoracic horn well developed, with horn sac and sometimes plastron plate (e.g., figure 4.5B in [77], figures 5.6A,B and 5.31D,E in [78])8- Thoracic horn present or absent, thin, without horn sac and plastron plate108. Two pairs of frontal setae; sheaths of fore- and midlegs straight, terminating beside recurved hindleg sheath at apex of wing sheath (subfamily Podonominae)
*Parochlus*
- One pair of frontal setae; all leg sheaths recurved beneath wing sheath (subfamily Tanypodinae)99. Thoracic horn tubular, without plastron plate; anal lobe triangular with pointed apex. *Arctopelopia*- Thoracic horn widest in middle, with plastron plate; anal lobe rounded with serrate border towards apical point
*Procladius*
10. Dorsomedian area of thorax with 3 setae, dc3 typically in supra-alar position, dc4 absent, or all dorsocentral setae absent. Fore- and midleg sheaths extend directly backward, hindleg sheath recurved beneath wing sheath (subfamily Diamesinae)11- Dorsomedian area of thorax with 4 setae, with neither dc3 nor dc4 in supra-alar position; all leg sheath recurved beneath wing sheath (subfamily Orthocladiinae)1211. Anal lobe with pointed apical projection (figure 7.7c in [79]); sternites without posterior thorn-like spines
*Pseudokiefferiella*
- Anal lobe without pointed apical projection (Figures 5c,d, 7e, 8c and 9g); sternites with posterior thorn-like spines (e.g., Figures 5d, 8l,m)
*Diamesa*
12. Anal lobe with a full or partial fringe of setae; fringe setae may be sparse or dense, short or long13- Anal lobe without a fringe of setae; anal lobes sometimes absent or greatly reduced 1813. Thoracic horn absent; lateral setae on tergite III taeniate
*Corynoneura*
- Thoracic horn present; lateral setae on tergite III not taeniate1414. Tergite IV with discrete spine patches or rows in the median field and/or along the posterior margin (e.g., figures 9.54c and 9.55h in [80])
*Psectrocladius*
- Tergite IV without discrete spine patches or rows, but shagreen present1515. Anal lobe with spinules at apex (figure 11C in [53])
*Oliveridia*
- Anal lobe without spinules at apex1617. Wing sheath with pearl row (figures 9.29C in [80])
*Heterotrissocladius*
- Wing sheath without pearl row
*Hydrobaenus*
18. Thoracic horn absent or minute tubercle19- Thoracic horn present, well developed2619. Tergites II-VIII with transverse row of closely set tubercles or spines along posterior margin (e.g., Figure 39g); anal macrosetae present (e.g., Figure 39j, figures 9.33C in [80])20- Tergites II-VIII without transverse row of closely set tubercles or spines along posterior margin; anal macrosetae absent2220. Armament along posterior margin of tergites II-VIII of blunt tubercles; anal macrosetae reduced (e.g., Figures 39g–j and 40f–i)
*Metriocnemus*
- Armament along posterior margin of tergites II-VIII of spines; anal macrosetae normally developed2121. Thoracic setae, particularly precorneals elongated (figure 9.46B in [80])
*Paralimnophyes*
- Thoracic setae normally developed
*Limnophyes*
22. Distinct bands of tiny spinules present on at least some conjunctives (figure 9.57D in [80])23- No distinct bands of tiny spinules present on any conjunctive or, if such bands are present, they appear as a continuation of the tergal shagreen2423. Tergites II–VII with similar-sized spinules covering most of tergites (figure 9.57D in [80]), frontal setae on prefrons
*Hydrosmittia*
- Tergites II–VII with anterior and posterior spinules clearly larger than median spinules, giving a transversely striped appearance; frontal setae on frontal apotome
*Allocladius*
24. Antepronotal seta 0–1 (figures 9.62B in [80])
*Smittia*
- Antepronotal seta 2–32525. Tergites III-VII with short, median posterior row of spinules (Figure 48l)*Orthocladius* (*Euorthocladius*)- Tergites more or less covered with fine shagreen, no rows or patches of spinules (figure 9.7G in [80])
*Bryophaenocladius*
26. Wing sheath with pearl row27- Wing sheath without pearl row2827. Thoracic horn with bulbous base and thin distal end; anal lobe with 3 macrosetae (Figures 71k–m)
*Tvetenia*
- Thoracic horn digitiform; anal lobe reduced with 0–2 macrosetae (figure 9.48 in [80])
*Paraphaenocladius*
28. Anal lobe with short, thorn-like, weakly bent, basally more or less swollen macrosetae (Figure 23j)
*Chaetocladius*
- Anal lobe with normally developed, apically hooked macrosetae2929. Tergites III-VII with central pair of circular spine patches (figure 9.41G in [80])*Orthocladius* (*Pogonocladius*)- Tergites III-VII without central pair of circular spine patches3030. Hook-row on tergite II absent (figure 9.40A in [80])*Orthocladius* (*Eudactylocladius*)- Hook-row on tergite II present3131. Hook-row on tergite II arranged in two even rows (e.g., figure 6.87 in [50])
*Cricotopus*
- Hook-row on tergite II arranged in three uneven rows (Figure 50d)*Orthocladius* (*Orthocladius*)

#### 3.1.3. Larvae

**Figure 13:**
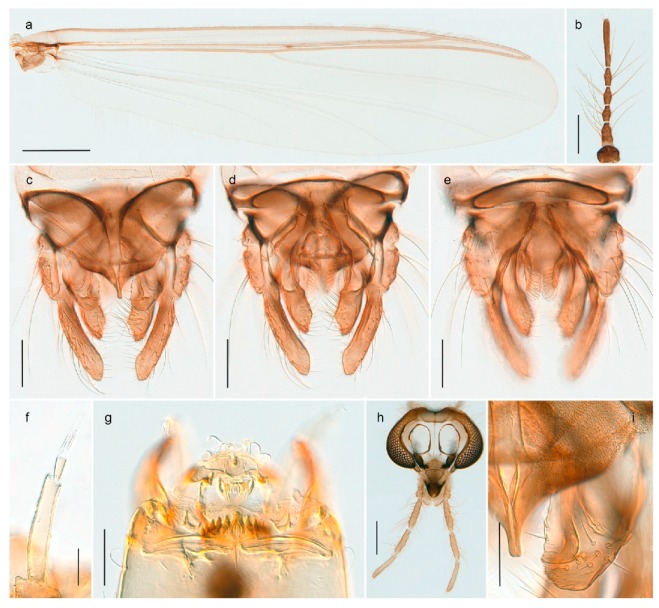
*Micropsectra radialis*. (**a**) Male wing, scale bar = 500 µm; (**b**) female antenna, scale bar = 200 µm; (**c-e**) male hypopygium, scale bar = 100 µm; (**f**) larval antenna, scale bar = 50 µm; (**g**) larval head ventral view, scale bar = 100 µm; (**h**) male head, scale bar = 200 µm; (**i**) male hypopygium, anal point and superior volsella, scale bar = 50 µm.

**Figure 14:**
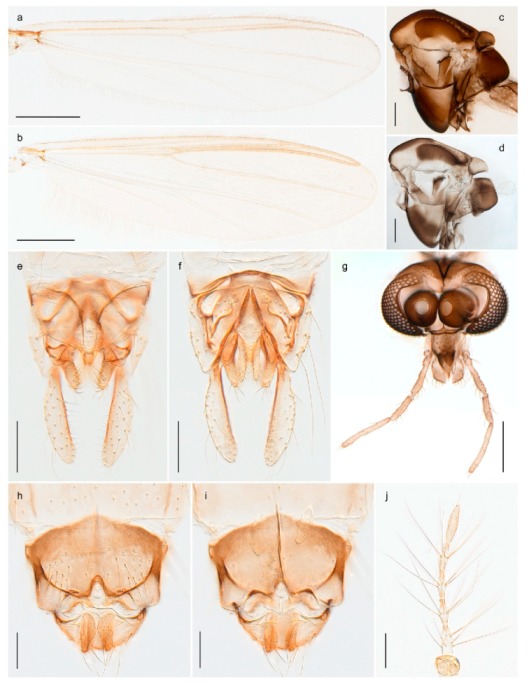
*Paratanytarsus austriacus*. (**a**) Male wing, scale bar = 500 µm; (**b**) female wing, scale bar = 500 µm; (**c**) male thorax, scale bar = 200 µm; (**d**) female thorax, scale bar = 200 µm; (**e**) male hypopygium dorsal view, scale bar = 100 µm; (**f**) male hypopygium ventral view, scale bar = 100 µm; (**g**) male head, scale bar = 200 µm; (**h**) female genitalia ventral view, scale bar = 100 µm; (**i**) female genitalia inner view, scale bar = 100 µm; (**j**) female antenna, scale bar = 100 µm.

**Figure 15:**
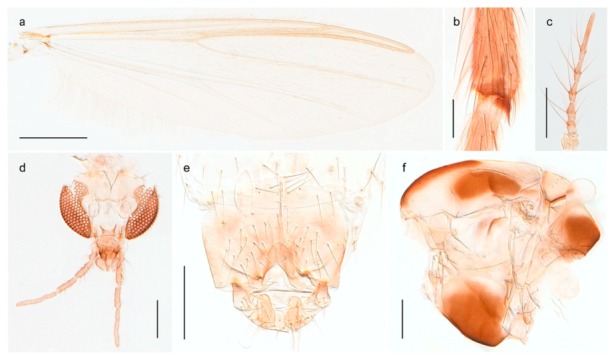
*Tanytarsus heliomesonyctios*. (**a**) Female wing, scale bar = 500 µm; (**b**) female tibial comb, scale bar = 50 µm; (**c**) female antenna, scale bar = 200 µm; (**d**) female head, scale bar = 200 µm; (**e**) female genitalia, scale bar = 200 µm; (**f**) female thorax, scale bar = 200 µm.

**Figure 16:**
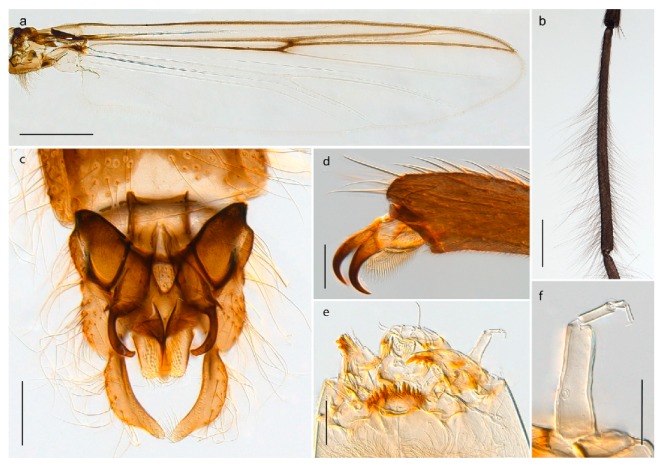
*Chironomus islandicus*. (**a**) Male wing, scale bar = 1000 µm; (**b**) male tarsus, scale bar = 500 µm; (**c**) male hypopygium, scale bar = 200 µm; (**d**) male tarsal claws, scale bar = 50 µm; (**e**) larval head ventral view, scale bar = 100 µm; (**f**) larval antenna, scale bar = 50 µm.

**Figure 17:**
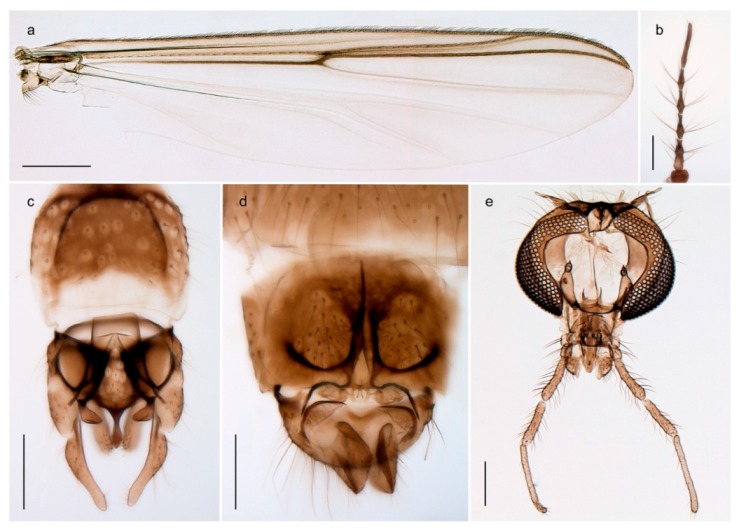
*Chironomus lugubris*. (**a**) Male wing, scale bar = 500 µm; (**b**) female antenna, scale bar = 200 µm; (**c**) male hypopygium, scale bar = 200 µm; (**d**) female genitalia, scale bar = 200 µm; (**e**) female head, scale bar = 200 µm.

**Figure 18:**
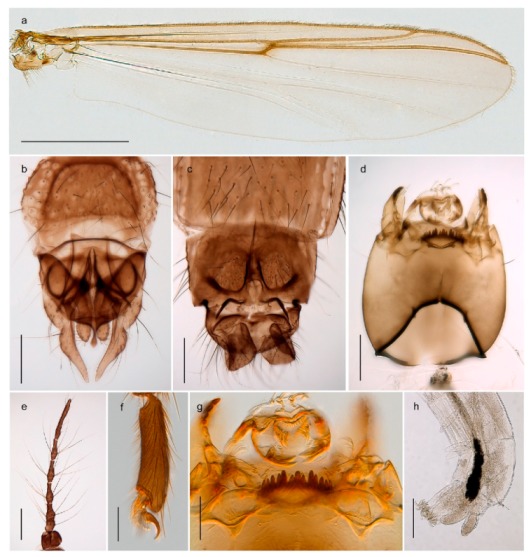
*Chironomus* sp. 1TE. (**a**) Male wing, scale bar = 500 µm; (**b**) male hypopygium, scale bar = 200 µm; (**c**) female genitalia, scale bar = 200 µm; (**d**) larval head ventral view, scale bar = 200 µm; (**e**) female antenna, scale bar = 200 µm; (**f**) male tarsal claws, scale bar = 50 µm; (**g**) larval mentum and labral surface, scale bar = 100 µm; (**h**) larva rear end, scale bar = 500 µm.

**Figure 19:**
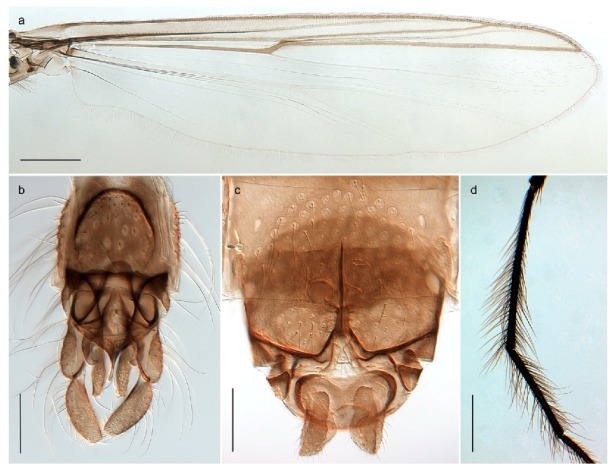
*Sergentia coracina*. (**a**) Male wing, scale bar = 500 µm; (**b**) male hypopygium, scale bar = 200 µm; (**c**) female genitalia, scale bar = 200 µm; (**d**) male fore tarsus with “beard”, scale bar = 200 µm.

**Figure 20:**
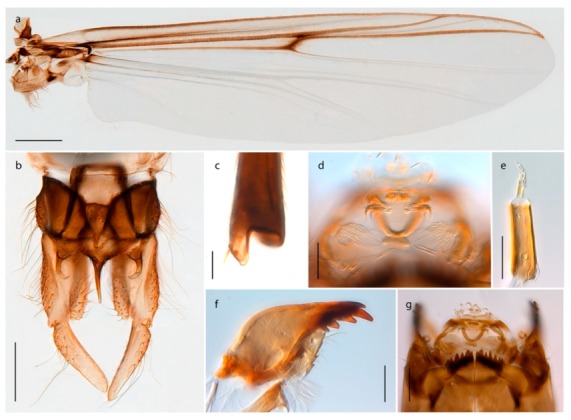
*Stictochironomus psilopterus*. (**a**) Male wing, scale bar = 500 µm; (**b**) male hypopygium, scale bar = 200 µm; (**c**) apex fore tibia, scale bar = 50 µm; (**d**) larval labral surface, scale bar = 50 µm; (**e**) larval antenna, scale bar = 50 µm; (**f**) larval mandible, scale bar = 50 µm; (**g**) larval head ventral view, scale bar = 100 µm.

**Figure 21:**
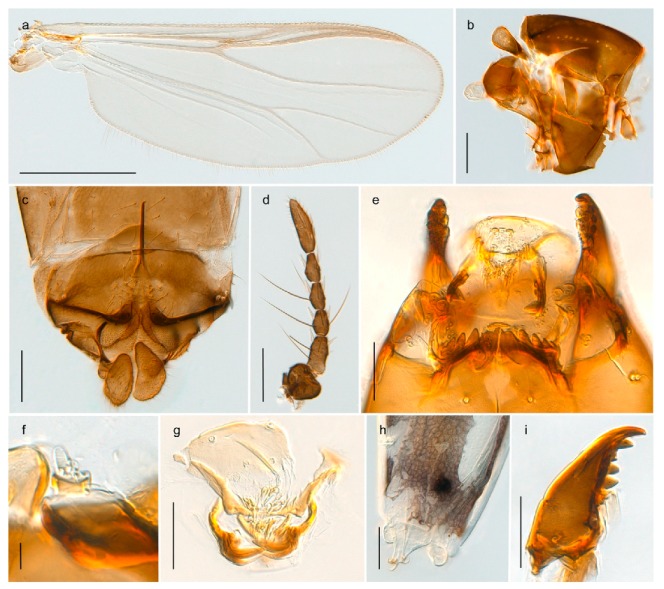
*Allocladius* sp. 1ES. (**a**) Female wing, scale bar = 500 µm; (**b**) female thorax, scale bar = 200 µm; (**c**) female genitalia, scale bar = 100 µm; (**d**) female antenna, scale bar = 100 µm; (**e**) larval head ventral view, scale bar = 50 µm; (**f**) larval antenna, scale bar = 10 µm; (**g**) larval premandibles, scale bar = 50 µm; (**h**) larva posterior end, scale bar = 100 µm; (**i**) larval mandible, scale bar = 50 µm.

**Figure 22:**
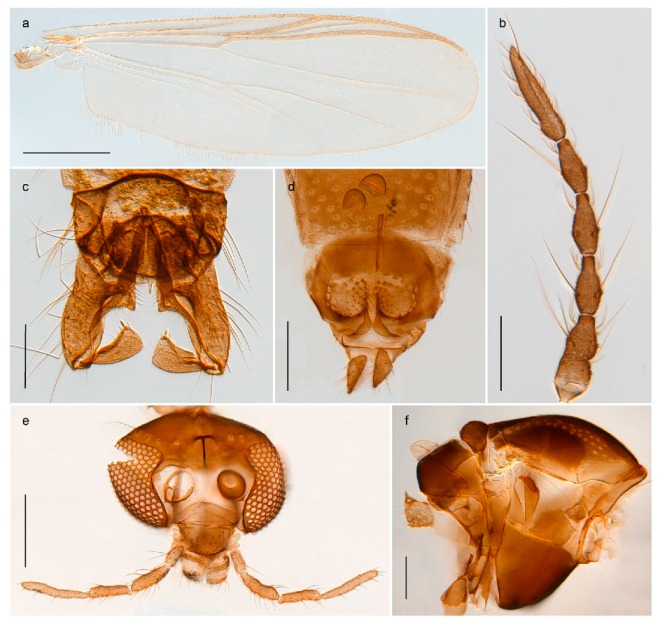
*Bryophaenocladius* sp. 5ES. (**a**) Female wing, scale bar = 500 µm; (**b**) Female antenna, scale bar = 100 µm; (**c**) male hypopygium, scale bar = 100 µm; (**d**) female genitalia, scale bar = 200 µm; (**e**) female head, scale bar = 200 µm; (**f**) female thorax, scale bar = 200 µm.

**Figure 23:**
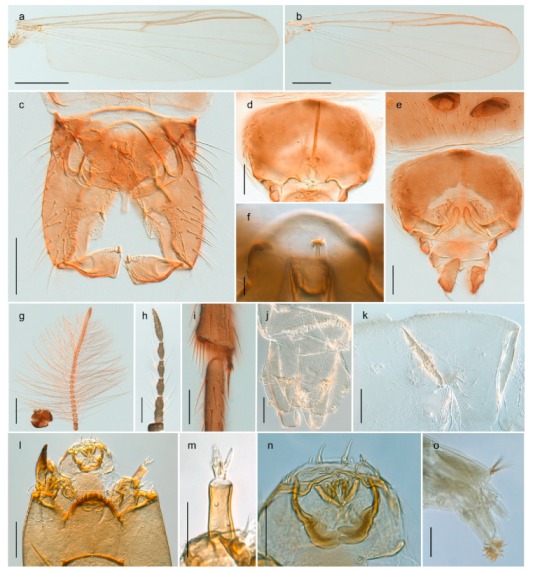
*Chaetocladius holmgreni*. (**a**) Male wing, scale bar = 500 µm; (**b**) Female wing, scale bar = 500 µm; (**c**) male hypopygium, scale bar = 100 µm; (**d**) female genitalia inner structures, scale bar = 100 µm; (**e**) female genitalia ventral view, scale bar = 100 µm; (**f**) male genitalia virga, scale bar = 15 µm; (**g**) male antenna, scale bar = 200 µm; (**h**) female antenna, scale bar = 100 µm; (**i**) hind tibial comb and spur, scale bar = 50 µm; (**j**) pupal posterior end, scale bar = 100 µm; (**k**) pupal thoracic horn, scale bar = 100 µm; (**l**) larval head ventral view, scale bar = 100 µm; (**m**) larval antenna, scale bar = 50 µm; (**n**) larval premandibles, scale bar = 50 µm; (**o**) larva posterior end, scale bar = 200 µm.

**Figure 24:**
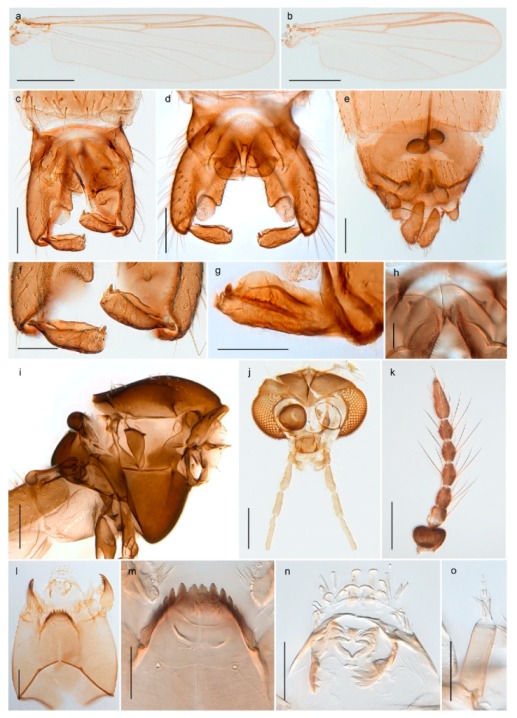
*Chaetocladius incertus* and *Chaetocladius* sp. 8ES. (**a**) *C. incertus* male wing, scale bar = 500 µm; (**b**) *C. incertus* female wing, scale bar = 500 µm; (**c**) male hypopygium, scale bar = 100 µm; (**d**) *C. incertus* male hypopygium, scale bar = 100 µm; (**e**) *C. incertus* female genitalia, scale bar = 100 µm; (**f**) *C*. sp. 8ES male gonostyli, scale bar = 50 µm; (**g**) *C. incertus* male gonostyli, scale bar = 50 µm; (**h**) male hypopygium, virga, scale bar = 40 µm; (**i**) *C.* sp. 8ES thorax, scale bar = 200 µm; (**j**) *C. incertus* male head, scale bar = 200 µm; (**k**) *C. incertus* female antenna, scale bar = 100 µm; (**l**) *C. incertus* larval head ventral view, scale bar = 100 µm; (**m**) *C. incertus* larval mentum, scale bar = 50 µm; (**n**) *C. incertus* larval premandibles, scale bar = 50 µm; (**o**) *C. incertus* larval antenna, scale bar = 50 µm.

**Figure 25:**
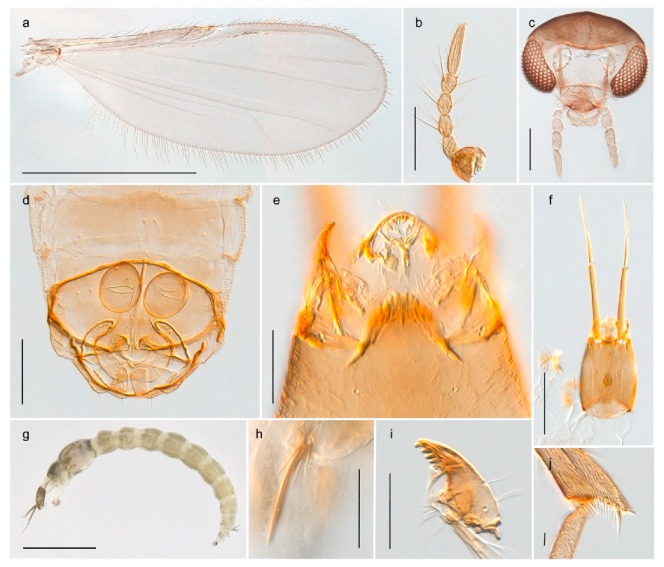
*Corynoneura* sp. 1ES. (**a**) Female wing, scale bar = 500 µm; (**b**) female antenna, scale bar = 100 µm; (**c**) female head, scale bar = 100 µm; (**d**) female genitalia, scale bar = 100 µm; (**e**) larval head ventral view, scale bar = 50 µm; (**f**) larval head dorsal view, scale bar = 100 µm; (**g**) larva lateral view, scale bar = 1 mm; (**h**) spine on larval posterior parapod, scale bar = 50 µm; (**i**) larval mandible, scale bar = 50 µm; (**j**) tibial comb of hind leg, scale bar = 10 µm.

**Figure 26:**
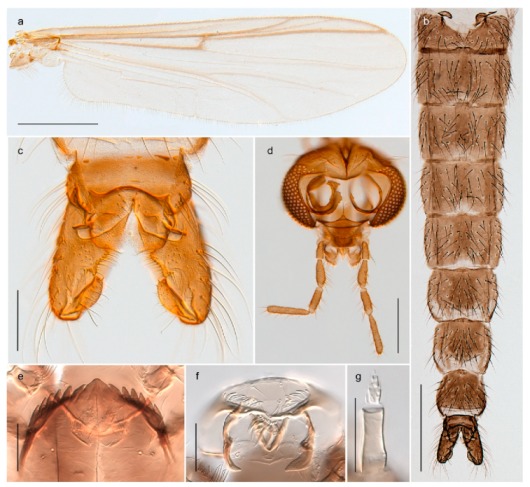
*Cricotopus* (*Cricotopus*) *gelidus*. (**a**) Male wing, scale bar = 500 µm; (**b**) male abdomen, scale bar = 500 µm; (**c**) male hypopygium, scale bar = 100 µm; (**d**) male head, scale bar = 200 µm; (**e**) larval mentum, scale bar = 50 µm; (**f**) larval labral surface, scale bar = 50 µm; (**g**) larval antenna, scale bar = 50 µm.

1. Antenna retractile into head capsule; prementum with distinctly developed ligula (Figure 3g) (subfamily Tanypodinae)2- Antenna not retractile into head capsule; prementum not with distinctly developed ligula (e.g., Figures 20g and 71n)32. Head elongate; dorsomentum without well developed teeth (Figure 3g); body without lateral fringe of setae
*Arctopelopia*
- Head rounded to oval; dorsomentum with well developed teeth (Figure 4j); body with well-developed fringe of lateral seta
*Procladius*
3. Premandible absent; procercus 8–10 times longer than wide (figure 4.4E in [81]) (subfamily Podonominae)
*Parochlus kiefferi*
- Premandible present (e.g., Figure 8h); procercus rarely more than 4 times longer than wide44. Antennal segment 3 annulated (e.g., Figures 8g and 9h); prementum with three strong bushes (subfamily Diamesinae)5- Antennal segment 3 not annulated; prementum at most with a single bush65. Procercus well developed, longer than wide (figure 7.14F in [82]); body with dark setae
*Pseudokiefferiella*
- Procercus absent or very small (Figure 8k); body with pale setae
*Diamesa*
6. Mentum with well-developed, striated ventromental plates (e.g., Figures 13g, 16e and 18g) (subfamily Chironominae)7- Mentum without or only weakly developed ventromental plates, never striated (e.g., Figures 21e and 23l) (subfamily Orthocladiinae)127. Antenna on pedestal (e.g., Figure 13f, figures 22 and 26 in [10]); ventromental plates much wider than long, almost meeting medially (e.g., Figure 13g) (tribe Tanytarsini)8- Antenna not on pedestal; ventromental plates not much wider than long, well-separated medially (e.g., Figures 18g and 20g) (tribe Chironomini)108. Premandible with 3–4 main teeth (figures 28 and 31 in [10])
*Tanytarsus*
- Premandible with 2 main teeth (e.g., figure 23 in [10])99. Lauterborn organs on long pedicels, extending well beyond apex of antenna (Figure 13f, figure 18 in [10],); pecten epipharyngis consisting of three separate, serrated scales (Figure 13g, figures 19 and 23 in [10])
*Micropsectra*
- Lauterborn organs on short pedicels, not reaching apex of antenna (figure 10.80D in [83]); pecten epipharyngis consisting of 3–5 rounded or pointed scales (figure 10.80G in [83])
*Paratanytarsus*
10. Ventral side of mandible with basal row of radially arranged furrows (figure 10.7C in [83]); body with (e.g., Figure 18h) or without ventral tubuli
*Chironomus*
- Ventral side of mandible without basal row of radially arranged furrows; body without ventral tubuli1111. Mandible with 4 inner teeth (figure 10.59A in [83]); small Lauterborn organs opposite on antennal segment 2 (figure 10.59B in [83])
*Sergentia*
- Mandible with 2–3 inner teeth (Figure 20f); small Lauterborn organs alternate on antennal segments 2 and 3 (Figure 20e)
*Stictochironomus*
12. Anal end without procercus, mostly terrestrial and semi-terrestrial species (e.g., Figure 21h)13- Anal end with procercus (e.g., Figure 23o)1613. Preanal and anal segments and posterior parapods bent at right angles to axis of rest of body (figure 9.9G in [84],)
*Bryophaenocladius*
- Preanal and anal segments in same axis as rest of body (Figure 21h)1414. Antenna not strongly reduced; antennal blade shorter than antenna (e.g., Figures 59i, 60j and 61g)
*Smittia*
- Antenna strongly reduced; antennal blade slightly longer than antenna (Figure 21f)1515. Mentum with 5 lateral teeth (Figure 21e); posterior parapod with 6–7 claws (Figure 21h)
*Allocladius*
- Mentum with 4 lateral teeth (figures 9.39A in [84]); posterior parapod with more than 7 claws
*Hydrosmittia*
16. Antenna longer than head (Figure 25f)
*Corynoneura*
- Antenna shorter than head (e.g., Figure 23l)1717. Antenna with 7 segments, third segment much smaller than fourth, seventh segment hair-like (figures 9.37D,E in [84])
*Heterotrissocladius*
- Antenna with fewer segments, last segment can be hair-like (e.g., Figure 24m)1818. Labral seta SI bifid and labral lamella absent (e.g., Figure 26f); setal tufts on at least the first 6 abdominal segments (figure 23.2 in [50])
*Cricotopus*
- Labral seta SI usually coarsely or finely plumose, simple, serrate or palmate and labral lamella present; if SI bifid and labral lamella absent, setal tufts absent from first 6 abdominal segments1919. Labral seta SI bifid (e.g., Figure 51k)
*Orthocladius*
- Labral seta SI plumose, serrate, simple or palmate (e.g., Figures 23n, 54g, 58j and 71j)2020. Labral seta SI distinctively palmate with 3–10 lobes; premandible with one apical tooth (e.g., Figures 54i and 58j)
*Psectrocladius*
- Labral seta SI simple, serrate or plumose; premandible with one or more teeth (e.g., Figures 23n, 24l and 71j)2121. Antenna with 6 segments, consecutively smaller, sixth segment vestigial (e.g., Figure 33j)22- Antenna with 4–5 segments, sometimes not consecutively smaller (e.g., Figures 23m, 36k, 37i and 71i)2322. Mentum with single, weakly sclerotized median tooth (figure 9.51A in [84]); ventromental plates narrow and acute at apices; antennal segment 1 more than 2.5x longer than segment 2 (figure 9.51B in [84])
*Oliveridia*
- Mentum with double, strongly sclerotized median tooth (Figure 33i); ventromental plates broader and rounded apically; antennal segment 1 less than 2.0x longer than segment 2 (Figure 33j)
*Hydrobaenus*
23. Mandible with 3 inner teeth (Figures 36j, 37l and 71o)24- Mandible with at least 4 inner teeth (Figures 24j, 42k, 43j and 44b)2524. Premandible with one tooth (Figure 71j); body with long, strong setae, at least ½ length of segment
*Tvetenia*
- Premandible with 2 apical and 2 more or less distinct inner teeth (Figure 37j)*Limnophyes* *25. Procercus and anal setae posteriorly directed (figure 9.55E in [84])*Paraphaenocladius* **- Procercus and anal setae not posteriorly directed (Figures 23o and 42l)2626. Premandible with serrated outer tooth (Figure 24n)
*Chaetocladius incertus*
- Premandible without serrated outer tooth2727. Mentum with double or single median tooth deeply set (e.g., Figures 40n and 43k)
*Metriocnemus*
- Mentum with median tooth higher than first lateral tooth (Figure 23l)2828. Antennal segment 3 and 4 subequal (Figure 23m); premandibular brush absent
*Chaetocladius holmgreni*
- Antennal segment 3 shorter than 4th segment (figure 9.59B in [84]); premandibular brush present*Paralimnophyes* **** Larvae of *Limnophyes brachytomus* and *Limnophyes schnelli* are unknown. ** Larva of *Paraphaenocladius brevinervis* is unknown. *** Generic diagnosis of *Paralimnophyes* larva is based on one species only.

## 4. Discussion

Many of the chironomids encountered on Svalbard are difficult to identify, either due to subtle morphological differences or to the lack of taxonomic revisions. Often, the original literature and vouchered reference material must be consulted, and even then, the results can be ambiguous. In this section, we comment on various observations made and present arguments for the identification (or previous misidentification) of genera and species reported from Svalbard and Jan Mayen. When interesting, we also refer to the known geographical distribution and genetic similarity with DNA barcodes from other populations represented in BOLD.

### 4.1. Podonominae

#### 4.1.1. Parochlus

*Parochlus kiefferi* and *Paralimnophyes* sp. were reported from birds’ nests on Spitsbergen as “old and damaged” larval head capsules [85]. Apart from these findings, the two genera have never been recorded from Svalbard. We have examined the head capsule remains that were reported by Pilskog et al. [85]. They are in a relatively poor condition but, based on the mentum and very short antennal segment 1, the three specimens identified as *Parochlus kiefferi* likely belong to *Smittia* instead. However, we do include *Parochlus kiefferi* in the key since it is not unlikely that it will be found on Svalbard in the future (we have seen records from the Norwegian mainland, Iceland and Greenland).

### 4.2. Tanypodinae

#### 4.2.1. Arctopelopia

The species *Arctopelopia barbitarsis* was recorded in stomach content of Arctic char from lakes on Bear Island by Berg, Finstad, Olsen, Arnekleiv and Nilssen [19] (identified by T. Ekrem). Re-examination of the specimens have revealed that these belong to *A. melanosoma* (Goetghebuer, 1933). Comparison of DNA barcode data in BOLD shows that the BIN with *A. melanosoma* (BOLD:AAD2100) containing members from Bear Island, Greenland and Canada is genetically distinct from the group with *A. barbitarsis* with barcodes from continental Norway and Finland. We have examined two females from Bear Island identified as *A. barbitarsis* by Edwards [31] and find these conspecific with examined females of *A. melanosoma.* We thus regard *A. barbitarsis* as absent from the Svalbard Archipelago.

#### 4.2.2. Procladius

*Tanypus frigidus* Holmgren, 1869 was originally described from Bear Island (Mount Misery) [26]: p. 48. The name is listed as a junior synonym of *Procladius* (*Holotanypus*) *crassinervis* in the World Catalogue of Chironomidae [2] which other authors have been treated as a subjective synonym of *Procladius* (*Holotanypus*) *culiciformis* (Linnaeus, 1767) [57,86]. DNA barcodes of our specimens from several localities on Bear Island and Spitsbergen cluster nicely with those of specimens from northern Norway but are more than 7% different from specimens in BOLD identified as *P*. (*H*.) *culiciformis*. Moreover, the original description of *Tipula culciformis* indicate that the specimens Linnaeus described had pale legs [87]: p. 978, while our specimens are completely dark. *Tanypus crassinervis* was originally described with bare wings [66]: p. 817, which differs from later interpretations of this species, e.g., [47]. Our specimens from Bear Island have moderately hairy wings in the adult males, while female wings have more hairs (Figure 4a,b) and therefore, do not fit the original description. Comparison of DNA barcode data in BOLD, show that there currently are five BINs with the name of *P*. (*H*.) *crassinervis*. Sequences from our Svalbard specimens populate BOLD:AAB9256 together with specimens from northern Canada, Greenland, continental Norway and Finland. The genus and the species group are in need of revision [57], but we choose to keep the name *P*. (*H*.) *frigidus* here since our specimens were collected close to the type locality, and because they clearly best fit the original description under this name. We thus regard previous records of *P*. (*H*.) *crassinervis* from Svalbard to be misidentifications and/or caused by a doubtful synonymy and reinstate the name *Procladius* (*H*.) *frigidus* for specimens associated with the Svalbard population.

*Procladius* cf. *choreus* was reported from Londonelva, New Ålesund on Spitsbergen by Lods-Crozet, et al. [88]. As this constitutes an uncertain identification in a genus in need of revision, we currently do not treat *P. choreus* as present on Svalbard.

### 4.3. Diamesinae

#### 4.3.1. Diamesa

*Diamesa hyperborea* Holmgren, 1869 was originally described from Bear Island [26], and has been documented and DNA barcoded by us. Pedersen [70] and Sæther [89] indicated that *Diamesa hyperborea* (as *D. ursus* Kieffer, 1919) is present on Spitzbergen. Their distributional records likely originate from Styczyński and Rakusa-Suszczewski [90] who collected larva in a pond near Hornsund. We have been unable to confirm this by examination of specimens and regard the presence on Spitzbergen as questionable since the only known record is based on larvae only.

*Diamesa incallida* was reported as pupae from Bayelva near Ny Ålesund, Spitsbergen [88]. We have been unsuccessful in locating the vouchers of these records (pers. comm. with Brigitte Lods-Crozet and Valeria Lencioni). Thus, we consider the identification of the single finding of *D. incallida* as doubtful and regard this species as absent from Svalbard.

*Diamesa lindrothi* (or “*D*. cf. *lindrothi*”) apparently has been reported from Svalbard as larva only [20,90,91]. We have not seen material of *D. lindrothi* from Svalbard or Jan Mayen, and regard the species records based on larva only as doubtful as this species has a morphology very similar to that of *Diamesa bertrami* and descriptions of *D. lindrothi* larvae from Svalbard [90] fit well with observations we have made of *D. bertrami*. We therefore regard *D. lindrothi* as not present on Svalbard until its occurrence there is proven.

*Diamesa lundstromi* Kieffer, 1918, was recently reported as present on Svalbard [92,93]. The species’ name originates from Kieffer [94] as a new name for specimens from Bear Island and Spitsbergen previously assigned to *Diamesa arctica* (Boheman, 1865) in Kieffer and Lundbeck [71]. *Diamesa lundstromi* is currently considered as a nomen dubium [2]. We have not seen material that could help clarify this species name, nor been able to locate the type material in Zoologisches Forschungsmuseum Alexander Koenig in Bonn, Germany.

#### 4.3.2. Pseudokiefferiella

The genus *Pseudokiefferiella* has been treated as monotypical, the only valid species being *P. parva* (Edwards, 1932) originally described from Scotland. The species was recorded from Spitsbergen as larvae [91]. However, we collected one female from Spitsbergen that is morphologically and genetically different from continental *P. parva*; its DNA barcode clusters with those of numerous females from Greenland and more distantly (3.9% divergent) with a male from Finnmark. We believe the larvae collected by Losos and Kubíček (1988) belong to this species, and that it is likely new to science. Material from Greenland and northern North America should be considered before description as there are indications of additional taxa that should be treated simultaneously [15]: p. 617).

### 4.4. Chironominae, Tanytarsini

#### Tanytarsus

*Tanytarsus heliomesonyctios* Langton, 1999 was originally described from Ellesmere Island in Arctic Canada [74]. Stur and Ekrem [10] recorded the species from Spitsbergen and described the larva based on associations through DNA barcodes. Although all specimens collected in the high Arctic so far have been females and support the assumption that *T. heliomesonyctios* is a parthenogenetic species, we have a DNA barcodes from a male collected in northern Norway (Porsanger, Finnmark) that clusters with females from the Arctic as well as with specimens throughout Canada (BIN BOLD:AAC2863). Adult males were recently described from northeast Russia [95]. We suspect that the species is facultatively parthenogenetic with males appearing at lower latitudes.

### 4.5. Chironominae, Chironomini

#### 4.5.1. Chironomus

The species *Chironomus hyperboreus* originally described from Greenland [96,97] has been reported from Spitsbergen and Bear Island [26,31,98]. Some authors considered the name as a senior synonym of *Chironomus islandicus* [99], but Pedersen [65] provided convincing evidence for separate species. We have DNA barcodes of *C. hyperboreus* specimens from continental Norway that match with populations from Greenland and Canada in BOLD, and DNA barcodes of populations from Bear Island and continental Norway that match a population from Iceland identified as *C. islandicus*. The COI-sequences of the two groups differ by approximately 5% K2P-distance. Moreover, our specimens from Bear Island agree morphologically with the diagnostic characteristics of *C. islandicus* discussed by Pedersen [65]. Although we cannot be sure about the identity of previous records of *C. hyperboreus* from Svalbard, we think there is reason to believe that these were based on misidentifications of *C. islandicus*, since the two species are morphologically very similar and the distinction between them was first properly presented by Pedersen [65]. *Chironomus islandicus* was previously known from Iceland, Greenland and Finland [65,86]. The larvae of both *C. islandicus* and *C. hyperboreus* are of *salinarius*-type, i.e., lack the ventral and lateral tubuli seen in many *Chironomus* species. Rempel’s [100] description of *C. hyperboreus* from Saskatchewan was based on misidentification of a species later named *C. rempelii* [101].

*Chironomus* sp. 1TE may be an undescribed species close to *C. saxatilis* Wülker et al., 1981. The polytene chromosomes of a specimen with COI-barcode grouping with *C.* sp. 1TE in BIN BOLD:AAC0592 indicate that it cannot be *C. saxatilis* and do not match any cytologically studied species from the Holarctic (Jon Martin pers comm.). The species has *halophilus*-type larvae.

#### 4.5.2. Sergentia

The species *Sergentia coracina* is listed as present on Svalbard in recent checklists [86,92,98]. The record seems to have originated from Edwards’ [28,29] records from Bear Island, referring to “*Lauterbornia* ? *coracina*, Zett.” and “*Chironomus coracinus*, Zett.” respectively. Later sources report the species from Spitsbergen [47,101], but this was likely based on a misconception that Svalbard and Spitsbergen refer to the same land masses [29]. Edwards [31] further discussed the Bear Island records and described the previously recorded specimens as different from Zetterstedt’s types of *Chironomus coracinus*. He named the species *Chironomus psilopterus* (see comments on *Stictochironomus*). We have not seen material of *Sergentia coracina* from Svalbard and do not know of reliable records. Based on the above discussion, we therefore regard the species to be absent from the archipelago but include *Sergentia* in the identification keys since it is not completely unlikely that it will be found there in the future.

#### 4.5.3. Stictochironomus

*Stictochironomus psilopterus* (Edwards, 1935) was described as *Chironomus psilopterus* based on material from several lakes on Bear Island [31]. The species was later placed in *Sergentia* and also recorded from Lapland [47,101,102] but in recent checklists, the name has been regarded as a nomen dubium in *Sergentia* [103]. According to the original description, however, the species belongs to the genus *Stictochironomus* and we are confident that we collected this exact species as males and larvae on Bear Island (Figure 20). The species appears similar to *S. sticticus* (Fabricius, 1781) and *S. unguiculatus* (Malloch, 1934) in the adult male, but can be separated by more than 10% divergence in DNA barcodes. We DNA-barcoded several additional, likely undescribed species of *Stictochironomus* from other regions, and a taxonomic revision of the genus is needed to identify morphologically diagnostic features of all species. Bista et al. [104] recorded and DNA barcoded specimens identified as “*Sergentia psiloptera*” from the UK (GenBank accessions KY225371, KY225372), but this appears to be an erroneous identification that matches our *Sergentia* sp. TE2 from mainland Norway.

### 4.6. Orthocladiinae

#### 4.6.1. Allocladius

The species we call *Allocladius* sp. 1ES seems to be close to *Allocladius nanseni* (Kieffer, 1926), but is separated from the latter by >6% uncorrected genetic distance. Morphologically, it is difficult to separate the species from *A. nanseni*, *A. aizaiensis* Wang, 1990 and *A. arenarius* (Strenzke, 1960) as described by Ferrington and Sæther [48]. We collected one female and several larvae of this species, which is the first record of *Allocladius* from Svalbard. DNA barcodes in BOLD match those of specimens from Arctic Canada and Greenland.

#### 4.6.2. Bryophaenocladius

We collected and barcoded females of one *Bryophaenocladius* species from Spitsbergen but are unable to associate them with a known species. Our species is therefore assigned the interim name *Bryophaenocladius* sp. 5ES. The DNA barcode match that of a specimen collected on Iceland, but otherwise there are no matching records in BOLD at present.

We also examined a male *Bryophaenocladius* from Spitsbergen collected by Brigitte Lods-Crozet near Ny Ålesund. It is not possible for us to associate this male with the above-mentioned female, nor to any described species. It is rather similar to *Bryophaenocladius saanae* Tuiskunen, 1986, but has a considerably higher antennal ratio (AR 1.75 vs. AR 1.25 in *B. saanae*).

#### 4.6.3. Camptocladius

Recent listings of the species *Camptocladius stercorarius* from Spitsbergen [3,86] seem to originate from Holmgren’s [26] report of material from “Green Harbour”, “Advent Bay”, “Nordkap” and “Storfjorden” under the junior synonym of *Chironomus byssinus* (Schrank, 1803). We examined males and females from Holmgren’s Spitsbergen material of *C. byssinus* deposited in the Swedish Museum of Natural History in Stockholm. Unfortunately, the seven specimens were considerably damaged in the mail, to the extent that some broken off parts were impossible to assign to any labelled individual. However, it is clear that at least the two examined male specimens do not belong to *Camptocladius stercorarius* but to *Smittia extrema* Holmgren, 1869. Thus, we regard *C. stercorarius* to be absent from Svalbard, agreeing with the conclusion reached by Edwards [29].

#### 4.6.4. Chaetocladius

*Chaetocladius perennis* has been reported from Svalbard by several authors, e.g., [92]. However, the DNA barcodes of *Chaetocladius* specimens morphologically fitting previous descriptions of *C. perennis* from Svalbard are very divergent from the barcodes in continental populations of this species. Closer examination of our Svalbard specimens reveals that these have dark brown halteres (in macerated individuals) as opposed to the pale or yellowish halteres described by Meigen [105] and later by Edwards [106] based on specimens from Germany and Great Britain, respectively. Unpublished notes by Edwards confirm that he had examined presumed type specimens of Meigen before writing his key to British Chironomidae (M. Spies pers. comm. 05.ix.2016). BOLD holds DNA barcode data of specimens from continental Norway, Greenland and Canada that belong to a single BIN (BOLD:AAC8747). We have seen specimens from Central Norway belonging to the «true» *C. perennis* cluster that fit the original description, and there are DNA barcodes of specimens from Germany and southern Canada in the same BIN (BOLD:ACF6903). We have not seen specimens from Svalbard or other Arctic regions that fit Meigen’s (1830) or Edwards’ (1929) description *of C. perennis*. Moreover, the larvae associated with the Svalbard population through DNA barcodes differ markedly from described *Chaetocladius* larvae in having a premandible with three strong teeth of which the apical one is serrated (Figure 24n); see the genus diagnosis in Andersen, et al. [84]). Sæther [57] examined two syntypes of *Camptocladius incertus* Lundström, 1915 from Siberia and synonymized this name with *Chaetocladius perennis* (Meigen, 1830) mainly based on male hypopygial features. Sæther did not describe the halteres of the examined types, but the original description states that the species has black halteres [107]. Thus, we regard the Svalbard population to be *Chaetocladius incertus* (Lundström, 1915) and Sæther’s synonymy as incorrect.

A third species of *Chaetocladius* present on Svalbard is morphologically very similar to *C. incertus*, but separated from the latter by >7% uncorrected genetic distance. We only examined one specimen from Spitsbergen (male, specimen ID SV91), but it seems to be slightly different from *C. incertus* in having an evenly broad (parallel-sided) gonostylus (Figure 24f). More specimens are needed to describe this possibly new species properly. Thus, a temporary name *Chaetocladius* sp. 8ES is assigned to this specimen in BOLD. We have also seen two *Chaetocladius* specimens from Jan Mayen that are similar to *Chaetocladius* sp. 8ES, but due to the condition of these slide mounted specimens we cannot evaluate wether or not they are conspecific. They have no associated DNA barcodes.

*Chaetocladius dentiforceps*, *C. dissipatus*, *C. laminatus* and *C. piger* (Goetghebuer, 1913) have been reported from Svalbard in ecological studies [88,108], but in low numbers. *Chaetocladius dentiforceps*, *C. laminatus* and *C. piger* were only recorded as immatures and these records must be regarded as doubtful. Moreover, the re-examination of a pupa from this material previously determinated as *C. laminatus* showed that it likely belongs to *C. holmgreni* (Jacobson, 1898) instead. Re-examination of adult males from Lods-Crozet’s material determined as *Chaetocladius dissipatus* and *C. suecicus* revealed that these are morphologically consistent with what is currently regarded as *C. holmgreni* and *C. incertus* respectively. In summary, we consider three *Chaetocladius* species as recorded from Svalbard with certenty, *C*. *holmgreni*, *C. incertus* and *C.* sp. 8ES.

#### 4.6.5. Corynoneura

The genus *Corynoneura* is represented on Svalbard by one form that might be a parthenogenetic population of *Corynoneura arctica* Kieffer, 1923. We are, however, not able to assign the examined females to *C. arctica* based on morphology, and DNA barcodes from the Svalbard population in BOLD belonging to a BIN (BOLD:ABZ8189) separated from its nearest neighbour (containing specimens identified as *C. arctica*) by at least 1.58% uncorrected genetic distance. The BIN containing the Svalbard specimens also includes representatives from throughout northern Canada, one from Central Norway, and one from Alaska. Our record of *Corynoneura* sp. 1ES is the first contemporary record of the genus from Svalbard, but subfossil material identified as the *Corynoneura arctica* type has been recorded from Spitsbergen (referred to as *C. scutellate* type) [109].

#### 4.6.6. Cricotopus

*Cricotopus* is one of the most widely distributed and species rich genera in the subfamily Orthocladiinae. It appears to be particularly diverse in the Holarctic region and has numerous species in the Arctic. Six species are recorded from Svalbard with certainty (see key below). In addition, *Cricotopus* (*Cricotopus*) *polaris* was reported by Lods-Crozet, et al. [88] and Marziali, et al. [108]. Based on examination of material kindly sent by B. Lods-Crozet, the specimens belong more likely to *Cricotopus* (*C.*) *tibialis* (Meigen, 1804). *Cricotopus* (*Cricotopus*) *humeralis* with its junior synonym *Cricotopus* (*Cricotopus*) *ephippium* (Zetterstedt, 1838) was recorded from northern Spitsbergen by Edwards [29], as a *C. humeralis*. However, according to Hirvenoja [50], page140, Edwards misinterpreted the species *C. tibialis*. We have not seen material of *C.* (*C*.) *humeralis* (or *ephippium*) from Svalbard.

##### Key to Species

**Figure 27:**
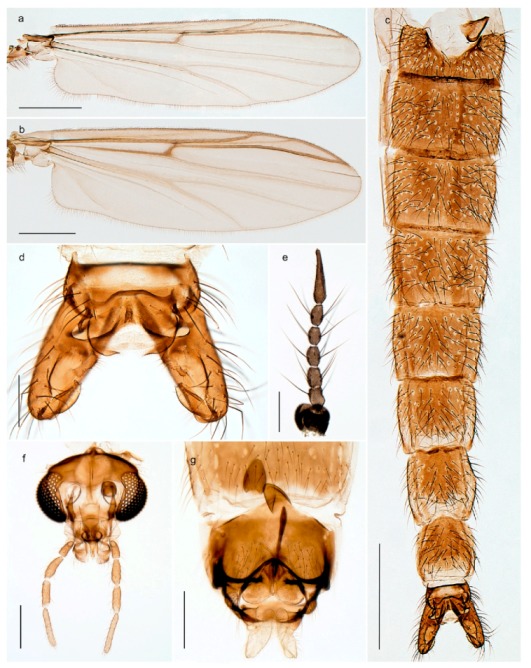
*Cricotopus* (*Cricotopus*) *lestralis*. (**a**) Male wing, scale bar = 500 µm; (**b**) female wing, scale bar = 500 µm; (**c**) male abdomen, scale bar = 500 µm; (**d**) male hypopygium, scale bar = 100 µm; (**e**) female antenna, scale bar = 100 µm; (**f**) female head, scale bar = 200 µm; (**g**) female genitalia, scale bar = 200 µm.

**Figure 28:**
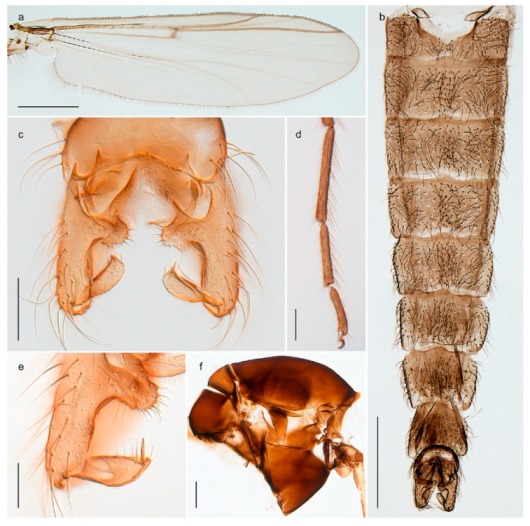
*Cricotopus* (*Cricotopus*) *pilosellus*. (**a**) Male wing, scale bar = 500 µm; (**b**) male abdomen, scale bar = 500 µm; (**c**) male hypopygium, scale bar = 100 µm; (**d**) male fore tarsus, scale bar = 100 µm; (**e**) male gonocoxite and gonostylus, scale bar = 50 µm; (**f**) male thorax, scale bar = 200 µm.

**Figure 29:**
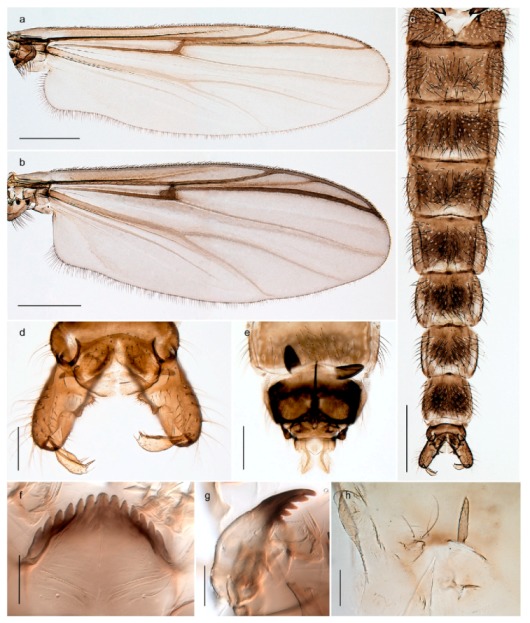
*Cricotopus* (*Cricotopus*) *tibialis*. (**a**) Male wing, scale bar = 500 µm; (**b**) female wing, scale bar = 500 µm; (**c**) male abdomen, scale bar = 500 µm; (**d**) male hypopygium, scale bar = 100 µm; (**e**) female genitalia, scale bar = 200 µm; (**f**) larval mentum, scale bar = 50 µm; (**g**) larval mandible, scale bar = 50 µm; (**h**) pupal thoracic horn, scale bar = 100 µm.

**Figure 30:**
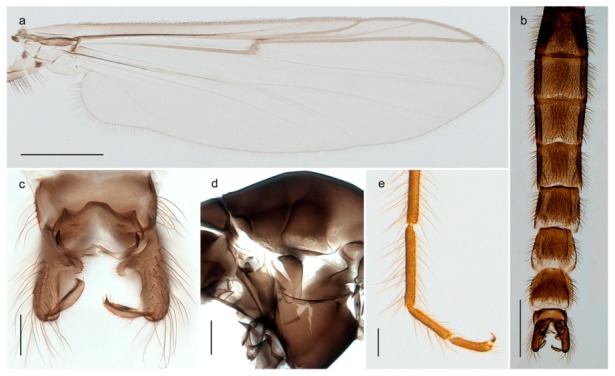
*Cricotopus* (*Cricotopus*) *villosus*. (**a**) Male wing, scale bar = 500 µm; (**b**) male abdomen, scale bar = 500 µm; (**c**) male hypopygium, scale bar = 100 µm; (**d**) male thorax, scale bar = 200 µm; (**e**) male fore tarsus, scale bar = 100 µm.

**Figure 31:**
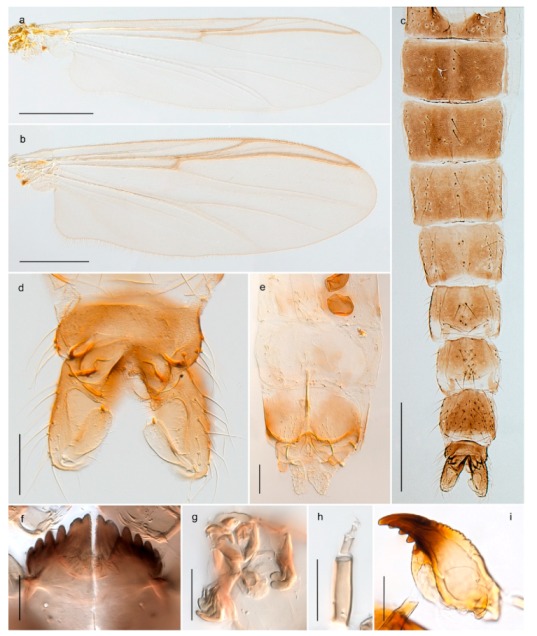
*Cricotopus* (*Isocladius*) *glacialis*. (**a**) Male wing, scale bar = 500 µm; (**b**) Female wing, scale bar = 500 µm; (**c**) male abdomen, scale bar = 500 µm; (**d**) male hypopygium, scale bar = 100 µm; (**e**) female genitalia, scale bar = 100 µm; (**f**) larval mentum, scale bar = 50 µm; (**g**) larval premandible, scale bar = 50 µm; (**h**) larval antenna, scale bar = 50 µm; (**i**) larval mandible, scale bar = 50 µm.

**Figure 32:**
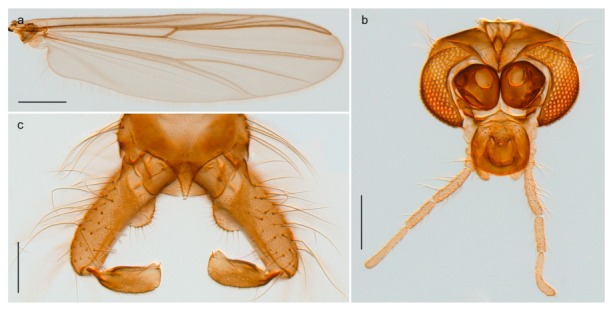
*Heterotrissocladius subpilosus*. (**a**) Male wing, scale bar = 500 µm; (**b**) male head, scale bar = 200 µm; (**c**) male hypopygium, scale bar = 100 µm.

**Figure 33:**
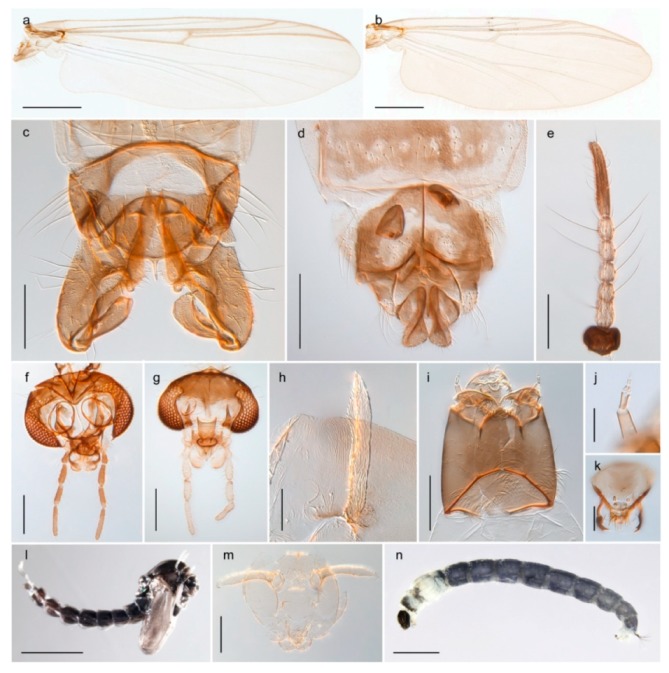
*Hydrobaenus conformis*. (**a**) Male wing, scale bar = 500 µm; (**b**) Female wing, scale bar = 500 µm; (**c**) male hypopygium, scale bar = 100 µm; (**d**) female genitalia, scale bar = 200 µm; (**e**) female antenna, scale bar = 100 µm; (**f**) male head, scale bar = 100 µm; (**g**) female head, scale bar = 100 µm; (**h**) pupal thoracic horn, scale bar = 100 µm; (**i**) larval head ventral view, scale bar = 100 µm; (**j**) larval antenna, scale bar = 25 µm; (**k**) larval labral surface, scale bar = 25 µm; (**l**) pupa lateral view, scale bar 1 mm; (**m**) pupal frontal apotome, scale bar = 200 µm; (**n**) larva lateral view, scale bar = 1 mm.

1. Abdominal tergites with reduced setation, forming longitudinal rows on segments III-V (Figure 31c); female with humeral setae; male hypopygium with superior volsella (Figure 31d)*Cricotopus* (*Isocladius*) *glacialis*- Abdominal tergites more or less covered with setae, no longitudinal rows on segments II-V (Figures 26b and 27c and 28b and 29c and 30b); female without humeral setae; male hypopygium without superior volsella (e.g., Figure 26c) (subgenus Cricotopus)22. Anterior prealar setae present, slightly smaller than posterior prealar setae (but not separated from these) (Figure 28f and 30d)3- Anterior prealar setae absent. posterior prealar setae present43. Bristle ratio on third tarsomere of fore leg > 3.5 (Figure 30e); 0- 10 setae on preepisternum*Cricotopus* (*C.*) *villosus*- Bristle ratio on third tarsomere of fore leg < 3.5 (Figure 28d); more than 14 setae on preepisternum (Figure 28f)*Cricotopus* (*C.*) *pilosellus*4. Setation on abdominal tergites slightly reduced, anteromedian areas of tergites III-IV with seta-free patches (Figure 26b)*Cricotopus* (*C.*) *gelidus*- Setation on abdominal tergites not reduced, setae on tergites III-IV evenly distributed (Figures 27c and 29c)55. Legs completely brown; male superior volsella broadly rectangular, simple (Figure 27d)*Cricotopus* (*C.*) *lestralis*- Legs with pale ring on tibiae (not obvious in freshly emerged individuals); male inferior volsella usually with obvious concave median margin (Figure 29d)*Cricotopus* (*C.*) *tibialis*

#### 4.6.7. Heterotrissocladius

Concerning records previously identified as *Heterotrissocladius callosus* (Becher, 1886) please see under *Metriocnemus* below.

*Heterotrissocladius subpilosus* (Kieffer, 1911) was described by Kieffer in Koenig ([71]: p. 273) from Bear Island as *Dactylocladius subpilosus*. We have examined material collected on Bear Island identified by Edwards [31] and can confirm that this material is conspecific with current understanding of *H*. *subpilosus* as described by Brundin [110] with a strongly swollen clypeus in the adult male. Photos and DNA barcodes of the *H. subpilosus* presented here are from specimens collected in Central Norway.

#### 4.6.8. Hydrosmittia

The genus *Hydrosmittia* currently has one nominal species recorded from Svalbard, but DNA barcode data indicate that there is an additional un-clarified species present. Of both species, only females were collected.

**Figure 34:**
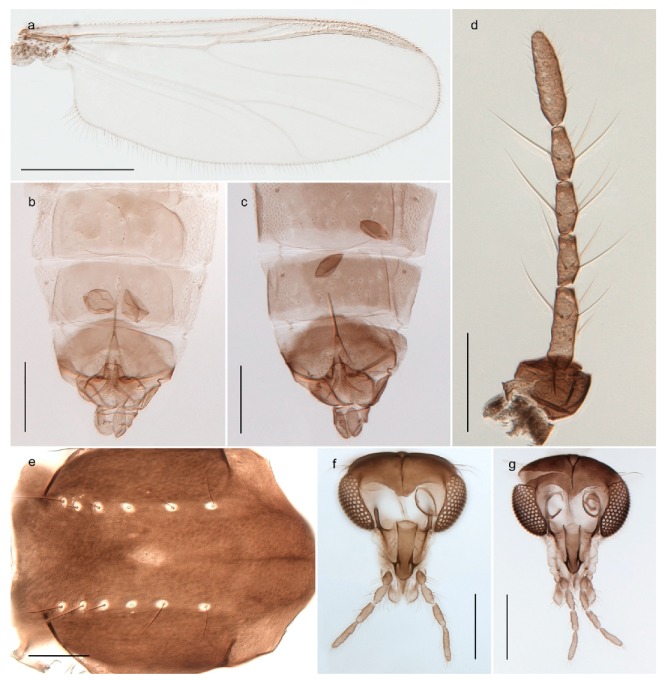
*Hydrosmittia* sp. 1ES. (**a**) Female wing, scale bar = 500 µm; (**b**) female genitalia, scale bar = 200 µm; (**f**) female head, scale bar = 200 µm. *Hydrosmittia oxoniana*. (**c**) Female genitalia, scale bar = 200 µm; (**d**) female antenna, scale bar = 100 µm; (**e**) female thorax dorsal view, scale bar = 100 µm; (**g**) female head, scale bar = 200 µm.

*Hydrosmittia oxoniana* (Edwards, 1922) (Figure 34c–e,g) was originally described as *Camptocladius oxonianus* based on females from Bear Island. Hirvenoja [33] reported females from Spitsbergen. Later records include males and indicate a wide distribution throughout the Holarctic [48]. We have only recorded females from Bear Island and Spitsbergen and DNA barcodes of these specimens constitute a well-separated cluster compared to other *Hydrosmittia* from Svalbard and mainland Norway, including a male *H. oxoniana* from Central Norway identified by Ole A. Sæther. The species is listed with a total of 9 junior synonyms [48]. Thus, we suspect that there are several unrecognized species currently hidden within *H. oxoniana* sensu Ferrington and Sæther (2011), but that our sampled populations from Bear Island (locus typicus) and Spitsbergen belong to the nominal species.

*Hydrosmittia ruttneri* occurs on Spitsbergen according to Ferrington and Sæther [48], but we have been unable to verify the source of this record and have never seen material of this species from Svalbard. The record might have been kept by a lapsus following the authors’ interpretation of Edwards’ [111] identification of *Smittia oxoniana* from Lapland. Edwards had considered the latter as conspecific with his specimens of the same species from Spitsbergen but Ferrington and Sæther [48] explicitly disagreed and stated that the specimens Edwards had identified from Lapland do not belong to the same species as those from Spitsbergen. We regard *H. ruttneri* as absent from Svalbard.

*Hydrosmittia* sp. 1ES (Figure 34a,b,f) is morphologically similar to *H. oxoniana*, but DNA barcodes constitutes a genetic cluster that is clearly divergent from those of the latter species. We suspect that the specimens represent a second species, but morphological confirmation including comparison with type material for the many synonyms of *H. oxoniana* is needed to be certain.

#### 4.6.9. Limnophyes

The genus *Limnophyes* has four confirmed species on Svalbard (see key below). An additional species, *Limnophyes edwardsi*, has been recorded from ecological studies in Ny Ålesund by Lods-Crozet, et al. [88] and Marziali, et al. [108]. Revision of this material kindly sent by B. Lods-Crozet showed that the specimens fit the description of *L. brachytomus* in Sæther [55] and that Sæther, who had identified the specimens, wrote “? edwardsi” on the slides. Sæther [55] also listed Spitsbergen as part of the distribution range for *L. edwardsi*, but there are no records of specimens in his long list of examined material and no references to relevant literature. Sæther [55] refered to Edwards’ [106] interpretation of *L. pumilio*, but only listed Edwards’ material from Scotland as examined. We therefore regard *L. edwardsi* not to be present in Svalbard.

##### Key to Species


**Figure 35:**
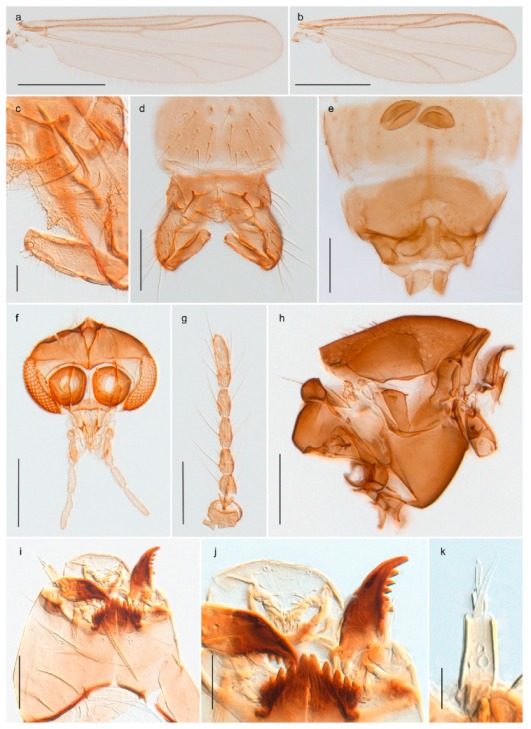
*Limnophyes brachytomus*. (**a**) Male wing, scale bar = 500 µm; (**b**) female wing, scale bar = 500 µm; (**c**) male gonocoxite and gonostylus, scale bar = 20 µm; (**d**) male hypopygium dorsal view, scale bar = 100 µm; (**e**) female genitalia, scale bar = 100 µm; (**f**) male head, scale bar = 200 µm; (**g**) female antenna, scale bar = 100 µm; (**h**) male thorax, scale bar = 200 µm; (**i**) larval head, scale bar = 100 µm; (**j**) larval mandible and mentum, scale bar = 50 µm; (**k**) larval antenna, scale bar = 20 µm.

**Figure 36:**
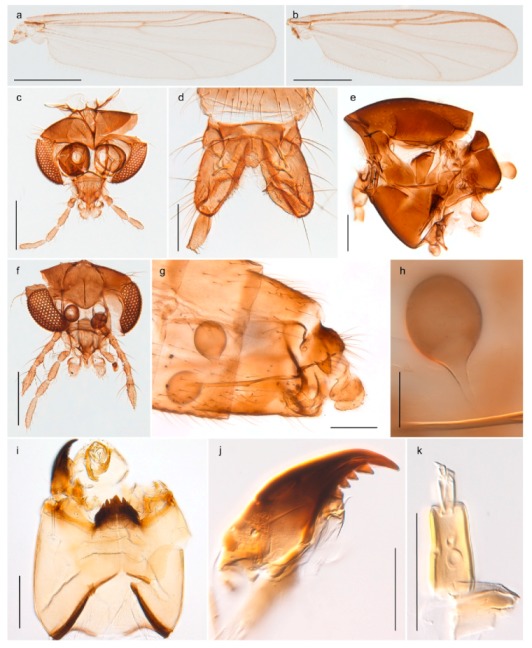
*Limnophyes eltoni*. (**a**) Male wing, scale bar = 500 µm; (**b**) female wing, scale bar = 500 µm; (**c**) male head, scale bar = 200 µm; (**d**) male hypopygium dorsal view, scale bar = 100 µm; (**e**) male thorax, scale bar = 200 µm; (**f**) female head, scale bar = 200 µm; (**g**) female genitalia lateral view, scale bar = 100 µm; (**h**) female seminal capsule, scale bar = 50 µm; (**i**) larval head, scale bar = 100 µm; (**j**) larval mandible, scale bar = 50 µm; (**k**) larval antenna, scale bar = 50 µm.

**Figure 37:**
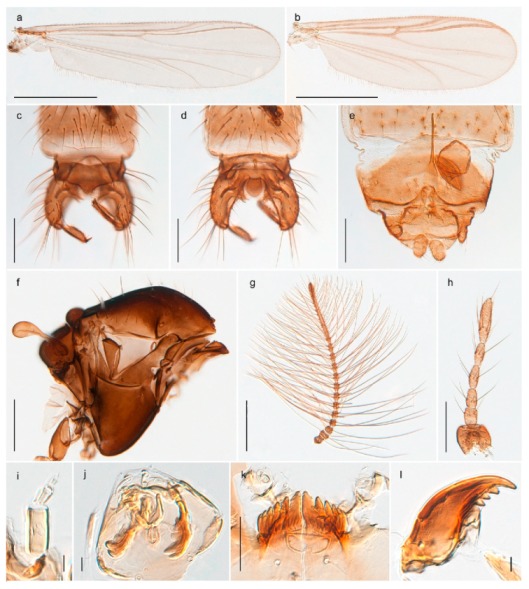
*Limnophyes pumilio*. (**a**) Male wing, scale bar = 500 µm; (**b**) female wing, scale bar = 500 µm; (**c**) male hypopygium dorsal view, scale bar = 100 µm; (**d**) male hypopygium inner structures, scale bar = 100 µm; (**e**) female genitalia, scale bar = 100 µm; (**f**) male thorax, scale bar = 200 µm; (**g**) male antenna, scale bar = 200 µm; (**h**) female antenna, scale bar = 100 µm; (**i**) larval antenna, scale bar = 10 µm; (**j**) larval premandibles, scale bar = 10 µm; (**k**) larval mentum, scale bar = 50 µm; (**l**) larval mandible, scale bar = 10 µm.

**Figure 38:**
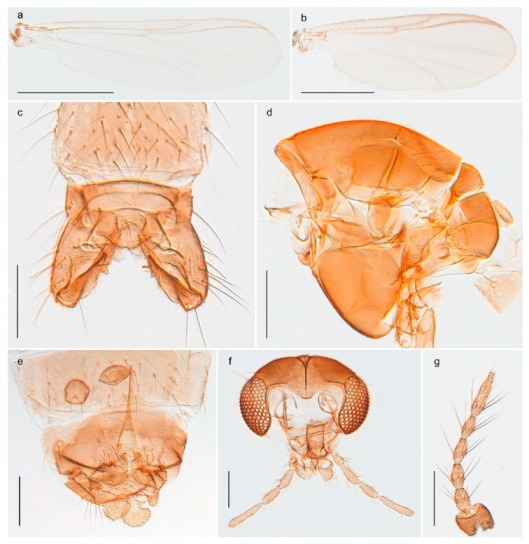
*Limnophyes schnelli*. (**a**) Male wing, scale bar = 500 µm; (**b**) female wing, scale bar = 500 µm; (**c**) male hypopygium dorsal view, scale bar = 100 µm; (**d**) male thorax, scale bar = 200 µm; (**e**) female genitalia, scale bar = 100 µm; (**f**) female head, scale bar = 100 µm; (**g**) female antenna, scale bar = 100 µm.

1. Anterior and posterior setae present on preepisternum (Figures 36e and 37f)2- Setae present only posteriorly on preepisternum (Figures 35h and 38d)32. Male genitalia with globular lobe (pars ventralis) in between gonocoxites (Figure 37d); thorax in both sexes with few lanceolate humerals and prescutellars (Figure 37f)
*Limnophyes pumilio*
- Male genitalia without globular lobe (pars ventralis) in between gonocoxites; thorax in both sexes with numerous lanceolate humerals and prescutellars (Figure 36e)
*Limnophyes eltoni*
3. Dorsocentrals long and simple in a single row (Figure 38d)
*Limnophyes schnelli*
- Dorsocentrals in multiple rows including both simple and lanceolate setae (Figure 35h)
*Limnophyes brachytomus*



*Limnophyes pumilio* (Holmgren, 1869) was described based on material from Spitsbergen collected at Green Harbour, Advent Bay and Smeerenberg ([26]: p. 41). A DNA barcode cluster comprising specimens from Spitsbergen, Greenland, Arctic Canada and one specimen from Finnmark thus appears to present the true species. Additional specimens from mainland Norway, Greenland and Arctic Canada identified as *L. pumilio* in BOLD are found in three additional BINs with a maximum pairwise distance of 6.11% to the nearest neighbour. Nevertheless, we regard all these genetic clusters as members of the same species.

*Limnophyes schnelli* was first described from mountainous regions in central and western Norway [55]. We have DNA barcodes of females from Bear Island that cluster closely with male and female specimens from northern Norway (Finnmark), northern Finland and numerous localities throughout Canada (BIN BOLD:AAC9278). The species was previously known from several countries in the northern Palaearctic [86], but this is the first record of *L. schnelli* from Svalbard.

#### 4.6.10. Metriocnemus

Five named and valid *Metriocnemus* species and one hitherto undescribed species have been recorded from Svalbard with certainty (see keys below). In addition, there are four previously recorded species names, which examination of reference material has revealed misidentifications:

*Chironomus callosus* Becher, 1886 was described from Jan Mayen based on adult males and females [34]. The description is rather good for its age, including details such as an enlarged clypeus, which likely contributed to to some authors subsequentely placing the species in *Heterotrissocladius* [3]. Edwards [35] examined the type series and noted that all Becher’s specimens were freshly emerged once the colouration of which had then faded in ethanol. He thus considered only conspecific specimens collected on Jan Mayen by W. S. Bristow in 1921 to show the true, uniformly dull black colour. The observation of a few setae on the tip of the wing (in cell r_4+5_) indicated high similarity to *Heterotrissocladius subpilosus*, but Edwards [31] ruled out the latter species since the Becher species (as represented by Mr. Barstow’s specimens) has a produced costa similar to what is observed in *Metriocnemus*. Consequently, Edwards regarded *C. callosus* as a *Metriocnemus*. We have examined Becher’s types (one male and three females) on loan from Naturhistorisches Museum Wien and agree with Edwards’ conclusions. The specimens, though damaged, conform well to the current definition of *Metriocnemus ursinus* and we therefore regard *C. callosus* as a junior synonym.

*Metriocnemus picipes* was recorded by Goetghebuer [112] from Kongsfjorden. The Goetghebuer material (1923, leg. E. Hansen, deposited in NHM Oslo) has been examined and belong to *Metriocnemus ursinus*.

*Metriocnemus similis* was listed from Svalbard in Lindegaard [98], likely based on the record by Hirvenoja [33]. The species was originally described from Novaya Zemlya [113]. We have examined the holotype deposited in NHM Oslo and regard the name as a junior synonym of *Metriocnemus ursinus*. It does not belong to *Heterotrissocladius* as was suggested by Ashe and O’Connor ([3]: p. 311). We were able to extract DNA from the holotype and obtain a short COI-sequence of 103 base pairs. The mini-barcode matches our *Metriocnemus ursinus* specimens from Svalbard by 99.02%.

The only record of *Metriocnemus tristellus* from Svalbard known to uswas published by Goetghebuer [112] from the island of Hopen. The antennae are missing from the examined male (NHM Oslo), but the general morphology and hypopygium fits *Metriocnemus* sp. 1ES, not *M. tristellus* as described originally by Edwards [106] and subsequently by Langton and Pinder [114] where the palpi were reported to be unusually short, with palpomeres 3 and 4 less than three times as long as broad and with the anal point rather long and slender [114]: Figure 168B. Thus, we regard the previous presence of *M. tristellus* in the list of Svalbard Chironomidae as erroneous due to misidentification.

##### Key to Males

**Figure 39:**
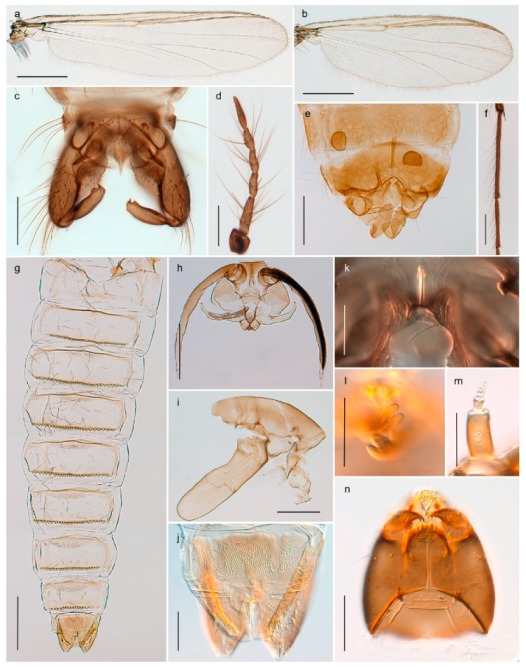
*Metriocnemus brusti*. (**a**) Male wing, scale bar = 500 µm; (**b**) female wing, scale bar = 500 µm; (**c**) male hypopygium, scale bar = 100 µm; (**d**) female antenna, scale bar = 100 µm; (**e**) female genitalia, scale bar = 200 µm; (**f**) male hind tarsus with pseudospurs, scale bar = 200 µm; (**g**) pupal abdomen, scale bar = 500 µm; (**h**) pupal frontal apotome, scale bar = 200 µm; (**i**) pupal thorax, scale bar = 500 µm; (**j**) pupal anal lobe, scale bar = 100 µm; (**k**) male hypopygium, virga, scale bar = 50 µm; (**l**) larval premandibles, scale bar = 50 µm; (**m**) larval antenna, scale bar = 50 µm; (**n**) larval head ventral view, scale bar = 100 µm.

**Figure 40:**
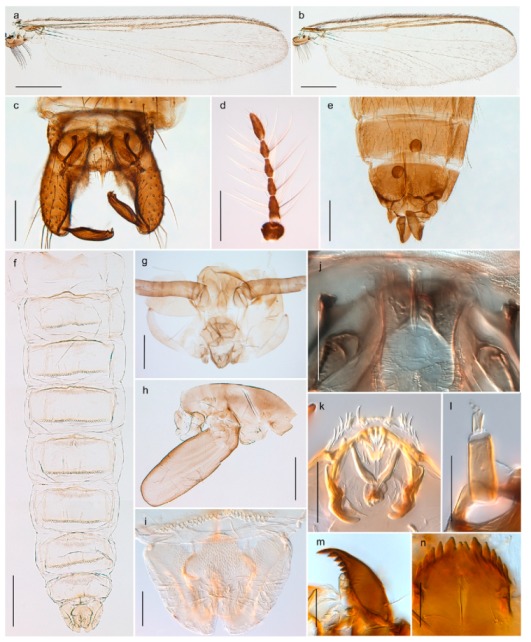
*Metriocnemus eurynotus*. (**a**) Male wing, scale bar = 500 µm; (**b**) female wing, scale bar = 500 µm; (**c**) male hypopygium, scale bar = 100 µm; (**d**) female antenna, scale bar = 200 µm; (**e**) female genitalia, scale bar = 200 µm; (**f**) pupal abdomen, scale bar = 500 µm; (**g**) pupal frontal apotome, scale bar = 200 µm; (**h**) pupal thorax, scale bar = 500 µm; (**i**) pupal anal lobe, scale bar = 100 µm; (**j**) male hypopygium, virga, scale bar = 50 µm; (**k**) larval labral surface, scale bar = 50 µm; (**l**) larval antenna, scale bar = 50 µm; (**m**) larval mandible, scale bar = 50 µm; (**n**) larval mentum, scale bar = 50 µm.

**Figure 41:**
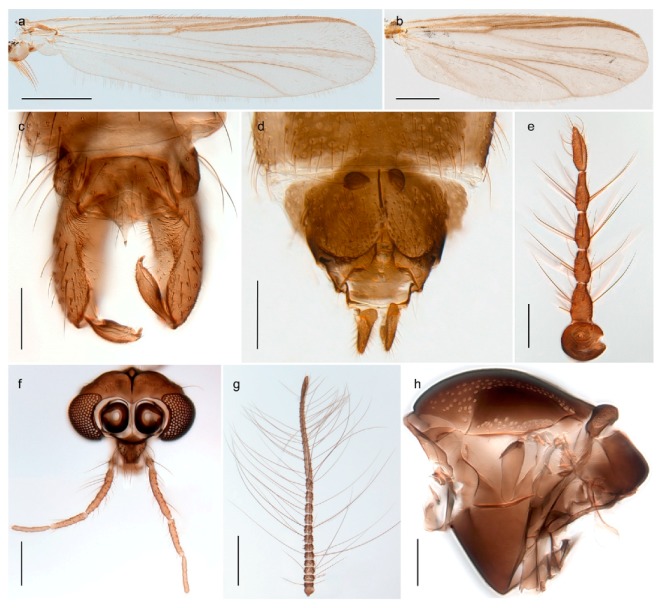
*Metriocnemus fuscipes*. (**a**) Male wing, scale bar = 500 µm; (**b**) female wing, scale bar = 500 µm; (**c**) male hypopygium, scale bar = 100 µm; (**d**) female genitalia, scale bar = 200 µm; (**e**) female antenna, scale bar = 100 µm; (**f**) male head, scale bar = 200 µm; (**g**) male antenna, scale bar = 200 µm; (**h**) male thorax, scale bar = 200 µm.

**Figure 42:**
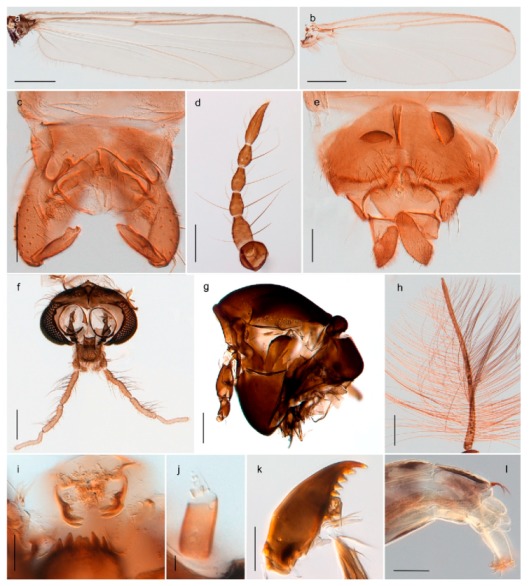
*Metriocnemus ursinus*. (**a**) Male wing, scale bar = 500 µm; (**b**) female wing, scale bar = 500 µm; (**c**) male hypopygium dorsal view, scale bar = 100 µm; (**d**) female antenna, scale bar = 100 µm; (**e**) female genitalia, scale bar = 100 µm; (**f**) male head, scale bar = 200 µm; (**g**) male thorax, scale bar = 200 µm; (**h**) male antenna, scale bar = 200 µm; (**i**) larval labral surface, scale bar = 50 µm; (**j**) larval antenna, scale bar = 10 µm; (**k**) larval mandible, scale bar = 50 µm; (**l**) larva posterior end, scale bar = 200 µm.

**Figure 43:**
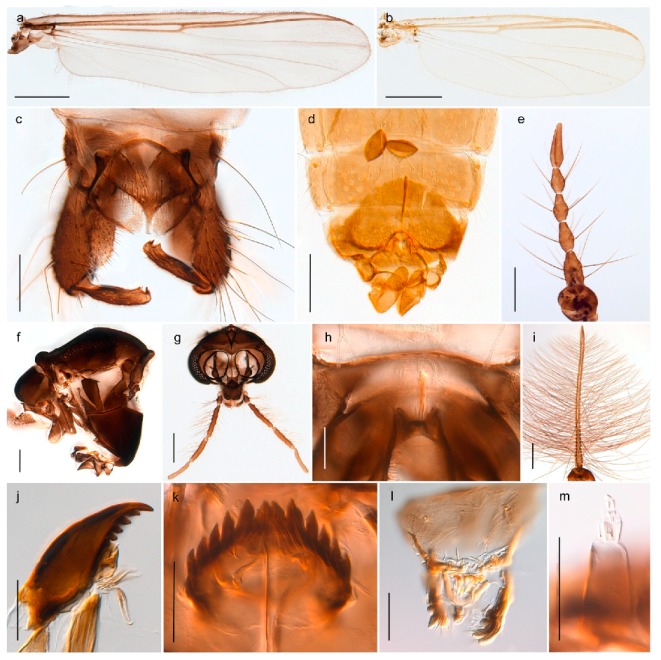
*Metriocnemus* sp. 1ES. (**a**) Male wing, scale bar = 500 µm; (**b**) female wing, scale bar = 500 µm; (**c**) male hypopygium, scale bar = 100 µm; (**d**) female genitalia, scale bar = 200 µm; (**e**) female antenna, scale bar = 100 µm; (**f**) male thorax, scale bar = 200 µm; (**g**) male head, scale bar = 200 µm; (**h**) male hypopygium, virga, scale bar = 50 µm; (**i**) male antenna, scale bar = 200 µm; (**j**) larval mandible, scale bar = 50 µm; (**k**) larval mentum, scale bar = 50 µm; (**l**) larval labral surface, scale bar = 50 µm; (**m**) larval antenna, scale bar = 50 µm.

1. Wing membrane with setae on wing tip only (Figures 42a,b and 43a)2- Wing membrane with setae on most of surface (Figures 39a and 40a and 41a)32. Hypopygium with narrow anal point (Figure 42c); head with numerous (>20) temporal setae in multiple rows; AR ca. 2.7
*Metriocnemus ursinus*
- Hypopygium often with broader, blunt anal point (Figure 43c); head usually with <15 temporal setae in one row; AR 1.8–2.2*Metriocnemus* sp. 1ES3. Hypopygium (Figure 41c) with virga absent; AR < 1.2 (Figure 41g)
*Metriocnemus fuscipes*
- Hypopygium with virga present (Figures 39k and 40j); AR > 1.744. Wing very densely clothed with setae in all cells; subcostal with more than 20 setae; costa ends clearly beyond M3+4 (Figure 40a)
*Metriocnemus eurynotus*
- Wings with setae in all cells, but much less densely so, in cell m3+4 only in apical half; subcostal with 0–8 setae; costa ends only slightly beyond apex of M3+4 (Figure 39a)
*Metriocnemus brusti*


##### Key to feMales

1. Terminal antennal flagellomere with pointed apex (Figure 42d)
*Metriocnemus ursinus*
- Terminal antennal flagellomere with rounded apex (Figures 39d, 40d, 41e and 43e)22. Antennal flagellomere 2 shorter than flagellomere 4 (Figure 41e)
*Metriocnemus fuscipes*
- Antennal flagellomere 2 at least as long as flagellomere 4 (e.g., Figure 43e)33. Antennal flagellomere 2 almost as long as flagellomere 1, with long well-defined neck (figure 17 in [27])
*Metriocnemus cataractarum*
- Antennal flagellomere 2 clearly shorter than flagellomere 1, neck often visible, but not as well defined (e.g., Figure 43e)44. Cerci with strong ventral projections; seminal capsules > 100 µm in diameter (Figure 43d)*Metriocnemus* sp. 1ES- Cerci without strong ventral projections; seminal capsules ca. 60–80 µm in diameter (Figures 39e and 40e)55. Wing membrane densely clothed with setae (Figure 40b); antepronotum usually with > 20 setae
*Metriocnemus eurynotus*
- Wing membrane not densely clothed with setae (Figure 39b); antepronotum with ca. 16 setae
*Metriocnemus brusti*


*Metriocnemus brusti* was originally described from Churchill, Manitoba and has been recorded in the eastern Palaearctic, Novaya Zemlya and Switzerland in addition to the Nearctic region [86]. DNA barcodes from our specimens collected on Spitsbergen and Edge Island cluster nicely with those from the locus typicus as well as from Greenland, northern Norway and a number of other northern localities in Canada. Our specimens also fit well with the original description. Our records are the first of this species from Svalbard.

*Metriocnemus cataractarum* Kieffer, 1919 was described from Svalbard based on females, Oliver [51] reported males from Bear Island, but we regard this association as doubtful (see comment on *Metriocnemus* sp. 1ES below). Edwards [30] recorded doubtfully identified males from Reinsdyrsflya and Roosneset, north on Spitsbergen. Thus, certainly associated males of this species are unknown to us. We have DNA barcoded *Metriocnemus* larvae from Spitsbergen (*Metriocnemus* sp. 8ES, see below) with no related sequences from adult specimens in BOLD. These might represent *M. cataractarum* or a hitherto unknown species from Svalbard.

*Metriocnemus eurynotus* (Holmgren, 1883) was originally described from Vaygach Island south of Novaya Zemlya and has been regarded as a widely distributed species [54,56] (sub *M. obscuripes* in Sæther [54]). Our DNA barcode data from the Palaearctic region form five defined barcode clusters and indicate several cryptic species in this group. This is perhaps not surprising given the list of currently held junior synonyms [54,56]. All specimens from Svalbard fall within the same genetic barcode cluster.

*Metriocnemus fuscipes* is considered widespread in the Holarctic Region, but was previously unreported from Svalbard [86,92]. Goetghebuer [112] recorded the species from eastern Greenland. Our specimens from Spitsbergen and Bear Island clusters with specimens from mainland Norway and south of Bonn in Germany (close to locus typicus for the nominotypical species) (BIN BOLD:AAI1573). Additional DNA barcodes from other populations in Norway and the German Alps form an additional three clusters that are genetically deeply divergent from this group.

*Metriocnemus* sp. 1ES differs from other described *Metriocnemus* species by having males with a combination of reduced wing setation, few temporal setae on the head, AR between 1.8–2.2 and a slightly broader and blunt anal point. The species seems to be quite similar to Oliver’s [51] interpretation of *M. cataractarum* Kieffer, 1919. However, the latter was based on females only and our DNA barcode associated females of *M.* sp. 1ES do not fit Kieffer’s description [27] as they have quite differently structured antennae with shorter flagellomeres (Figure 43e).

**Figure 44:**
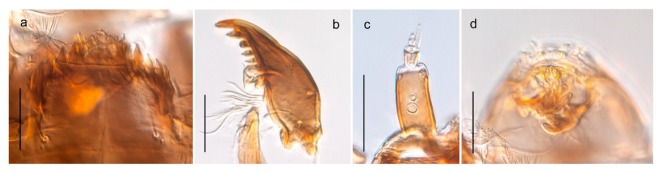
*Metriocnemus* sp. 8ES. (**a**) larval mentum, scale bar = 50 µm; (**b**) larval mandible, scale bar = 50 µm; (**c**) larval antenna, scale bar = 50 µm; (**d**) larval premandibles, scale bar = 50 µm.

*Metriocnemus* sp. 8ES (Figure 44) was collected only as larvae from northern Spitsbergen. The species belongs to the *eurynotus* group [84], but we have been unable to match it with any other species present in BOLD. There are two *Metriocnemus* species of the *eurynotus* group that have Arctic distribution but are unrepresented in BOLD. *Metriocnemus longipennis* (Holmgren, 1883), a peculiar species with reduced wings, antennae and thorax, was described from Novaya Zemlya and later recorded and redescribed from New Siberia [56,57,107] and northernmost Alaska [54]. *Metriocnemus sibiricus* (Lundström, 1915) was described from the New Siberian Islands and redescribed by Sæther [54]. The male of the latter species resembles *M. longipennis* in its shortened wings and reduced antennal plume, but differs by the shape of the gonostylus, by normal wing setation and the higher number of flagellomeres.

#### 4.6.11. Orthocladius

*Orthocladius* is represented by eight nominal valid species in Svalbard. In addition, we have material of one species that is putatively new to science. Four of the presently six subgenera have been recorded.

The names *Orthocladius arcticus* (Kieffer, 1919) and *Orthocladius spitzbergensis*, both with type localities in Spitsbergen [27] are regarded as nomina dubia, probably in the subgenus *Eudactylocladius* [3]. *Orthocladius mixtus* (Holmgren, 1869) with type locality in Bear Island [26] has been regarded as a nomen dubium in *Orthocladius* s. str. [3,115], but is revived (below).

*Orthocladius* (*Eudactylocladius*) *gelidus* was first recorded by Hirvenoja [33] from Helvetiafjellet on Spitsbergen. The material could not be located at the Finnish Museum of Natural History in Helsinki. Given that later taxonomic papers have both, revised definitions in the genus and added species to the fauna of Svalbard [58,59,115], we regard the record in Hirvenoja [33] as doubtful. The species was later recorded from the stream Londonelva near Ny-Ålesund on Spitsbergen [88]. We have examined an adult male from this material kindly sent to us by B. Lods-Crozet and find it to be conspecific with our material of *O*. (*E*.) *gelidorum* from Svalbard as well as with the redescription of the latter species by Cranston [115]. We thus regard *O*. (*E*.) *gelidus* as hitherto undocumented from Svalbard.

*Orthocladius* (*Euorthocladius*) *thienemanni* has been reported in an ecological study from Ny Ålesund [88,108], but re-examination of a pupal skin from this material kindly sent to us by B. Lods-Crozet showed that it fits our associated pupae of *Orthocladius* (*Euorthocladius*) *telochaetus* Langton, 1985 (associated here for the first time). We thus regard the previous record of *O.* (*Euorthocladius*) *thienemanni* in Svalbard to be a misidentification.

*Orthocladius* (*Orthocladius*) *obumbratus* was recorded from Svalbard by Lindegaard [98], possibly based on the suggested synonymy with *O*. (*O*.) *rhyacobius*, *O*. (*O*.) *dispar* Goetghebuer, 1942 and *O*. (*O*.) *excavatus* Brundin, 1947 published by Langton and Cranston [116] and on the wide distribution of *O*. (*O*.) *excavatus* recorded by Soponis [72]. The synonymy was later dissolved by Rossaro, et al. [117] (see comments by Spies and Sæther [118]). We are unaware of any reliable documentation of *O*. (*O*.) *obumbratus* from Svalbard, a species which, according to both Soponis [72] and Rossaro, et al., [117] has been found only in the Nearctic Region.

*Orthocladius* (*Orthocladius*) *rhyacobius* was originally described based on material collected by Thienemann in Germany [119]. It was regarded as a distinct species by Rossaro, et al. [117], but their taxonomic treatment of this and associated species was questioned by Spies and Sæther [118]. The species was recorded as adult males by Lods-Crozet, et al. [88] from Bayelva near Ny-Ålesund on Spitsbergen. We have examined two poorly preserved specimens from this material and find them conspecific with *O*. (*Pogonocladius*) *consobrinus* (Holmgren, 1869). Thus, we do not regard *O.* (*O*.) *rhyacobius* to be present on Svalbard.

*Orthocladius* (*Orthocladius*) *trigonolabis* Edwards, 1924 was originally described from northern Spitsbergen [29] and the name is frequently seen in faunistic literature on Svalbard. Sæther [57] found this name to be a junior synonym of *O*. (*O*.) *nitidoscutellatus* Lundström, 1915 (originally described from northern Russia) and later described larvae of this species from Svalbard [59].

##### Key to Males

**Figure 46:**
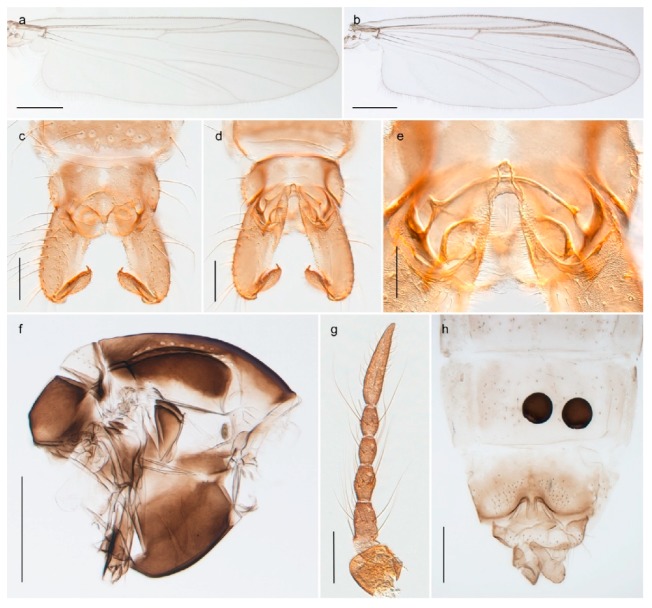
*Orthocladius* (*Eudactylocladius*) *gelidorum*. (**a**) Male wing, scale bar = 500 µm; (**b**) female wing, scale bar = 500 µm; (**c**) male hypopygium, dorsal view, scale bar = 100 µm (**d**) male hypopygium, ventral view, scale bar = 100 µm; (**e**) male hypopygium inner structures, scale bar = 50 µm; (**f**) female thorax, scale bar = 500 µm; (**g**) female antenna, scale bar = 100 µm; (**h**) female genitalia, scale bar = 200 µm.

**Figure 47:**
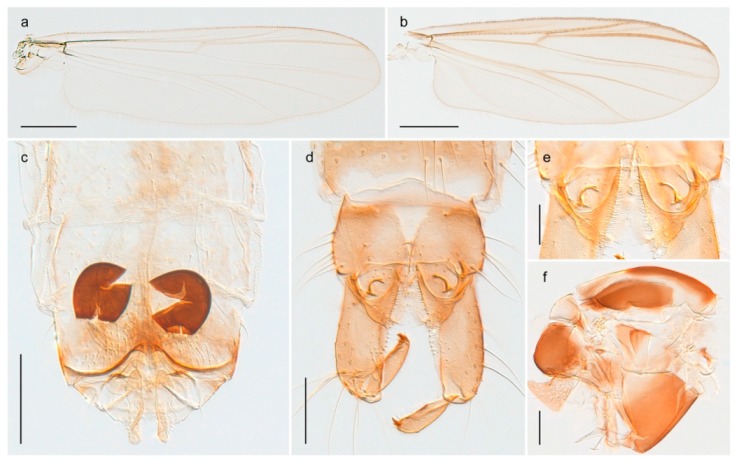
*Orthocladius* (*Eudactylocladius*) sp. 2TE. (**a**) Male wing, scale bar = 500 µm; (**b**) female wing, scale bar = 500 µm; (**c**) female genitalia, scale bar = 200 µm; (**d**) male hypopygium, scale bar = 100 µm; (**e**) male hypopygium inner structures, scale bar = 50 µm; (**f**) male thorax, scale bar = 200 µm.

**Figure 48:**
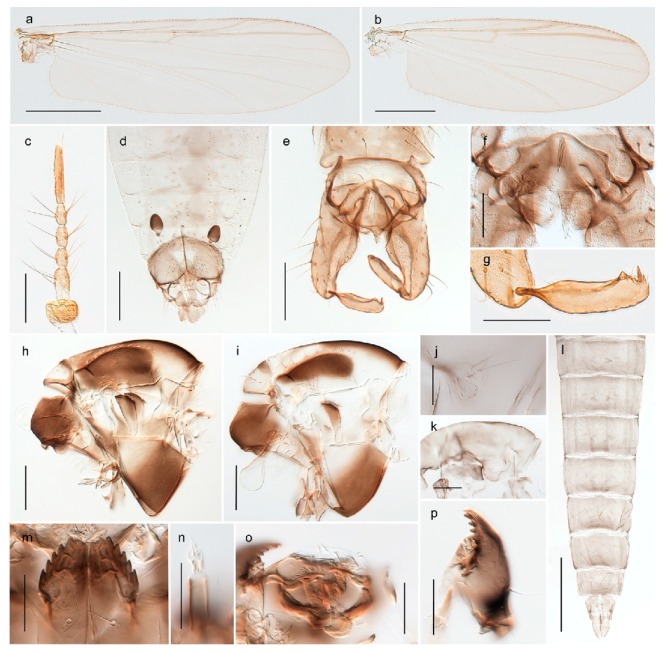
*Orthocladius* (*Euorthocladius*) *telochaetus*. (**a**) Male wing, scale bar = 500 µm; (**b**) female wing, scale bar = 500 µm; (**c**) female antenna, scale bar = 100 µm; (**d**) female genitalia, scale bar = 200 µm; (**e**) male hypopygium, scale bar = 100 µm; (**f**) male hypopygium inner structures, scale bar = 50 µm; (**g**) male gonostylus, scale bar = 50 µm; (**h**) male thorax, scale bar = 200 µm; (**i**) female thorax, scale bar = 200 µm; (**j**) pupal precorneal setae, scale bar= 50 µm; (**k**) pupal thorax, scale bar = 200 µm; (**l**) pupal abdomen, scale bar = 500 µm; (**m**) larval mentum, scale bar = 50 µm; (**n**) larval antenna, scale bar = 50 µm; (**o**) larval labral surface, scale bar = 50 µm; (**p**) larval mandible, scale bar = 50 µm.

**Figure 49:**
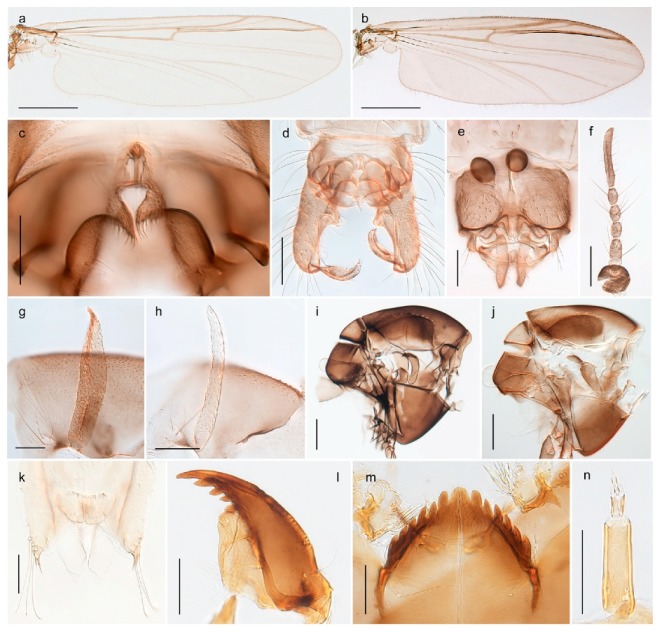
*Orthocladius* (*Orthocladius*) *decoratus*. (**a**) Male wing, scale bar = 500 µm; (**b**) female wing, scale bar = 500 µm; (**c**) male inner structures of hypopygium, scale bar = 50 µm; (**d**) male hypopygium, scale bar = 100 µm; (**e**) female genitalia, scale bar = 100 µm; (**f**) female antenna, scale bar = 100 µm; (**g**) female pupal thoracic horn, Bear Island, scale bar = 100 µm; (**h**) female pupal thoracic horn, Spitsbergen, scale bar = 100 µm; (**i**) male thorax, scale bar = 200 µm; (**j**) female thorax, scale bar = 200 µm; (**k**) pupal anal lobe, scale bar = 100 µm; (**l**) larval mandible, scale bar = 50 µm; (**m**) larval mentum, scale bar = 50 µm; (**n**) larval antenna, scale bar = 50 µm.

**Figure 50:**
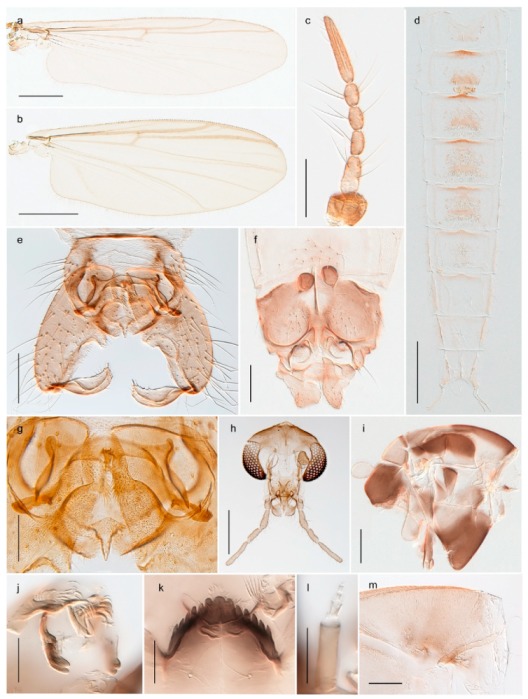
*Orthocladius* (*Orthocladius*) *mixtus*. (**a**) Male wing, scale bar = 500 µm; (**b**) female wing, scale bar = 500 µm; (**c**) female antenna, scale bar = 100 µm; (**d**) pupal abdomen, scale bar = 500 µm; (**e**) male hypopygium, scale bar = 100 µm; (**f**) female genitalia, scale bar = 100 µm; (**g**) male anal tergite and inner structures of hypopygium, scale bar = 50 µm; (**h**) female head, scale bar = 200 µm; (**i**) female thorax, scale bar = 200 µm; (**j**) larval premandible, scale bar = 50 µm; (**k**) larval mentum, scale bar = 50 µm; (**l**) larval antenna, scale bar = 50 µm; (**m**) pupal anterior thorax, scale bar = 100 µm.

**Figure 51:**
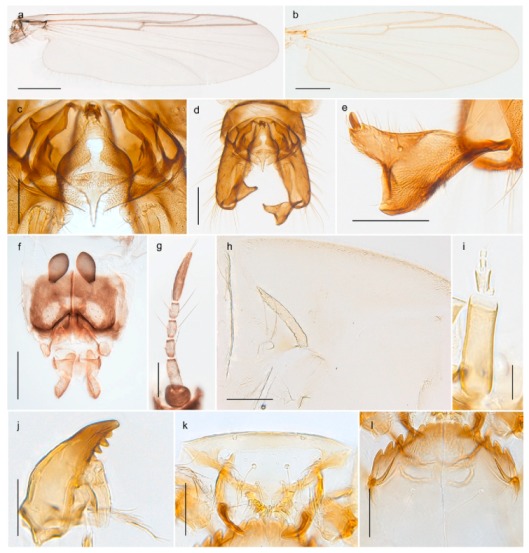
*Orthocladius* (*Orthocladius*) *nitidoscutellatus*. (**a**) Male wing, scale bar = 500 µm; (**b**) female wing, scale bar = 500 µm; (**c**) male anal tergite and inner structures of hypopygium, scale bar = 50 µm; (**d**) male hypopygium, scale bar = 100 µm; (**e**) male gonostylus, scale bar = 50 µm; (**f**) female genitalia, scale bar = 200 µm; (**g**) female antenna, scale bar = 100 µm; (**h**) pupal thorax, scale bar = 100 µm; (**i**) larval antenna, scale bar = 50 µm; (**j**) larval mandible, scale bar = 50 µm; (**k**) larval labral surface, scale bar = 50 µm; (**l**) larval mentum, scale bar = 50 µm.

**Figure 52:**
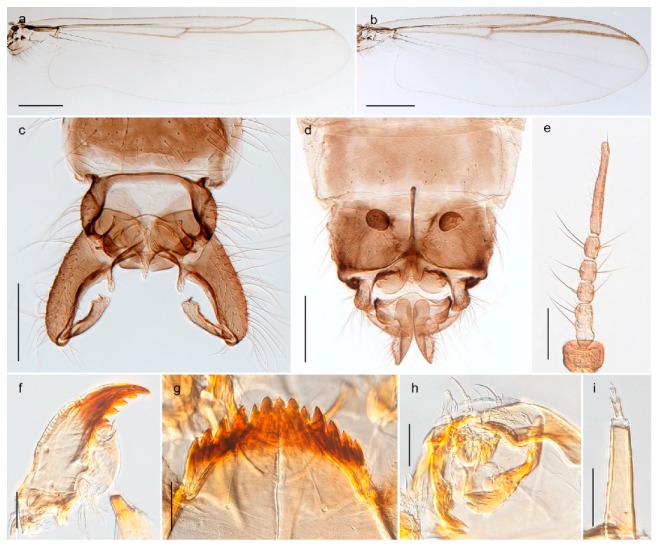
*Orthocladius* (*Pogonocladius*) *consobrinus*. (**a**) Male wing, scale bar = 500 µm; (**b**) female wing, scale bar = 500 µm; (**c**) male hypopygium, scale bar = 200 µm; (**d**) female genitalia, scale bar = 200 µm; (**e**) female antenna, scale bar = 100 µm; (**f**) larval mandible, scale bar = 50 µm; (**g**) larval mentum, scale bar = 50 µm; (**h**) larval labral surface, scale bar = 50 µm; (**i**) larval antenna, scale bar = 50 µm.

**Figure 53:**
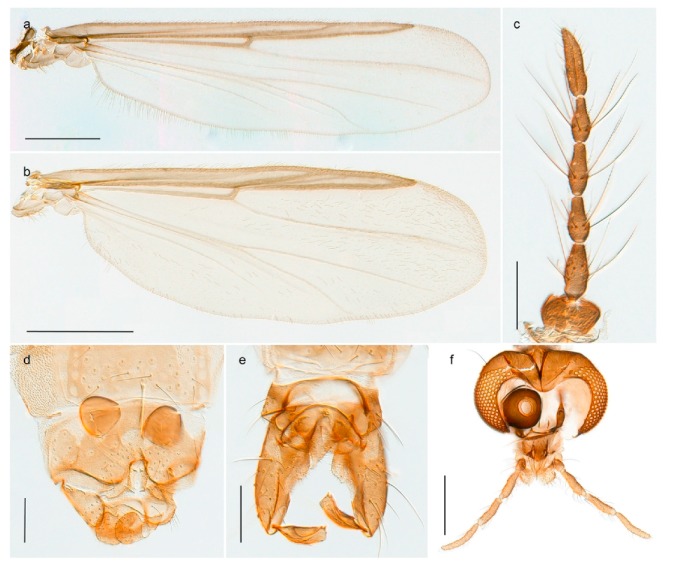
*Paraphaenocladius brevinervis*. (**a**) Male wing, scale bar = 500 µm; (**b**) female wing, scale bar = 500 µm; (**c**) female antenna, scale bar = 100 µm; (**d**) female genitalia, scale bar = 100 µm; (**e**) male hypopygium, scale bar = 100 µm; (**f**) male head, scale bar = 200 µm.

1. Male gonocoxite with poorly developed inferior volsella (Figures 46d and 47e); virga absent (subgenus *Eudactylocladius*)2- Male gonocoxite with well-developed inferior volsella (e.g., Figures 49d, 50g and 51c); virga absent or present (e.g., Figures 49c and 50g); if inferior relatively volsella low, then virga present42. Anal point well sclerotized, parallel-sided (figure 2 in [58]); tarsi without sensilla chaetica*Orthocladius* (*Eudact.*) *almskari*- Anal point less sclerotized than above, if rather well sclerotized then anal point triangular; sensilla chaetica present on tarsi of mid and hind legs33. Anal point narrowly triangular, relatively well sclerotized (Figure 46c); gonostylus widest at ½ length (Figure 46c,d); hind ta1 with 1-2 sensilla chaetica*Orthocladius* (*Eudact.*) *gelidorum*- Anal point broadly triangular, weakly sclerotized (Figure 47d); gonostylus widest at 2/3 length (Figure 47d); hind ta1 with 4 sensilla chaetica*Orthocladius* (*Eudact.*) sp. 2TE4. Fore tarsus with beard; anal lobe of wing strongly produced (Figure 52a)*Orthocladius* (*Pogonocladius*) *consobrinus*- Fore tarsus without beard; anal lobe of wing obtuse or moderately produced (e.g., Figures 48a and 49a)55. Anal point often with apical seta; dorsal lobe of inferior volsella weakly developed; superior volsella broad, medially directed (Figure 48f); scutellar setae biserial*O*. (*Euorthocladius*) *telochaetus*- Anal point without apical seta; dorsal lobe of inferior volsella well-developed; superior volsella well-developed, posteriorly directed, except in O. (O.) nitidoscutellatus (Figure 51c); scutellar setae uniserial (subgenus Orthocladius)66. Outer margin of gonostylus with a large projection, giving a triangular appearance (Figure 51e)*Orthocladius* (*O*.) *nitidoscutellatus*- Outer margin of gonostylus not as above77. Anal point comparatively long, sharply pointed; gonostylus strongly curved inwards distally, giving the subapical outer margin a flat appearance margin (figure 59b in [72])*Orthocladius* (*O*.) *knuthi*- Anal point relatively shorter, triangular; gonostylus not strongly curved inwards, its subapical outer margin with rounded appearance (Figures 49d and 50e)88. Dorsal lobe of inferior volsella with comparatively broad base, giving this part a triangular to quadrangular appearance; gonostylus comparatively wide, its inner margin without microtrichia; transverse sternapodeme comparatively straight with strong anterior projections (Figure 50e)*Orthocladius* (*O*.) *mixtus*- Dorsal lobe of inferior volsella with comparatively narrow base, giving this part an almost sausage-like appearance; gonostylus comparatively narrow with microtrichia on inner margin; transverse sternapodeme comparatively curved with weak anterior projections (Figure 49d)*Orthocladius* (*O*.) *decoratus*

##### Key to Females (Female of *O*. (*Eudactylocladius*) *Almskari* Unknown)

1. Spermathecal ducts with at least one U-shaped loop2- Spermathecal ducts straight42. Tergite IX divided; seminal capsule pear-shaped (Figure 48d)*Orthocladius* (*Euorthocladius*) *telochaetus*- Tergite IX undivided; seminal capsule more or less circular (Figures 46h and 47c)33. Seminal capsule comparatively large, as long as cercus (Figure 47c)*Orthocladius* (*Eudact.*) sp. 2TE- Seminal capsule comparatively small, shorter than cercus (Figure 46h)*Orthocladius* (*Eudact.*) *gelidorum*4. Tergite IX not clearly divided, with pale margins; terminal flagellomere long, AR close to 1 (Figure 52e)*Orthocladius* (*Pogonocladius*) *consobrinus*- Tergite IX clearly divided, with darkened margins; terminal flagellomere short, AR < 0.85 (e.g., Figures 50c and 51g) (subgenus Orthocladius)55. Neck of seminal capsule transparent, not sclerotized; rami very small, in ventral view often obscured by median ends of gonocoxapodemes (Figure 51f)*Orthocladius* (*O*.) *nitidoscutellatus*- Neck of seminal capsule sclerotized; rami longer, well visible anterior to gonocoxapodemes (e.g., Figure 50f)66. Gonocoxite IX with < 10 setae; cercus with slightly pointed anterior end (Figure 50f); thoracic pigmented aresa with rather well-defined margins (Figure 50i)*Orthocladius* (*O*.) *mixtus*- Gonocoxite IX with >10 setae; cercus with rounded anterior end (Figure 49e); thoracic pigmented areas with comparatively fuzzy margins (Figures 49i,j)77. AR < 0.8 (Figure 49f); head with about 5 vertical setae*Orthocladius* (*O*.) *decoratus*- AR > 0.8; head with about 10 vertical setae*Orthocladius* (*O*.) *knuthi* (page 29, figure 76 in [72])

*Orthocladius* (*Eudactylocladius*) *almskari* was originally described as *O*. (*Eudactylocladius*) *schnelli* Sæther, 2004 from Lake Birgervatnet in north-western Spitsbergen [58]. Due to junior homonymy, Sæther in Spies and Sæther [118] renamed the species to *O*. (*Eudactylocladius*) *almskari*. The three species of *Eudactylocladius* from Svalbard are very similar in the adult male and can only be separated by minor differences in the hypopygium and the tarsal sensilla chaetica. *Orthocladius* (*Eudactylocladius*) sp. 2TE cannot be assigned to any scientifically named species and is given an interim name until more material will allow formal description. In BOLD, there are two specimens of the same BIN from Northwest Territories and Yukon (Canada), indicating that *O*. (*Eudactylocladius*) sp. 2TE is an arctic species.

*Orthocladius* (*Eudactylocladius*) sp. 2TE has been recorded from Svalbard as single female from Bear Island. However, the DNA barcode of this specimen groups with a male from Finnmark, northern Norway and enables us to separate the species from other *Orthocladius* recorded from Svalbard. The male adult keys to *Orthocladius* (*Eudactylocladius*) *sublettorum* in [58], but differs by having slightly more setae on the squama and no crista dorsalis. The DNA barcodes of *O*. (*Eudactylocladius*) sp. 2TE are more than 8% divergent from those in BOLD identified as *O*. (*Eudactylocladius*) *subletteorum* from North America, Central and northern Norway.

*Orthocladius* (*Euorthocladius*) *telochaetus* Langton, 1985 was described based on two original syntypes of *Chironomus limbatellus* Holmgren, 1869 that were not conspecific with the syntype selected as lectotype for *Psectrocladius limbatellus* (Holmgren) [63]. DNA barcodes of *Orthocladius* (*Euorthocladius*) *telochaetus* from Svalbard cluster tightly with barcodes from *Orthocladius* (*Euorthocladius*) *rivicola* Kieffer, 1911 from mainland Norway. Soponis [61] diagnosed adult males of *Orthocladius* (*Euorthocladius*) *telochaetus* by the apical seta on the anal point. We have observed specimens without this feature and also with an anal point much shorter than in the type series (Figure 48e,f). However, the very low dorsal lobe of the inferior volsella and the darker colour of the specimens seem to be good characters to separate male adults of *O*. (*Euorthocladius*) *telochaetus* from those of *O*. (*Euorthocladius*) *rivicola.* Moreover, in *O*. (*Euorthocladius*) *telochaetus* the pupal abdominal tagite TIII has spines in posterior rows (Figure 48l), whereas these are absent in *O*. (*Euorthocladius*) *rivicola*. Thus, we regard these two species as separate despite high similarity in DNA barcodes (same BIN). We regard the record of *O*. (*Euorthocladius*) *rivicola* reported as larvae in an ecological study near New Ålesund (Spitsbergen) by Blaen, et al. [120] as misidentifications due to the difficulty of separating species in *Eurorthocladius* in the larval stage [61], and to the fact that the larva of *O*. (*Euorthocladius*) *telochaetus* has been unknown.

We have not seen material of *Orthocladius* (*Orthocladius*) *knuthi* Soponis, 1977 from Svalbard, but Soponis [72] based her original description partly on a male (paratype) from Hornsund (Spitsbergen). We have seen and barcoded an adult male from Churchill (Manitoba, Canada) that fits well with the original description of this species. The characters separating females of *O*. (*O*.) *decoratus* and *O*. (*O*.) *knuthi* should be regarded as uncertain due to few examined specimens and the brief original description [72].

*Orthocladius* (*Orthocladius*) *mixtus* (Holmgren, 1869) was originally described from Bear Island based on a female adult [26]. Edwards [29] reported males from northern Spitsbergen and regarded the name as a senior synonym of *Orthocladius arcticus* Kieffer, 1919, despite differences in body colour [27,29,113]. Edwards [31] recorded two males from Bear Island with questionable identification to *O. mixtus*. We have compared our female specimens with photographs of the genitalia and critical mensural data of the holotype and find this material to be conspecific. Consequently, we can confirm the placement of *Orthocladius mixtus* (Holmgren) in the subgenus *Orthocladius* as suggested by Cranston [115]. Previously, the species name has been treated as a junior synonym of *Orthocladius* (*O*.) *decoratus* (Holmgren, 1869), and most recently as a nomen dubium in the subgenus *Orthocladius* [3]. We now regard it as denoting a separately valid, well defined species. The latter is very similar to the male, female and pupa to *Orthocladius* (*O*.) *wiensi* Sæther, 1969 [72,121], but differs in being larger (male standard wing length approx. 2.5 mm), having a lower adult male AR (1.2), more setae on the male laterosternite IX and by having fewer spinules on TIII and TVI of the pupal abdomen. DNA barcodes of our Svalbard specimens cluster tightly with specimens from the northwestern Yukon in Canada (BIN BOLD:AAE4991).

#### 4.6.12. Paralimnophyes

The genus *Paralimnophyes* has been recorded from Spitsbergen only once as a single larval head capsule, sampled with a battery-driven pooter in a kittiwake nest [85]. We have examined the specimen and find that this likely belongs to *Limnophyes* as there are only three well developed inner teeth on the mandible. We thus consider the record of *Paralimnophyes* on Svalbard as doubtful. The genus is nevertheless included in the above key for future reference. The larva is known for only one European species, and the latter is not the candidate most likely to occure on Svalbard, *Paralimnophyes trilineatus* (Lundström, 1915).

#### 4.6.13. Paraphaenocladius

*Paraphaenocladius impensus* was recorded from Spitsbergen by Hirvenoja [33]. The record went undetected by Sæther and Wang [122], who did not see material from Svalbard for their revision of the genus. We have examined the specimens on which Hirvenoja’s recordwas based and find that these belong to *Paraphaenocladius brevinervis* (Holmgren, 1869).

#### 4.6.14. Psectrocladius

The genus *Psectrocladius* is represented with five species on Svalbard. We have seen material of and DNA barcoded *P*. (*Monopsectrocladius*) *calcaratus*, *P.* (*Psectrocladius*) *octomaculatus*, *P.* (*P.*) *psilopterus*, *P*. (*P*.) *barbimanus*, *P*. (*P*.) *limbatellus* and *P* (*P*.) *oxyura*, but only the material of the latter three species was from Svalbard. Therefore, several of the diagnostic characters in the key below are retrieved from literature, in particular from [62,63,123,124].

*Psectrocladius* (*Monopsectrocladius*) *calcaratus* (Edwards, 1929) was reported by Langton [63] based on one male adult in Edwards’ material from Spitsbergen. We have examined this specimen and find it to belong to *P*. (*P*.) *limbatellus*. Since this was the only known record of *P*. (*M*.) *calcaratus* from Svalbard, we now regard this species to be absent from the archipelago. We have nevertheless included it in the below key and figures to avoid future confusion. Our understanding of this species is based on literature and DNA barcoded specimens from northern and central Norway (counties Finnmark and Trøndelag).

*Psectrocladius* (*Psectrocladius*) *psilopterus* was recorded from Spitsbergen by Lods-Crozet et al. [88]. We have examined a male adult from this study and can confirm the similarity with *Psectrocladius* (*P.*) *psilopterus*. However, there are slight differences in both colouration and hypopygial structures in a barcoded specimen of *P. psilopterus* from southern Norway that matches DNA barcodes of this species from Finland. The specimen we had on loan was unfortunately damaged in the return mail to the museum in Trento, Italy, but photos were taken before this shipment (available upon request). We consider *P. psilopterus* as absent from Svalbard, and regard the specimen recorded by Lods-Crozet, et al. [88] as belonging to a separate species (see *Psectrocladius* (*P*.) *borealis* below).

The species *Psectrocladius* (*Psectrocladius*) *ventricosus* has been included in checklists from Svalbard [93,98]. The record likely originates from Hirvenoja (1967) who reported the species from Lindholmhøgda in Adventdalen (Spitsbergen). Immature stages of *Psectrocladius* (*P*.) *ventricosus* have been recorded mostly from brackish or very hard water [62,123]; thus, it may be considered as unlikely to occur in the streams or ponds near Lindholmhøgda. Not all of Hirvenoja’s specimens could be located, but we have examined two males and these fit the diagnosis of *P* (*P*.) *limbatellus* except for having an antennal ratio of 2.2. The fact that Hirvenoja [33] reported *P*. (*P*.) *limbatellus* pupal exuviae (but no adults) from the nearby Isdammen, and that we have collected adult specimens of this species in the closely located Gruvedalen and Todalen, support our interpretation that the record of *P*. (*P*.) *ventricosus* was likely based on a misidentification. We therefore regard *P*. (*P*.) *ventricosus* as absent from Svalbard.

##### Key to Males

**Figure 54:**
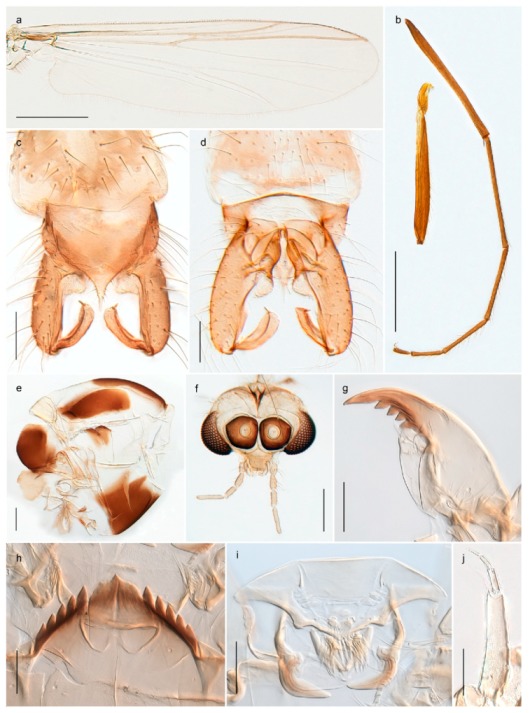
*Psectrocladius* (*Monopsectrocladius*) *calcaratus*. (**a**) Male wing, scale bar = 500 µm; (**b**) male fore leg, scale bar = 500 µm; (**c**) male hypopygium dorsal view, scale bar = 100 µm; (**d**) male hypopygium ventral view, scale bar = 100 µm; (**e**) male thorax, scale bar = 200 µm; (**f**) male head, scale bar = 200 µm; (**g**) larval mandible, scale bar = 50 µm; (**h**) larval mentum, scale bar = 50 µm; (**i**) larval labral surface, scale bar = 50 µm; (**j**) larval antenna, scale bar = 50 µm.

**Figure 55:**
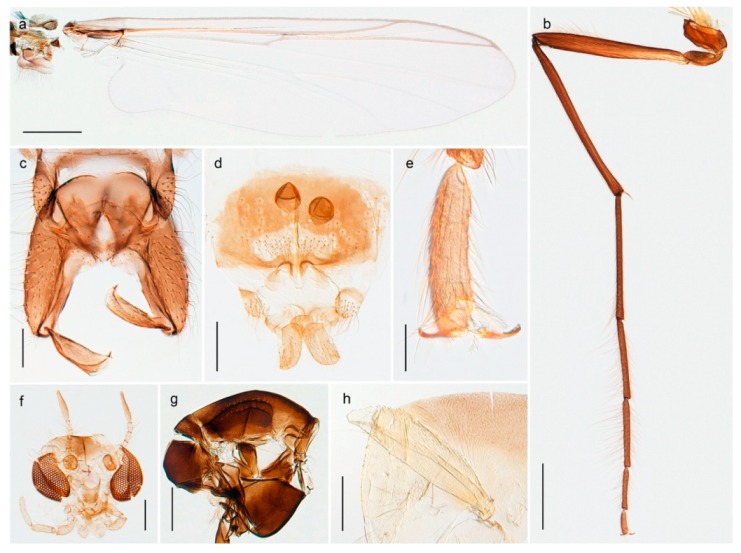
*Psectrocladius* (*Psectrocladius*) *barbimanus*. (**a**) Male wing, scale bar = 500 µm; (**b**) male fore leg, scale bar = 500 µm; (**c**) male hypopygium, scale bar = 100 µm; (**d**) female genitalia, scale bar = 200 µm; (**e**) male tarsomere 5, scale bar = 50 µm; (**f**) female head, scale bar = 200 µm; (**g**) male thorax, scale bar = 500 µm; (**h**) pupal thoracic horn, scale bar = 200 µm.

**Figure 56:**
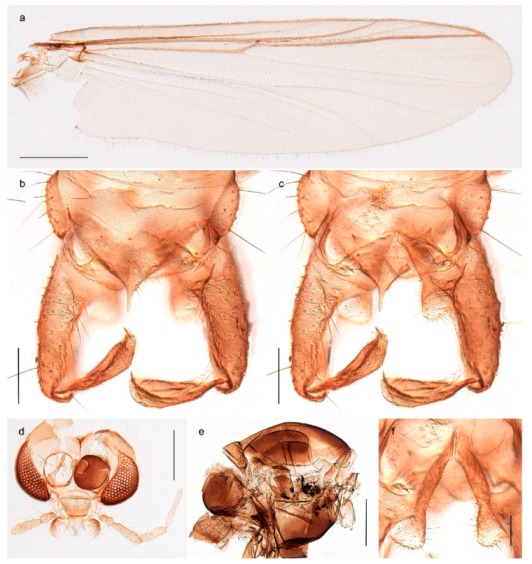
*Psectrocladius* (*Psectrocladius*) cf. *borealis*. (**a**) Male wing, scale bar = 500 µm; (**b**) male hypopygium, scale bar = 100 µm; (**c**) male hypopygium, scale bar = 100 µm; (**d**) male head, scale bar = 200 µm; (**e**) male thorax, scale bar = 500 µm; (**f**) male hypopygium, inner margin of gonocoxite, scale bar = 50 µm.

**Figure 57:**
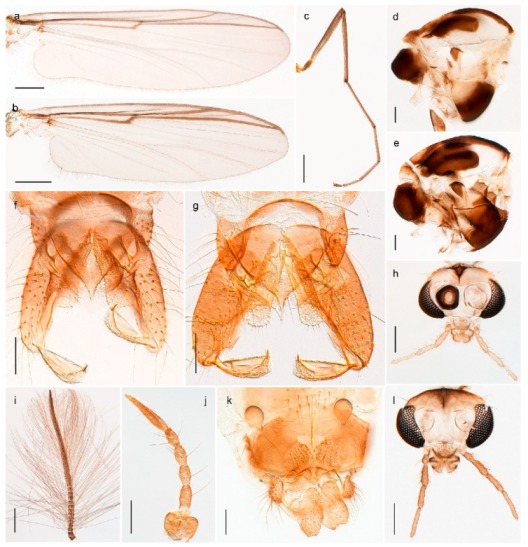
*Psectrocladius* (*Psectrocladius*) *limbatellus*. (**a**) Male wing, scale bar = 500 µm; (**b**) female wing, scale bar = 500 µm; (**c**) male fore leg, scale bar = 500 µm; (**d**) female thorax, scale bar = 200 µm; (**e**) male thorax, scale bar = 200 µm; (**f**) male hypopygium, scale bar = 100 µm; (**g**) male hypopygium, scale bar = 100 µm; (**h**) male head, scale bar = 200 µm; (**i**) male antenna, scale bar = 200 µm; (**j**) female antenna, scale bar = 100 µm; (**k**) female genitalia, scale bar = 100 µm; (**l**) female head, scale bar = 200 µm.

**Figure 58:**
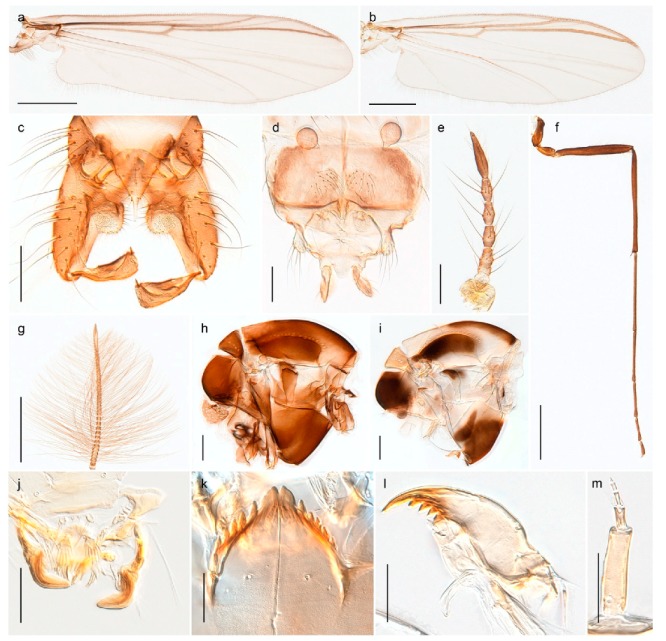
*Psectrocladius* (*Psectrocladius*) *oxyura*. (**a**) Male wing, scale bar = 500 µm; (**b**) female wing, scale bar = 500 µm; (**c**) male hypopygium, scale bar = 100 µm; (**d**) female genitalia, scale bar = 100 µm; (**e**) female antenna, scale bar = 100 µm; (**f**) male fore leg, scale bar = 500 µm; (**g**) male antenna, scale bar = 500 µm; (**h**) male thorax, scale bar = 200 µm; (**i**) female thorax, scale bar = 200 µm; (**j**) larval labral surface, scale bar = 50 µm; (**k**) larval mentum, scale bar = 50 µm; (**l**) larval mandible, scale bar = 50 µm; (**m**) larval antenna, scale bar = 50 µm.

1. Antero-median margin of gonocoxite with two emarginations, one of them far anterior, giving the free space between opposing gonocoxite contours a bottle-shaped appearance (Figure 54d)*Psectrocladius* (*Monopsectrocladius*) *calcaratus*- Antero-median margin of gonocoxite with one emargination at most, the far anterior always absent (e.g., Figure 57f,g) (Subgenus Psectrocladius)22. Several fore tarsomeres with long setae (tarsal beard), BR > 3.5 (Figure 55b)*Psectrocladius* (*P.*) *barbimanus*- Fore tarsus with shorter setae only, BR < 3.0 (Figures 57c and 58f)33. Anal point abruptly projecting from anal tergite; gonostylus narrow subapically (Figure 56b,c)*Psectrocladius* (*P.*) cf. *borealis*- Anal point not so abruptly projecting from anal tergite; gonostylus comparatively wider subapically (e.g., Figures 57f,g and 58c)44. Dorsocentral setae numerous (13–26), the most anterior setae situated anteriorly to adjacent lateral scutal band (vitta) (Figure 58h). Anal tergite gradually narrowed towards anal point, junction between the two difficult to define (Figure 58c)*Psectrocladius* (*P.*) *oxyura*- Dorsocentral setae fewer (5–15), the most anterior setae situated above lateral scutal band (Figure 57e). Anal tergite rounded posteriorly, its junction with the anal point more distinct (Figure 57f,g)55. Median margin of inferior volsella emaginated (figure 4d in [123])*Psectrocladius* (*P.*) *octomaculatus*- Median margin of inferior volsella more or less straight (Figure 57f,g, figure 5a in [123])*Psectrocladius* (*P.*) *limbatellus*

##### Preliminary Key to FeMales

This key is based on keys by Langton [63] and Sæther and Langton [124]. We have only seen females of *P* (*P*.) *barbimanus*, *P*. (*P*.) *limbatellus* and *P*. (*P*.) *oxyura*, thus could not test whether all characters used below will work on specimens from Svalbard. Additional characters that might be useful to separate females in *Psectrocladius* include the sizes and shapes of antennal flagellomeres as well as the pigmentation pattern on abdominal sternite VIII.
1. Sternite VIII posteriorly strongly emarginated in middle (often best seen in the shape of the apodeme). Seminal capsule pale*Psectrocladius* (*Monopsectrocladius*) *calcaratus*- Sternite VIII posteriorly weakly emarginated in middle. Seminal capsule golden or brown (subgenus *Psectrocladius*)22. Genitalia setose with about 40 setae on gonocoxite IX (Figure 55d)*Psectrocladius* (*P*.) *barbimanus*- Genitalia less setose, no more than 30 setae on gonocoxite IX (e.g., Figure 58d)33. Seminal capsule small, about 60 µm long; cercus short, about 100 µm long (Figure 58d)*Psectrocladius* (*P*.) *oxyura*- Seminal capsule larger, about 100 µm long; cercus longer, up to 200 µm (e.g., Figure 57k)44. Gonapophysis VIII broader than long (figure 3b in [63])*Psectrocladius* (*P*.) *octomaculatus*- Gonapophysis VIII as long as broad (Figure 57k, figure 3c in [63])*Psectrocladius* (*P*.) *limbatellus*

*Psectrocladius* (*Psectrocladius*) *barbimanus* (Edwards, 1929) is present in Svalbard and DNA barcodes of material from Spitsbergen clusters nicely with specimens from northern Norway (Finnmark) and northern Canada (Churchill).

The species *Psectrocladius* (*Psectrocladius*) cf. *borealis* Kieffer, 1919 was originally described from Spitsbergen in Kieffer and Thienemann [27] and later recorded by Edwards [28,29]. We have examined two adult male specimens from Edwards’ material that fit the (limited) original description by Kieffer (Figure 56), but given the observed morphological variability in *P*. (*P*.) *limbatellus* (see below), we cannot say with certainty if *P*. (*P*.) *borealis* constitute a separate species. We nevertheless choose to include the species as a separate entity in the key and figures. Material of *Psectrocladius* (*P*.) *psilopterus* recorded by Lods-Crozet et al. [88] from a Malaise trap near Bayelva on Spitsbergen (see comment above) apparently is conspecific with Edwards’ material of *Psectrocladius* (*P.*) *borealis*. The species is similar to *Psectrocladius* (*P*.) *psilopterus* and the related *P*. (*P*.) *bisetus* Goetghebuer, 1942 and *P*. (*P*.) *simulans* Johannsen, 1937, but is darker in colour and has a straight posterior margin of the inferior volsella. DNA barcodes from the Spitsbergen population are unavailable.

*Psectrocladius* (*Psectrocladius*) *limbatellus* (Holmgren, 1869) was originally described from Spitsbergen, but records have been reported from many countries in Europe, the Nearctic, the Near East and North Africa [86]. We have DNA barcodes from the Spitsbergen population that match those of specimens from Greenland and northern Canada (Churchill). Some of the matching specimens in BOLD from Greenland are currently identified as *P*. (*P*.) *barbimanus* and *P*. (*P*.) *sokolovae*, respectively (det. L. Paasivirta). Genetically divergent populations (>4% K2P distance) that are morphologically very similar to *P*. (*P*.) *limbatellus* have also been examined by us. These specimens are from northern Canada, central and northern Norway. Thus, we suspect that there currently are more than one species hiding under the name *P* (*P*.) *limbatellus* and that the nominal species has a narrower geographical distribution than present records indicate. At the same time there appears to be considerable morphological variation in the adult male hypopygia among specimens belonging to the same barcode cluster (Figure 57f,g). Thus, a revision including genetic characters is needed in order to clearly separate the closely related species of the *limbatellus* group. The females of the Spitsbergen population are larger than what has been described from England (also noted by Langton [63]) and more similar to *P*. (*P*.) *barbimanus* than previous identification keys indicate [63,124]. Thus, additional characters are introduced in the key above to accommodate differences observed in Spitsbergen material of both species.

*Psectrocladius* (*Psectrocladius*) *octomaculatus* Wülker, 1956 was reported by Langton [63] to be present in Edward’s material from Spitsbergen. We have seen the specimen examined by Langton and agree with the interpretation.

*Psectrocladius* (*Psectrocladius*) *oxyura* Langton, 1985 was described based on material from Cow Green Reservoir, England [63], but a wider distribution is indicated in the original description since at least part of the material identified as *P*. (*P*.) *limbatellus* by Brundin [47] and Wülker [62] fits the diagnosis of *P*. (*P*.) *oxyura*. We have examined and DNA barcoded populations from Bear Island and Spitsbergen that group with specimens from Canada and Greenland in BOLD.

#### 4.6.15. Smittia

Based on our review of the literature and available specimens (see below), the genus *Smittia* has three formally named species recorded from Svalbard. However, DNA barcode data indicate at least ten species from the archipelago and an additional two from Jan Mayen. Of these twelve species, ten are so far known as females only and we suspect that at least some of them are parthenogenetic. Most of the species can be separated by morphology and are keyed to species below. We have seen associated larvae of four of the seven unnamed species; in addition, we have associated larvae of *S. brevipennis* (Boheman, 1866), *S. extrema* (Holmgren, 1869) and *S. longicosta* (Edwards, 1922). We prefer to use interim names for species that we cannot assign to described and formally named taxa. Some of them might be facultative parthenogenetic with a wider distribution, and some might already be described as males. Thus, even before certain associations are made, we release the DNA barcodes and describe selected morphological characteristics to make the material available for future revisions.

*Smittia flexinervis* was described as adult male in *Thrichocladius* [sic!] from Bear Island [71]. We have not been able to locate the type material of this species and the latter cannot be identified or associated with recent material from the original description only. *Camptocladius longicosta* Edwards, 1922 was described from Bear Island based on 14 females [28]. The name is listed as a synonym of *Smittia flexinervis*, but it is not clear who originally suggested this synonymy [3,86]. It could have originated from Edwards’ (1922: p. 201) rather vague statement in the original description: “It is just possible that it may be the female of *C. flexinervis*, Kieff.” Given the high diversity of *Smittia* in the archipelago, this association is far from certain and we therefore regard *S. flexinervis* as a nomen dubium (see additional comment on *S. longicosta* below).

*Smittia spitzbergensis* (Kieffer, 1911) was described in *Thrichocladius* [sic!] based on a female from Hornsund, Spitsbergen [71]. From the drawing of the antenna, it clearly belongs to *Smittia*, but we are unable to associate the description with any of the morphotypes we have examined from the archipelago. The most similar species with regard to the antenna might be our *Smittia* sp. 7ES, but females of this species are considerably lighter in colour and have a shorter 5^th^ flagellomere and a shorter 5^th^ palpomere. We regard *S. spitzbergensis* as a nomen dubium until specimens can be associated with the original description and provide a better understanding of this species.

*Smittia lasiophthalma* was first described from Dubois, Illinois (USA) based on a female adult [125]. The record from Jan Mayen referred to in the World Catalogue [3] and Fauna Europaea [86] probably originates from Edwards’ questionable identification of Bristow’s material [35]. Edwards [30] also recorded this species with an uncertain identification from northern Spitsbergen (probably Reinsdyrflya). We have not compared Malloch’s types with Edwards’ material, but have examined a specimen from Edwards’ material in NHM labelled *Smittia* cf. *lasiophthalma*. This is conspecific with Smittia sp. 25ES discussed below. In light of the present knowledge of *Smittia* diversity in the Arctic, we regard it as unlikely that *Smittia lasiophthalma* is present on Svalbard and Jan Mayen.

*Smittia lasiops* was originally described in *Camptocladius* based on males and females collected near a house in Urbana, Illinois (USA) [125]. A female from the North Cape at Nordaustlandet on Svalbard was identified with some uncertainty by Edwards [29], which is likely the source for the species’ record for Svalbard in Lindegaard [98], Fauna Europaea [86] and the World Catalogue [3]. We regard the presence of *S. lasiops* on Svalbard as doubtful.

*Trichocladius polaris* Kieffer, 1919 has a type locality on Spitsbergen and is regarded as a subjective synonym of *Smittia extrema* (Holmgren, 1869) [3], likely based on Edwards’ (1924) observations: “I had wrongly identified this species. Holmgren’s specimens (16 ♀ from North Cape, but no ♂) are all quite evidently the species I recorded as *C. curvinervis*, Kieff.; if the determination was correct, Holmgren’s name will take precedence over Kieffer’s *curvinervis* and over Becher’s *incertus*, which has also proved to be the same species. Holmgren’s specimens have black halters, not yellow as in Becher’s and Kieffer’s material.” The observed differences in the colouration of the halters (described as white in the original description of *Trichocladius curvinervis* var. *polaris*) as well as differences in the female antenna (see figure 7 in Kieffer and Thienemann [27]) indicate that these two species names are not synonyms. Moreover, the figured antenna does not show the subapical setae typical for *Smittia* [27]. The reminder of the description fits the diagnosis of *Smittia*, however. Thus, we regard *T*. *curvinervis* var. *polaris* as a nomen dubium probably in *Smittia*. The species should not be confused with *Smittia polaris* (Kieffer, 1926) revisited by Sæther, et al. [126] from Greenland and northern Canada (Northwest Territories, Ellesmere Island). This species was originally described from Ellesmere Island [126,127]; records from Svalbard, Jan Mayen and Novaya Zemlya in Fauna Europaea [86] are of unknown origin and might be due to a mix-up of names.

##### Key to Males

1. Gonostylus distally strongly concave; hypopygium as in Figures 60c,f,g; AR >1.5; costal extension short, ending long before M1+2 (Figure 60a)
*Smittia extrema*
- Gonostylus distally weakly concave; hypopygium as in Figures 67g–i; AR <1.2; costal extension long, ending closer to M1+2 (Figure 67a)*Smittia* sp. 25ES

##### Key to FeMales

**Figure 59:**
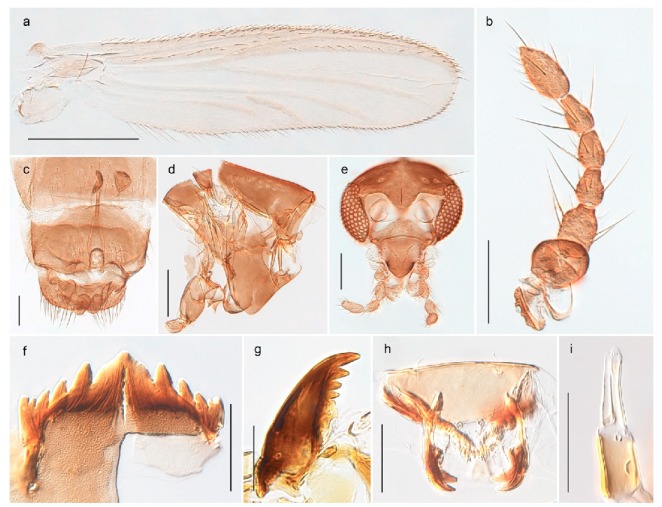
*Smittia brevipennis*. (**a**) Female wing, scale bar = 200 µm; (**b**) female antenna, scale bar = 100 µm; (**c**) female genitalia, scale bar = 100 µm; (**d**) female thorax, scale bar = 200 µm; (**e**) female head, scale bar = 100 µm; (**f**) larval mentum, scale bar = 50 µm; (**g**) larval mandible, scale bar = 50 µm; (**h**) larva labral surface, scale bar = 50 µm; (**i**) larval antenna, scale bar = 50 µm.

**Figure 60:**
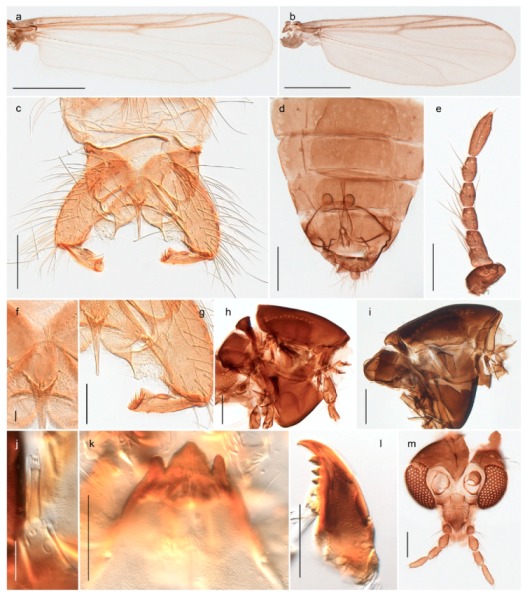
*Smittia extrema*. (**a**) Male wing, scale bar = 500 µm; (**b**) female wing, scale bar = 500 µm; (**c**) male hypopygium, scale bar = 100 µm (**d**) female genitalia, scale bar = 200 µm; (**e**) female antenna, scale bar = 100 µm; (**f**) male hypopygium inner structures, scale bar = 10 µm; (**g**) male anal point, gonocoxite and gonostylus, scale bar = 50 µm; (**h**) male thorax, scale bar = 200 µm; (**i**) female thorax, scale bar = 200 µm; (**j**) larval antenna, scale bar = 20 µm; (**k**) larval mentum, scale bar = 50 µm; (**l**) larval mandible, scale bar = 20 µm; (**m**) female head, scale bar = 100 µm.

**Figure 61:**
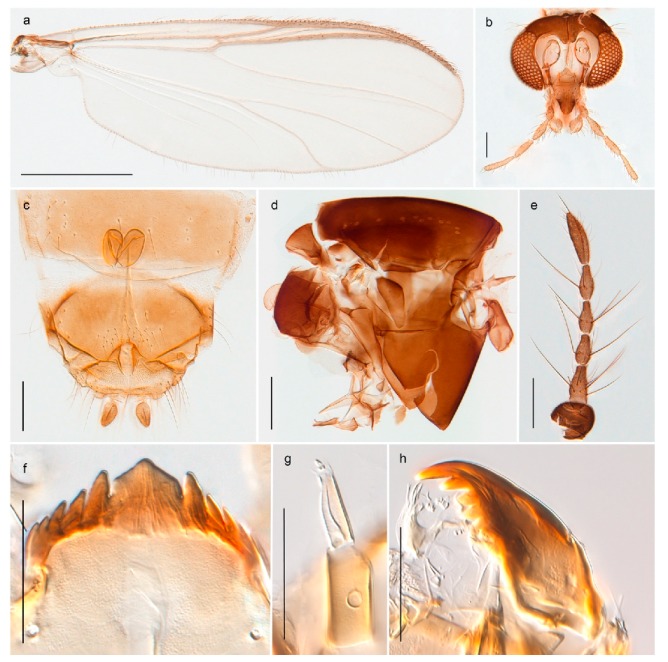
*Smittia longicosta*. (**a**) Female wing, scale bar = 500 µm; (**b**) female head, scale bar = 100 µm; (**c**) female genitalia, scale bar = 100 µm; (**d**) female thorax, scale bar = 200 µm; (**e**) female antenna, scale bar = 100 µm; (**f**) larval mentum, scale bar = 50 µm; (**g**) larval antenna, scale bar = 50 µm; (**h**) larval mandible, scale bar = 50 µm.

**Figure 62:**
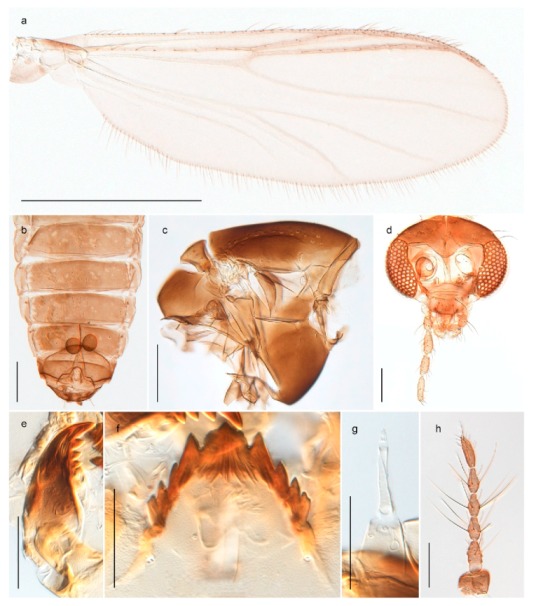
*Smittia* sp. 2ES. (**a**) Female wing, scale bar = 500 µm; (**b**) female genitalia, scale bar = 200 µm; (**c**) female thorax, scale bar = 200 µm; (**d**) female head, scale bar = 100 µm; (**e**) larval mandible, scale bar = 50 µm; (**f**) larval mentum, scale bar = 50 µm; (**g**) larval antenna, scale bar = 50 µm; (**h**) female antenna, scale bar = 100 µm.

**Figure 63:**
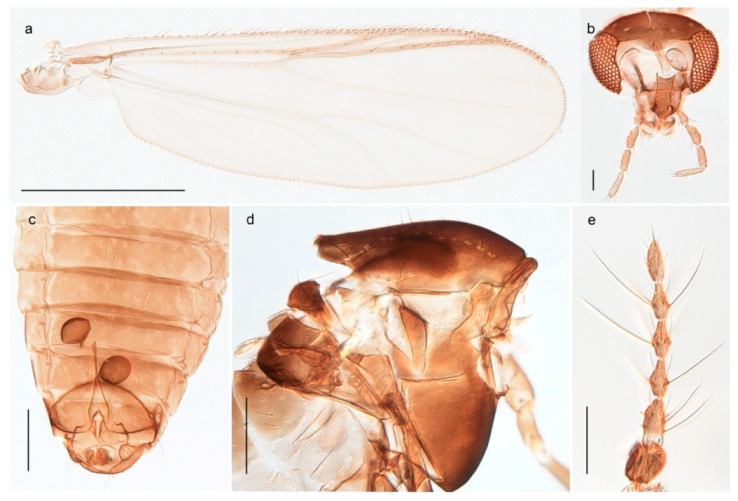
*Smittia* sp. 3ES. (**a**) Female wing, scale bar = 500 µm; (**b**) female head, scale bar = 100 µm; (**c**) female genitalia, scale bar = 200 µm; (**d**) female thorax, scale bar = 200 µm; (**e**) female antenna, scale bar = 100 µm.

**Figure 64:**
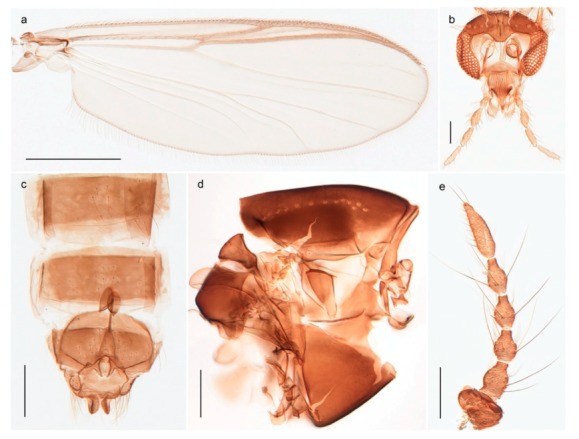
*Smittia* sp. 5ES. (**a**) Female wing, scale bar = 500 µm; (**b**) female head, scale bar = 100 µm; (**c**) female genitalia, scale bar = 200 µm; (**d**) female thorax, scale bar = 200 µm; (**e**) female antenna, scale bar = 100 µm.

**Figure 65:**
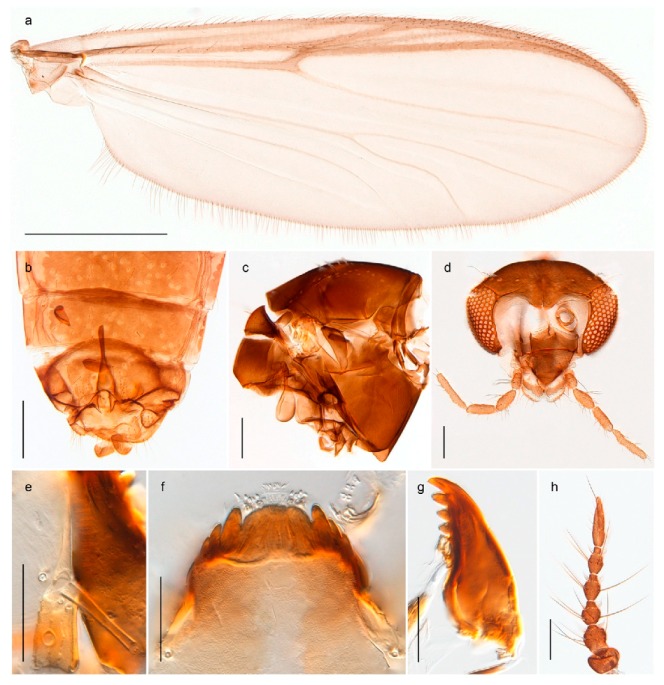
*Smittia* sp. 6ES. (**a**) Female wing, scale bar = 500 µm; (**b**) female genitalia, scale bar = 200 µm; (**c**) female thorax, scale bar = 200 µm; (**d**) female head, scale bar = 100 µm; (**e**) larval antenna, scale bar = 50 µm; (**f**) larval mentum, scale bar = 50 µm; (**g**) larval mandible, scale bar = 50 µm; (**h**) female antenna, scale bar = 100 µm.

**Figure 66:**
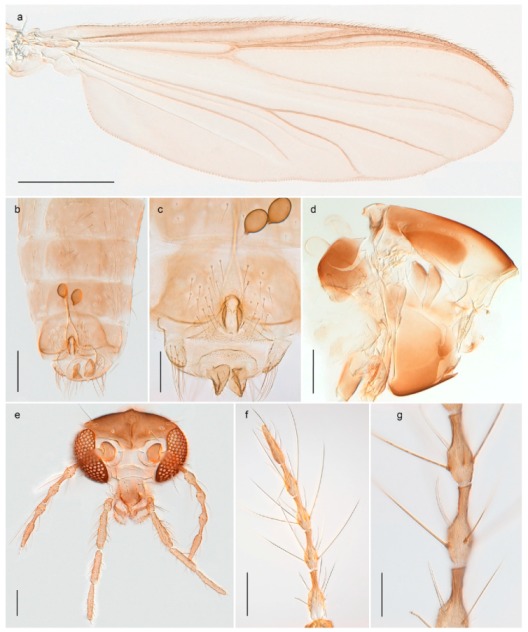
*Smittia* sp. 7ES. (**a**) Female wing, scale bar = 500 µm; (**b**) female genitalia, scale bar = 200 µm; (**c**) Female genitalia, detail, scale bar = 100 µm; (**d**) female thorax, scale bar = 200 µm; (**e**) female head, scale bar = 100 µm; (**f**) female antenna, scale bar = 100 µm; (**g**) female antenna, detail, scale bar = 50 µm.

**Figure 67:**
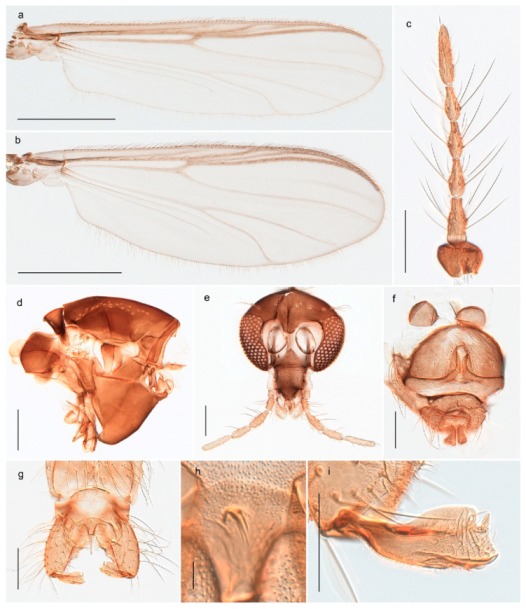
*Smittia* sp. 25ES. (**a**) Male wing, scale bar = 500 µm; (**b**) female wing, scale bar = 500 µm; (**c**) female antenna, scale bar = 100 µm; (**d**) female thorax, scale bar = 200 µm; (**e**) female head, scale bar = 100 µm; (**f**) female genitalia, scale bar = 100 µm; (**g**) male hypopygium, scale bar = 100 µm; (**h**) male hypopygium virga, scale bar = 10 µm; (**i**) male gonostylus, scale bar = 50 µm.

**Figure 68:**
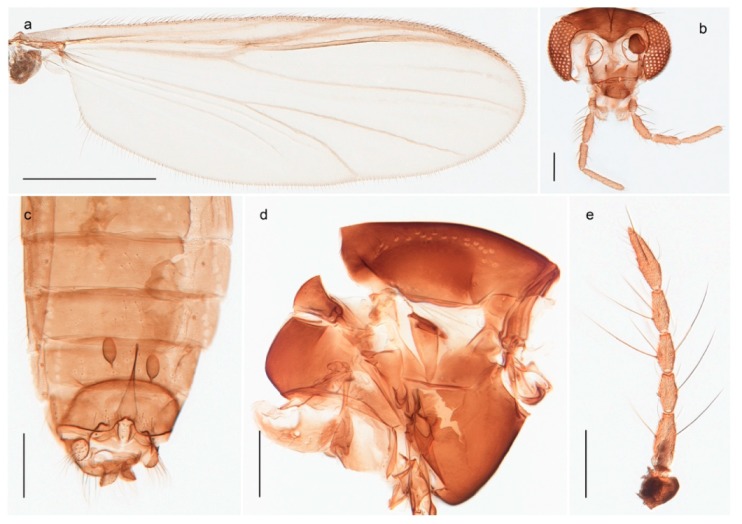
*Smittia* sp. 26ES. (**a**) Female wing, scale bar = 500 µm; (**b**) female head, scale bar = 100 µm; (**c**) female genitalia, scale bar = 200 µm; (**d**) female thorax, scale bar = 200 µm; (**e**) female antenna, scale bar = 100 µm.

**Figure 69:**
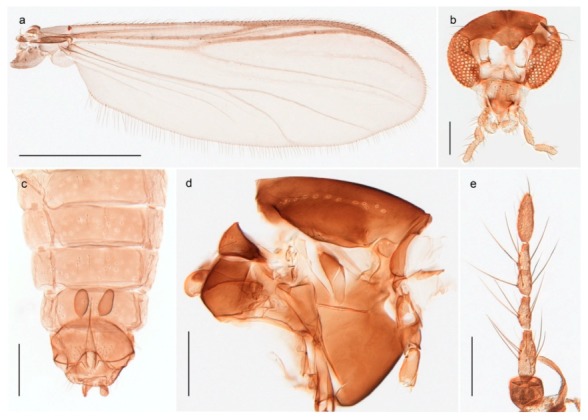
*Smittia* sp. 27ES. (**a**) Female wing, scale bar = 500 µm; (**b**) female head, scale bar = 100 µm; (**c**) female genitalia, scale bar = 200 µm; (**d**) female thorax, scale bar = 200 µm; (**e**) female antenna, scale bar = 100 µm.

**Figure 70:**
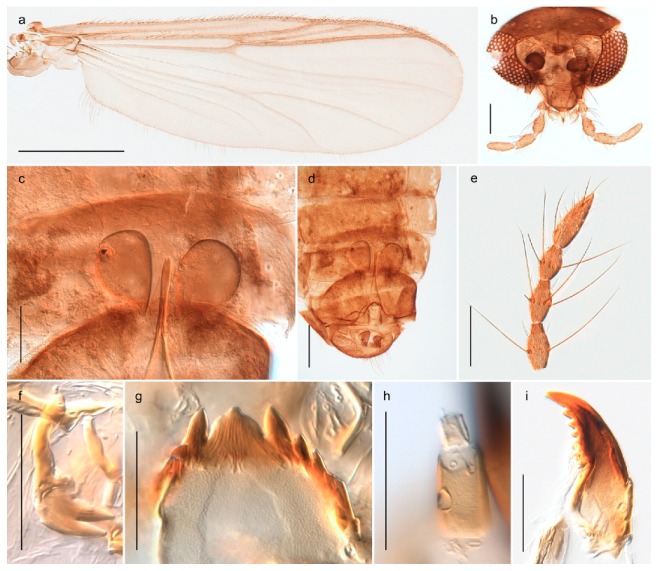
*Smittia* sp. 28ES. (**a**) Female wing, scale bar = 500 µm; (**b**) female head, scale bar = 100 µm; (**c**) female genitalia, scale bar = 200 µm; (**d**) female thorax, scale bar = 200 µm; (**e**) female antenna, scale bar = 100 µm.

1. Brachypterous or subapterous with indistinct veins and crossveins (Figure 59a)
*Smittia brevipennis*
- Macropterous with distinct veins and crossveins (e.g., Figure 61a)22. Antennal segments roundish (e.g., flagellum 2 length/width <1.5) with leaf like sensilla trichoidea broadened at base (Figures 64e and 65h)3- Antennal segments elongated (e.g., flagellum 2 length/width >1.5) with sensilla trichoidea narrow at base (e.g., Figure 66f,g)43. Length of sensilla trichoidea on antenna about 2/3 of length of antennal segments (Figure 64e); brown body colour*Smittia* sp. 5ES- Length of sensilla trichoidea on antenna about 1/2 of length of antennal segments (Figure 65h); black body colour*Smittia* sp. 6ES4. Light brown ground colour with dark vittae (Figure 66d); antennal flagellomeres long (flagellomere 2 length/width >3) (Figure 66f); wing length >1.9 mm*Smittia* sp. 7ES- Dark ground colour (e.g., Figure 67d); antennal flagellomeres shorter (flagellomere 2 length/width <3) (e.g., Figure 67c), wing length <1.955. Seminal capsule circular (e.g., Figure 60d)6- Seminal capsule oval (e.g., Figure 61c)106. Wing with costal extension ending clearly proximal of M1+2 apex (e.g., Figure 60b)7- Wing with costal extension ending almost above M1+2 apex (e.g., Figure 67b)97. Palpomere 4 short, less than two times longer than wide (Figure 60m); seminal capsule small, less than half length of segment VII
*Smittia extrema*
- Palpomere 4 long, more than two times longer than wide (Figures 62d and 63b); seminal capsule large, more than half length of segment VII88. Wing with anal lobe absent, fork FCu below RM (Figure 62a)*Smittia* sp. 2ES- Wing with anal lobe weak but present, fork FCu clearly distal to RM (Figure 63a)*Smittia* sp. 3ES9. Antennal flagellomeres 1–4 oval (Figure 70e); R4+5 almost straight (Figure 70a)*Smittia* sp. 28ES- Antennal flagellomeres 1–4 flask-shaped (Figure 67c); R4+5 curved towards costa (Figure 67b)*Smittia* sp. 25ES10. Palpomere 4 less than three times as long as broad, palpomere 5 less than four times as long as broad; seminal capsule longer than ½ length of segment VII (Figure 69c)*Smittia* sp. 27ES- Palpomere 4 more than three times as long as broad, palpomere 5 at least five times as long as broad; seminal capsule shorter than ½ length of segment VII (Figures 61c and 68c)1111. Wing fork FCu below RM (Figure 68a); neck of seminal capsule well sclerotized (Figure 68c)*Smittia* sp. 26ES- Wing fork FCu distal to RM (Figure 61a); neck of seminal capsule weakly sclerotized (Figure 61c)
*Smittia longicosta*


*Smittia brevipennis* (Boheman, 1856: p. 575) was originally described as *Chironomus brevipennis* from Kvalpynten on Edgeøya [25]. We have examined and DNA barcoded specimens from Russebukta (close to the locus typicus) as well as from other localities on Edgeøya and Spitsbergen and have associated larvae (Figure 59f–i) with the characteristic, brachypterous females. Sæther [57] redescribed the female genitalia of this species based on specimens from Siberia belonging to the Lundström collection.

*Smittia extrema* (Holmgren, 1869), originally described in both sexes from Nordaustlandet and Edgeøya [26], apparently has a wide Arctic distribution with records from Svalbard, Greenland, Canada and Russia [3]. DNA barcodes of the Svalbard population clusters with those of specimens from Zackenberg (Greenland), Nunavut and Northwest Territories. It is the only *Smittia* species from Svalbard with a recorded adult male as *Smittia* sp. 25ES has been found only on Jan Mayen, Greenland and in Canada. *Chironomus incertus* Becher, 1886 was originally described from Jan Mayen [34] and is listed as a questionable synonym of *Smittia extrema* in the World Catalogue of Chironomidae [3]. We have not examined the types (presumably in the Natural History Museum of Vienna), but based on Edwards’ observations [35], we agree that the species should be placed in *Smittia*. However, in light of the presence knowledge of the *Smittia* diversity on Svalbard and Jan Mayen, it is not clear whether *S. incerta* is conspecific with *S. extrema* or with *Smittia* sp. 25ES or with yet another established *Smittia* species. Nor do we know if males were included in the original description. We therefore regard *S. incerta* as a nomen dubium in *Smittia*.

The species *Smittia longicosta* (Edwards, 1922) was first described based on adult females from Walrus Bay on Bear Island [28]. We have examined one of Edwards’ syntypes and compared it with modern samples from the same area. We were also able to retrieve a 70bp mini barcode from the syntype that exclusively matches 100% with other barcoded specimens of *S. longicosta* from Svalbard. The species is identical to the one that [128] recorded under the interim name *Smittia* sp. 1ES. It is quite similar to what we call *Smittia* sp. 26ES, but the antennal flagellomeres 2–4 are slightly more bottle-shaped in *Smittia longicosta* and the seminal capsule necks are more sclerotized in *Smittia* sp. 26ES. The two species separate well by DNA barcodes (>12% pairwise distance) and a specimen of *S. longicosta* collected only 1.5 km from the locus typicus has been barcoded. *Smittia longicosta* has records from Spitsbergen, Edgeøya and Bear Island, while *Smittia* sp. 26ES appears to have a wider distribution with records in BOLD from Canada (Alberta and Nunavut), East Greenland, Jan Mayen and Spitsbergen.

*Smittia* sp. 5ES and *Smittia* sp. 6ES are similar in several features, e.g., both have a wing membrane with coarse punctation. They can be separated by the characters in the key; in addition, it appears that females of Smittia sp. 6ES have more robust flagellomeres (Figure 65h). The short and broad sensilla trichoidea of the female antenna in *Smittia* sp. 6ES resembles those in *Smittia aterrima* (Meigen, 1818), but DNA barcodes of *Smittia* sp. 6ES are more than 8% divergent from those we have associated with males of *S. aterrima* from central and northern Norway. *Smittia* sp. 5ES is recorded from Spitsbergen and Bear Island, and DNA barcodes match those of specimens in BOLD from Newfoundland, Canada. *Smittia* sp. 6ES has records from Spitsbergen and Edgeøya with barcode matches from East Greenland and Nunavut, Canada.

*Smittia* sp. 7ES has been recorded from Jan Mayen, Spitsbergen and Bear Island. The species is relatively easily separable from other *Smittia* on Svalbard as its females are more slender with longer extremities than in the other *Smittia*.

The remaining *Smittia* species from Svalbard also seem to have a relatively broad arctic to sub-arctic distribution (based on BIN-matches in BOLD): *Smittia* sp. 27ES is recorded from Jan Mayen, Finland and Switzerland; *Smittia* sp. 2ES from Spitsbergen, Arctic Canada (Nunavut) and East Greenland; *Smittia* sp. 3ES from Spitsbergen, Finland and Canada (Yukon, Northwest Terretories, Nunavut, Newfoundland); *Smittia* sp. 25ES from Jan Mayen, East Greenland and Canada (Nunavut); and *Smittia* sp. 28ES from Edgeøya and Nunavut, Canada.

#### 4.6.16. Tvetenia

**Figure 71:**
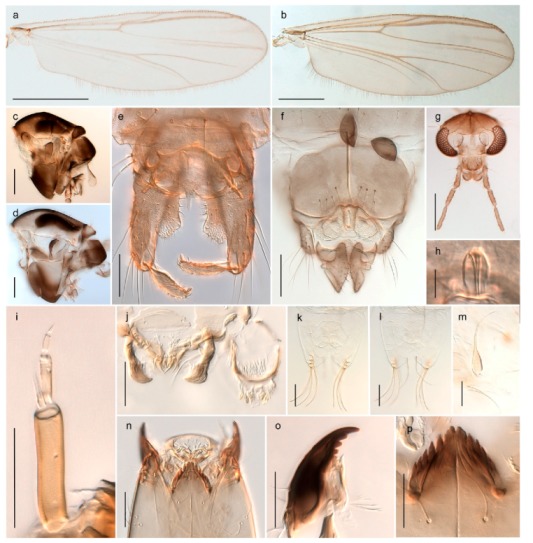
*Tvetenia bavarica*. (**a**) Male wing, scale bar = 500 µm; (**b**) Female wing, scale bar = 500 µm; (**c**) male thorax, scale bar = 200 µm; (**d**) female thorax, scale bar = 200 µm; (**e**) male hypopygium, scale bar = 50 µm; (**f**) female genitalia, scale bar = 100 µm; (**g**) female head, scale bar = 100 µm; (**h**) male hypopygium, virga, scale bar = 20 µm; (**i**) female antenna, scale bar = 50 µm; (**j**) larval labral surface, scale bar = 50 µm; (**k**–**l**) pupal anal lobe, scale bar = 100 µm; (**m**) pupal thoracic horn, scale bar = 100 µm; (**n**) larval head ventral view, scale bar = 50 µm; (**o**) larval mandible, scale bar = 50 µm; (**p**) larval mentum, scale bar = 50 µm.

*Tvetenia bavarica* has a wide distribution in the Holarctic Region and is here recorded from Svalbard for the first time. All specimens we have examined were from the warm springs Jotunkildene in Bockfjorden north on Spitsbergen, where we collected all major life stages (Figure 71). The DNA barcodes of the Spitsbergen population cluster with DNA barcodes from mainland Norway, Finland, Germany and Georgia in BOLD (BIN BOLD:AAD2063), including specimens collected near the locus typicus in Bavaria, Germany.

#### 4.6.17. Zalutschia

The species *Zalutschia tatrica* was listed as present on Svalbard by Lindegaard [98], but we have been unable to find the original documentation for this record. We have not seen any specimens of this species from the Archipelago either. In northern Europe, this species is recorded from northern Fennoscandia and the Kola Peninsula [53,129,130]. Until further documentation of this species becomes available, we regard it as not present on Svalbard and Jan Mayen (Figure 2, Figure 3, Figure 4, Figure 5, Figure 6, Figure 7, Figure 8, Figure 9, Figure 10, Figure 11, Figure 12, Figure 13, Figure 14, Figure 15, Figure 16, Figure 17, Figure 18, Figure 19, Figure 20, Figure 21, Figure 22, Figure 23, Figure 24, Figure 25, Figure 26, Figure 27, Figure 28, Figure 29, Figure 30, Figure 31, Figure 32, Figure 33, Figure 34, Figure 35, Figure 36, Figure 37, Figure 38, Figure 39, Figure 40, Figure 41, Figure 42, Figure 43, Figure 44, Figure 45, Figure 46, Figure 47, Figure 48, Figure 49, Figure 50, Figure 51, Figure 52, Figure 53, Figure 54, Figure 55, Figure 56, Figure 57, Figure 58, Figure 59, Figure 60, Figure 61, Figure 62, Figure 63, Figure 64, Figure 65, Figure 66, Figure 67, Figure 68, Figure 69, Figure 70 and Figure 71). 

## 5. Conclusions

Our review has confirmed that Svalbard has a surprisingly species-rich chironomid fauna. According to the records presented here, at least 74 species occur on Svalbard and Jan Mayen, with Spitsbergen as the most species-rich subregion (61 species). The high diversity on Spitsbergen compared to other regions in Svalbard (Table 1) almost certainly reflects that island’s large landmass and larger diversity of habitats, but also the fact that this is by far the best investigated island of the archipelago.

There likely are additional species still to be discovered in Svalbard and Jan Mayen. We document 20 distinct forms that do not fit current diagnoses for established species and therefore are given interim names. Although several of these might be associated with named species in the future via taxonomic revisions and associations of life stages, we are convinced that some also represent species new to science.

Our review shows that a number of previous species records from Svalbard were erroneus. We believe this reflects the lack of suitable identification keys for the region, but also the high level of taxonomic experience needed to correctly identify chironomids to species using morphology. The use of identification keys that were designed for other geographical regions easily leads to errors. Since it is easier to introduce new names in a checklist than to get rid of erroneously introduced records, we urge scientists to confirm their identifications against original descriptions and taxonomic reviews before publication.

DNA barcode data has been extremely important for our work. In several cases, we could only detect morphological differences to closely related species after DNA barcodes indicated distinct genetic lineages (e.g., *Orthocladius mixtus*). DNA barcodes have also been fundamental to associate life stages, especially when larvae fit poorly into established generic diagnoses (e.g., *Chaetocladius incertus*). We believe that the genetic characterisation of communities using DNA barcodes should always be integrated in morphological studies of Chironomidae, especially when sampling little- known regions. Only then is it possible to archive good comparative understanding of the true diversity and of how it relates to the distribution of similar taxa in other regions.

## Figures and Tables

**Figure 45:**
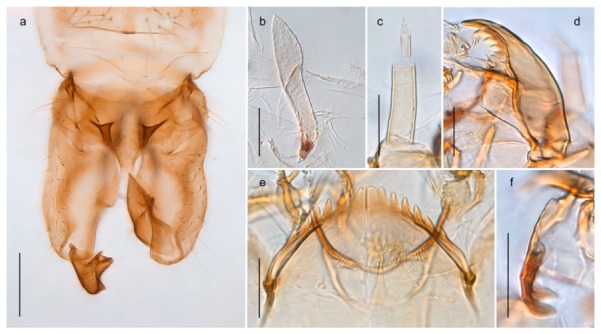
*Oliveridia tricornis*. (**a**) Male hypopygium, scale bar = 100 µm; (**b**) pupal thoracic horn, scale bar = 100 µm; (**c**) larval antenna, scale bar = 50 µm; (**d**) larval mandible, scale bar = 50 µm; (**e**) larval mentum, scale bar = 50 µm; (**f**) larval premandible, scale bar = 50 µm.

**Table 1:** Chironomidae (Diptera) species from Svalbard and Jan Mayen with associated DNA barcode records. Specimen records refer to material vouchered in museum collections. Taxa marked with ‘*’ are included in the discussion, x = material examined or analysed by the authors, [#] = reference to literature record if known from the literature only.

Species	Specimen Records				DNA Barcodes		Figure
	Spits-Bergen	Bear Island	Edge Island	Jan Mayen	Svalbard/Jan Mayen	Other Regions	
**Podonominae**							
*Parochlus kiefferi* (Garrett, 1925) *						x	2
**Tanypodinae**							
*Arctopelopia melanosoma* (Goetghebuer, 1933) *		x			x	x	3
*Procladius frigidus* (Holmgren, 1869) *	x	x			x	x	4
**Diamesinae**							
*Diamesa aberrata* Lundbeck, 1898	x	x		[35,51]	x	x	5
*Diamesa arctica* (Boheman, 1866) *	x				x	x	6
*Diamesa bertrami* Edwards, 1935 *	x	[51]			x	x	7
*Diamesa bohemani* Goetghebuer, 1932	x	x			x	x	8
*Diamesa hyperborea* Holmgren, 1869 *	[70]	x			x	x	9
*Pseudokiefferiella* sp. 1ES *	x				x	x	10
**Chironominae, Tanytarsini**							
*Micropsectra insignilobus* Kieffer, 1924	x				x	x	11
*Micropsectra logani* (Johannsen, 1928)		x			x	x	12
*Micropsectra radialis* Goetghebuer, 1939	x	x			x	x	13
*Paratanytarsus austriacus* (Kieffer, 1924)	x	x			x	x	14
*Tanytarsus heliomesonyctios* Langton, 1999 *	x	x			x	x	15
**Chironominae, Chironomini**							
*Chironomus (C.) islandicus* (Kieffer, 1913) *		x			x	x	16
*Chironomus* (*C.*) *lugubris* Zetterstedt, 1850	x				x		17
*Chironomus* (*C.*) sp. 1TE *	x				x	x	18
*Sergentia coracina* (Zetterstedt, 1850) *						x	19
*Stictochironomus psilopterus* (Edwards, 1935) *		x			x		20
**Orthocladiinae**							
*Allocladius* sp. 1ES *	x		x		x	x	21
*Bryophaenocladius* sp. 5ES *	x				x	x	22
*Chaetocladius holmgreni* (Jacobson, 1898) *	x	x		x	x	x	23
*Chaetocladius incertus* (Lundström, 1915) *	x				x	x	24
*Chaetocladius* sp. 8ES *	x				x		24
*Corynoneura* sp. 1ES *	x				x	x	25
*Cricotopus* (*C.*) *gelidus* (Kieffer, 1922) *	x				x		26
*Cricotopus* (*C.*) *lestralis* (Edwards, 1924) *	x				x		27
*Cricotopus* (*C.*) *pilosellus* Brundin, 1956 *		x			x	x	28
*Cricotopus* (*C.*) *tibialis* (Meigen, 1804) *	x	x		x	x	x	29
*Cricotopus* (*C.*) *villosus* Hirvenoja, 1973 *						x	30
*Cricotopus* (*I.*) *glacialis* Edwards, 1922 *	x				x	x	31
*Heterotrissocladius subpilosus* (Kieffer, 1911) *		[71]				x	32
*Hydrobaenus conformis* (Holmgren, 1869)	x				x		33
*Hydrosmittia oxoniana* (Edwards, 1922) *	x	x			x	x	34
*Hydrosmittia* sp. 1ES *	x				x	x	34
*Limnophyes brachytomus* (Kieffer, 1922) *	x	x			x	x	35
*Limnophyes eltoni* (Edwards, 1922) *	x	x			x	x	36
*Limnophyes pumilio* (Holmgren, 1869) *	x		x		x	x	37
*Limnophyes schnelli* Sæther, 1990 *		x			x	x	38
*Metriocnemus brusti* Sæther, 1989 *	x		x		x	x	39
*Metriocnemus cataractarum* Kieffer, 1919 *	[27,30]						
*Metriocnemus eurynotus* (Holmgren, 1883) *	x	x			x	x	40
*Metriocnemus fuscipes* (Meigen, 1818) *	x	x			x	x	41
*Metriocnemus ursinus* (Holmgren, 1869) *	x		x	x	x	x	42
*Metriocnemus* sp. 1ES *	x	x	x		x	x	43
*Metriocnemus* sp. 8ES *	x				x		44
*Oliveridia tricornis* (Oliver, 1976)	x				x	x	45
*Orthocladius* (*Eudact.*) *almskari* Sæther, 2004 *	[58]						
*Orthocladius* (*Eudact.*) *gelidorum* (Kieffer, 1923) *	x	x			x	x	46
*Orthocladius* (*Eudact.*) sp. 2TE *		x			x		47
*Orthocladius* (*Euorth.*) *telochaetus* Langton, 1985 *	x				x	x	48
*Orthocladius* (*O.*) *decoratus* (Holmgren, 1869) *	x	x			x	x	49
*Orthocladius* (*O.*) *knuthi* Soponis, 1977 *	[72]					x	
*Orthocladius* (*O.*) *mixtus* (Holmgren, 1869) *		x			x	x	50
*Orthocladius* (*O.*) *nitidoscutellatus* Lundström, 1915 *	x	x			x	x	51
*Orthocladius* (*P.*) *consobrinus* (Holmgren, 1869) *	x	x			x		52
*Paraphaenocladius brevinervis* (Holmgren, 1869) *	x				x	x	53
*Psectrocladius* (*M.*) *calcaratus* (Edwards, 1929) *						x	54
*Psectrocladius* (*P.*) *barbimanus* (Edwards, 1929) *	x				x	x	55
*Psectrocladius* (*P.*) cf. *borealis* Kieffer, 1919 *	x						56
*Psectrocladius* (*P.*) *limbatellus* (Holmgren, 1869) *	x				x	x	57
*Psectrocladius* (*P.*) *octomaculatus* Wülker, 1956 *	[63]					x	
*Psectrocladius* (*P.*) *oxyura* Langton, 1985 *	x	x			x	x	58
*Smittia brevipennis* (Boheman, 1866) *	x		x		x		59
*Smittia extrema* (Holmgren, 1869) *	x		x		x	x	60
*Smittia longicosta* (Edwards, 1922) *	x	x	x		x		61
*Smittia* sp. 2ES *	x				x	x	62
*Smittia* sp. 3ES *	x				x	x	63
*Smittia* sp. 5ES *	x				x	x	64
*Smittia* sp. 6ES *	x		x		x	x	65
*Smittia* sp. 7ES *	x	x		x	x		66
*Smittia* sp. 25ES *				x	x	x	67
*Smittia* sp. 26ES *	x			x	x	x	68
*Smittia* sp. 27ES *				x	x	x	69
*Smittia* sp. 28ES *			x		x	x	70
*Tvetenia bavarica* (Goetghebuer, 1934) *	x				x	x	71
